# Health Promotion and Disease Prevention Interventions for the Elderly: A Scoping Review from 2015–2019

**DOI:** 10.3390/ijerph17155335

**Published:** 2020-07-24

**Authors:** Ching-Ju Chiu, Jia-Chian Hu, Yi-Hsuan Lo, En-Yu Chang

**Affiliations:** 1 Institute of Gerontology, College of Medicine, National Cheng Kung University, Tainan 70101, Taiwan; g60435g@yahoo.com.tw; 2 School of Pharmacy, College of Medicine, National Cheng Kung University, Tainan 70101, Taiwan; panda20220@gmail.com; 3 Department of Statistics, College of Management, National Cheng Kung University, Tainan 70101, Taiwan; tiffanyl1999@gmail.com

**Keywords:** elderly, scoping review, health promotion, eHealth

## Abstract

In this study, a scoping review method is used to review the distribution and trends in health promotion research and explore the use and contribution of eHealth technologies in health promotion in the elderly. The study includes six search databases: PubMed, CINAHL, the CochraneLibrary, EMBASE, PubPsych, and ERIC (EBSCOhost), and studies published from January 2015 to October 2019, written in English, were included and analyzed. The findings of the study reveal that the amount of literature on promoting health for the elderly has increased, and some specific types of interventions are still favored in current health promotion efforts for older adults. The most commonly used methods were found to be health promotion (*n* = 322), followed by screening (*n* = 264), primary prevention (*n* = 114), and finally social support (*n* = 72). Beyond the above interventions, eHealth technology is also used in health promotion activities to prevent the elderly from falling and to improve home safety, etc. However, although the application of eHealth technology has been applied in areas such as fall prevention, mental health promotion, and home security monitoring, it is still immature, and thus more rigorous research is needed in different areas, especially in older populations, various professions, women, and people with dementia.

## 1. Background

According to the World Health Organization, health promotion enables people to take more control over their health. It covers a wide range of social and environmental interventions designed to benefit and protect everyone’s health and quality of life by addressing and preventing the causes of poor health, rather than just focusing on treatment and cures [1]. It can help people to manage their physical and psychological conditions, improve personal, family, and social health, and improve quality of life. It also increases the average healthy life expectancy and reduces unnecessary medical expenses and waste [2].

However, the promotion of health in the elderly is not based on a single aspect of health. It covers a wide range of issues, including nutrition, sleep, disability, and obesity. The elderly differ from the young in many ways. For example, according to a 2018 Taiwan elderly status survey report, the rate of self-reported chronic diseases among those aged over 65 is 64.88%. Therefore, in the formulation of policy or research, the special characteristics of diseases as well as the physical and mental needs of the elderly must be taken into account [3]. According to the World Health Organization, 20% of the world’s population will be over the age of 60 by 2050. The soaring incremental increase in the elderly population has made advanced health promotion an important issue.

Due to the wide range of entry points for that study of health promotion in the elderly, Duplaga et al., published a study on the promotion of health in the elderly in 2016 [4], which systematically summarized and reviewed research published on this topic from January 2000 to April 2015 and used a scoping review to systematically collate reviews. Although both scoping reviews and literature reviews contain reviews of empirical literature, their purposes and focuses of discussion are different. A scoping review mainly involves the collection of all relevant material to provide rather extensive coverage of a topic in order to provide an integrated discussion and report the relevant research designs and methodologies. The main concepts in the topic are determined in order to provide a complete system and valid comparability. A literature review, on the other hand, focuses on only one research problem and collects the most relevant and least relevant studies to review. In terms of data analysis, a scoping review describes the literature as a whole, while a literature review evaluates a single topic and empirically extracts the relevant research results.

Mariusz Duplaga’s research covered studies conducted from 2001 to 2015, where research over a five year period was collected to further explore the current technologies used to promote heath in the elderly so that health management programs for the elderly could be constructed, and valid policy suggestions could be made based on the elderly at different ages and states of health. Based on this research, the current study further explores whether the trends in and distribution of the literature on health promotion in the elderly has changed since 2015. Different from Duplaga et al., this study focuses on the role and application of eHealth technologies in health promotion in the elderly. eHealth is the use of ICT with cost-effective, secure methods in order to support health and health-related fields, including health-care services, health surveillance, the health literature, and health education, knowledge, and research [5]. Using ICT technologies can make it possible for the elderly to acquire effective health services faster and more efficiently. In addition, the WHO also listed eHealth as a priority for project development, which infers that eHealth has a great potential in the area of elderly health promotion. Therefore, it is important to search the usage and results of eHealth technologies in existing research. The objectives for this study are as follows: (1) to investigate the distribution of and trend in the secondary literature on health promotion in the elderly over a recent five year period, (2) to explore the use and contribution of eHealth technology in the secondary literature on health promotion in the elderly, and (3) to make suggestions for the development of health policies for the elderly in the future.

## 2. Method

The present study was based on scoping review methodologies designed in the last five years (2015–2019), with the purpose of identifying and reviewing the effectiveness of different interventions addressing elderly health promotion and related areas. This study is a continuation of the research of Duplaga et al. [4]. The concept of the scoping review in the present study follows that of previous scoping review studies, such as Arksey and O’Malley [6], and further discussion by Levac, Colquhoun, and O’Brien et al. [6]. Arksey and O’Malley stated six steps for conducting a scoping review. The first step was to identify the purpose of the research. The purpose of this study is to provide a scoping review of health promotion and a variety of interventions for elderly people in a recent five year period (2015–2019). Based on the Population-Concept-Context (PCC) mnemonic for a scoping review, the study population (P) is elderly people; the concept (C) is health promotion, and the context (C) is related systematic reviews and/or meta-analysis in a recent five year period.

### 2.1. Search Strategy

The search strategy for this study followed the PCC context and the concept of health promotion based on a previous study conducted by Duplaga et al. (2016) [7] using a combination of keywords shown in Table 1. The search strategy included six search databases: *PubMed, CINAHL, the CochraneLibrary, EMBASE, PubPsych, and ERIC (EBSCOhost)*, and the included reviews were published in English from January 2015 to October 2019.

### 2.2. Searching Strategy Process

The search process included the following steps: (1) searching for keywords from all six databases, (2) screening titles, (3) screening abstracts, (3) screening full reports, and (4) adding the remaining studies into this scoping review study.

### 2.3. Data Extraction and Assessment

The reviews in this study were classified into different categories based on previous studies, including the year of publication, database, the age, sex, and country of the targeted audience, intervention types, and the target of the interventions. Beyond the categories shown in Table 1, this scoping review included another category, “eHealth technology applications”, for the purpose of further discussion of the trend in eHealth technology applications in an effort to determine what types of technologies are more useful and how they work in the area of health promotion in the elderly.

The classification process was conducted by two authors independently, with each of them overseeing three databases. Divergent opinions were resolved on a consensus basis. If a consensus was not reached, a third author was referred to for the final decision.

The data collection tools used in this study were EndNote X9 and Excel. After downloading and duplicating all the reviews in EndNote, we imported the final data into Excel and began the screening process. The final results after the screening process include 2 reviews in CINAHL, 8 reviews in Cochrane, 143 reviews in Embase, 19 reviews in Eric host, 207 in reviews PubMed, and 107 reviews in PubPsych, with a total of 486 reviews.

The screening and classifying processes were completed by 23 March 2020, and the figures were plotted with Excel using the list of data.

We provided definitions and the related provenance of different items in order to give a clearer understanding (Supplementary 1), and we used the PRISMA-SCR checklist containing 20 essential reporting items to provide more information. (Supplementary 2)

## 3. Results

**Figure 1:**
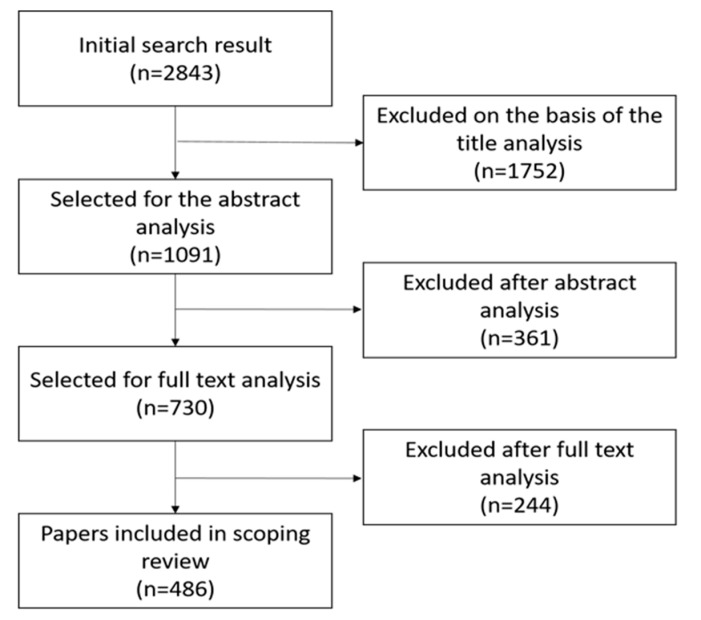
Scoping review search strategy.

We reviewed 2843 studies after searching the six databases, retaining 1091 studies after screening the titles, 730 after screening the abstracts, and ultimately retaining 486 studies for the scoping review study. The process diagram is shown in Figure 1. We provide a list of the 486 reviews included in the study and the title, year of publication, population, gender, general area of intervention, targeted area, and references. The list is ordered by title. (Supplementary 3)

### 3.1. Numbers of Reviews Sorted by Years

**Figure 2:**
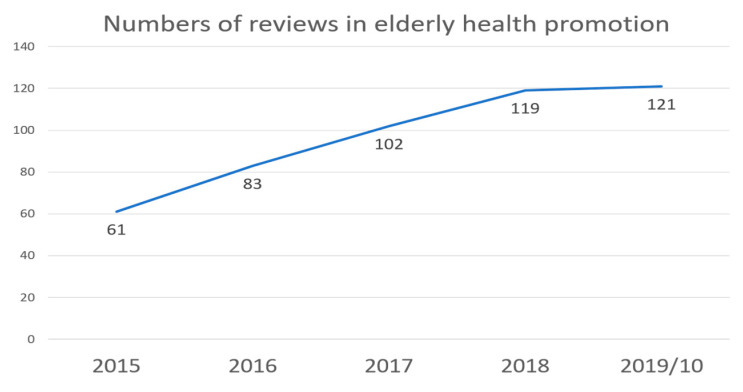
Number of reviews from 2015 to 10 October 2019.

Figure 2 shows that from 2015 to October 2019, the number of studies related to elderly health promotion increased annually, from 61 studies in 2015 to 121 studies in 2019, where there were more reviews in the first ten months of 2019 than in the entire year of 2018. It can be inferred from this that an increasing number of scholars are paying attention to issues related to the health of the elderly and related topics, such as assisting technologies, etc.

### 3.2. Gender

Most of the studies in this scoping review did not have a specific gender target. Only 10 out of the 486 studies focused specifically on elderly females, and there was no research focusing only on males.

### 3.3. Intervention Methods

The classification for the interventions methods followed Duplaga et al. [4] and was divided into four categories: health promotion (HP), primary prevention (PP), screening (SC), and social support (SS).

**Figure 3:**
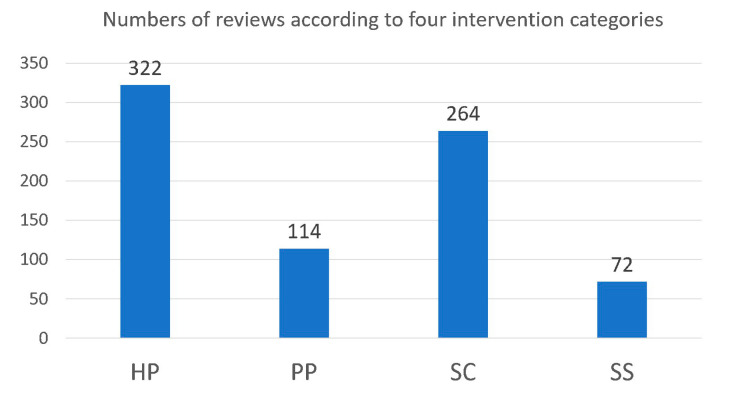
Number of reviews based on four intervention categories.

The most common method was health promotion, where 322 out of the 486 reviews (66%) were classified as health promotion (HP) interventions. Meanwhile, 264 (54%) were classified as screening (SC), 114 (23%) were classified as primary prevention (PP), and 72 (15%) were classified as social support (SS), as shown in Figure 3. Since studies may include more than one intervention method, the total number of studies exceeded the number of reviews.

**Figure 4:**
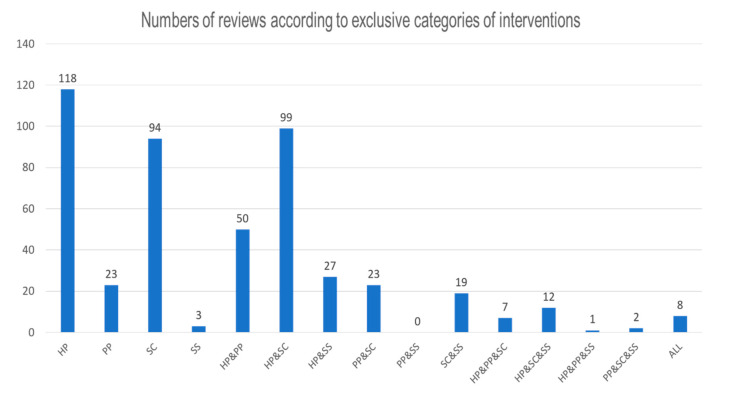
Number of reviews according to specific intervention categories.

If every combination was considered to be an independent category, the most common intervention method was health promotion (*n* = 118, 24.3%), and the second most common method was health promotion and screening (*n* = 99, 20.4%), for which the results are shown in Figure 4.

### 3.4. Areas Targeted for Health Promotion

The areas targeted for health promotion were classified as “disease-oriented,” “physical activity,” “general health,” “quality of life,” “frailty,” “cognitive function,” “mental health,” “nutrition,” “disability,” “independence,” “sleep quality,” “psychosocial functioning,” and “addiction.”

**Figure 5:**
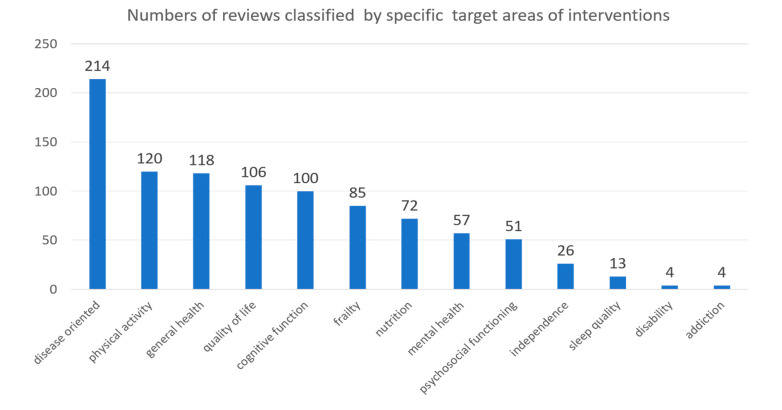
Number of reviews classified by specific intervention target area.

Figure 5 shows that the most common target for the review studies was “disease-oriented” (*n* = 214, 44% of all 486 reviews), followed by “physical activity” (*n* = 120, 24.7%), “general health” (*n* = 118, 24.2%), “quality of life”(*n* = 106, 21.8%), “cognitive function” (*n* = 100, 20.5%), “frailty”(*n* = 85, 17.5%),“nutrition”(*n* = 72, 14.8%), “mental health”(*n* = 57,11.7%), “psychosocial functioning”(*n* = 51,10.5%), “independence”(*n* = 26, 5.4%), “sleep quality”(*n* = 13, 2.7%), and finally, “addiction”(*n* = 4, 0.8%) and “disability” (*n* = 4, 0.8%). Since every study may consider more than one area, the total number of studies exceeded the number of reviews.

### 3.5. EHealth Technology Applications

Twelve out of the 486 reviews show the use of eHealth technology as an interventive tool for elderly health promotion. The technologies reviewed in the present work included “virtual reality” (*n* = 3), “smart homes and home health monitoring technologies” (*n* = 1), “socially assistive robots” (*n* = 5), and “electronic assistive technology” (*n* = 3). In addition, according to the statistical results, involvement in eHealth technology is also experiencing a slightly increasing trend.

## 4. Discussion and Future Directions

In this study, studies in the field of elderly health promotion from 2015 to October 2019 were summarized using a scoping review to investigate the research trends and distribution in the past five years in order to compare the results with studies compiled by Duplaga between 2000 and 2015. In this study, it was found that studies on health promotion in the elderly have increased annually. The topic of health promotion in the elderly is clearly receiving increasing scholarly attention.

A total of 486 articles from 2015 to 2019 were included in our study, with an average of 97 articles per year, compared with the 334 articles included by Duplaga in the 15 years from 2000 to 2014, with an average of 23 articles per year. From the above statistics, it can be seen that there has been a significant increase in the research on health promotion in the elderly. We did not find any studies specifically targeted at a single gender. Of the 486 studies, only 10 (2.1%) were found on older women, and the proportion of gender-specific studies in Duplaga was also small. Only 33 (9.9%) of the 486 studies were gender-specific. Therefore, it was found that health promotion in the elderly covers all genders at present, which means that in the future, gender effects and effects associated with different groups are worthy of study.

According to the research results for intervention methods, the most commonly used research methods for health promotion in the elderly during the period under observation included health promotion (HP), screening (SC), primary prevention (PP) and social support (SS), and many systematic reviews and meta-analyses used two or more types of intervention methods for health promotion in the elderly. When each combination was examined separately, the top five found in this study were “Health promotion,” “Health promotion and Screening,” “Screening,” “Health promotion and Primary prevention,” and “Health promotion and Social support.” Based on these results, it was found that health promotion accounts for a significant proportion of the issues related to health promotion in the elderly, indicating that the issue of health promotion for the elderly is currently under active investigation. Compared with the results of Duplaga’s research, where primary prevention accounted for the majority of the issues, it can be inferred that the current issue of health promotion is not limited to primary prevention, but is being studied in a multi-faceted manner with a more diverse range of topics and interventions. To compare the present study with that conducted by Duplaga et al. [4], we provide a table (Supplementary 4) including a comparison of the aims, numbers of studies included, publication years, databases, target areas of interventions, general area of interventions, specific gender target, and classification differences.

Based on the results, purpose-oriented studies can be ranked in the following order: disease-oriented, physical activity, general health, quality of life, general health, frailty, nutrition, mental health, psychosocial functioning, independence, sleep quality, disability, and addiction. The classification results also reveal a trend in multi-purpose-oriented discussions in some of the studies, including: disease-oriented vs. physical activity, physical activity vs. general health, quality of life, cognitive function, disease-oriented vs. nutrition, general health vs. general health, etc. This phenomenon suggests that the issue of health promotion in the elderly needs to be studied from many perspectives, rather than discussed as a single topic. It is also worth noting that the results of the author’s classifications are consistent with Duplaga’s top four results, with the first issue in all studies being treating and preventing disease, followed by physical activity, general health, and quality of life. The following is a discussion of the top five categories.

Among the purpose-oriented classifications, those related to diseases predominated and covered a relatively wide range, such as knee arthritis or sarcopenia of the musculoskeletal system, Alzheimer’s disease, and Parkinson’s disease. Psychological disorders included depression, bipolar disorder, post-traumatic stress disorder, and delirium. Chronic diseases included diabetes, hypertension, and kidney disease. Other illnesses included anorexia and dry mouth. Multiple complications were also the target of some studies. The authors in the literature attempted a variety of interventions, such as medication, physical activity, diet plans, and aromatherapy, to slow or even cure age-related illnesses. Due to the high heterogeneity of health status among the elderly and the significant influence of diseases on health promotion in the elderly, it is necessary to consider the influence of disease during the health promotion of the elderly and to create a health promotion policy based on a multi-dimensional assessment. Therefore, in the future, more in-depth discussion and research on specific diseases would certainly lead to remarkable results in the promotion of health in the elderly.

The physical activity category was the second largest among the purpose-oriented categories. It mainly focuses on sports, such as aerobic exercise, dancing, Tai-Chi, yoga, water aerobics, etc. However, some studies investigated digital behavior interventions, Pilates training, resistance training, physical activity monitoring, etc. In terms of type, most of the studies were related to disease, aging, and cognitive function, so physical activity was used fairly often as an intervention. In addition, Hu et al. conducted a scoping review in 2019 on the involvement of elderly people in physical activities as interventions who did not receive adequate medical services. The results suggest that interventions that are appropriate to the characteristics of older persons with disabilities should be tailored to their race, socioeconomic status, and level of physical disability [8]. It was observed that the ethnicity, socio-economic status, cultural characteristics, and diseases of the target subjects should be considered as factors in the design of interventional physical activities for the elderly.

The general health category was the third largest among the purpose-oriented categories and covered a wide range of areas, typically including physical functioning, role functioning, social functioning, mental health, health perception, and pain [9]. Therefore, if the intervention method discussed in each study contained three or more of the above conditions, it was included in this classification. From the classification results, it was found that there were more studies focusing on multi-field comprehensive analyses, while the analysis of a single factor tended to decrease over the period under consideration. For example, in this category, some studies discussed the effect of exercise on the overall health of the elderly and further discussed the impact on cognitive function. There were also some studies on intervention and general health in specific groups (e.g., elderly people in prison, veterans, women during menopause, elderly volunteers/workers, etc.). This classification involves a variety of targets and is comprehensive, and is therefore likely to be one of the research focuses of health promotion in the elderly in the future.

The fourth largest category was quality of life, which comprises health status, social contacts, and other elements [10] and is also related to the impact of declines in function [11]. In the screening results for this project, in addition to some of the studies sharing common research themes with other interventions such as physical activity, mental health, psychosocial functioning, sleep quality, etc., there were also studies focusing on independent topics. For example, there were many papers on prevention of abuse and neglect of older adults, and some other papers on the prevention of suicidal behavior and reduction in suicidal ideation, reminiscence therapy interventions, environmental and behavioral modifications, preventive house visits, lifelong learning, etc. The research themes in the relevant studies were quite diverse and will contribute to the formulation of future health and social services policies for the elderly [10].

The fifth largest category in the purpose-oriented categories was cognitive function, which was compared to “frailty” based on Duplaga’s screening results (2016), which was the only difference among the top five indices. According to the current study, cognitive decline is one of the symptoms of dementia and is not directly related to aging, physical health status, and pre-morbid activity patterns [12]. This discourse fits the results of the present screening. There was an overwhelming majority of the studies related to dementia. The results of the screening also reveal a wide range of interventions, including non-pharmacological interventions such as physical activity, structural brain plasticity-induced training, video games, electronic-assistive devices, etc. In the face of dementia, which has a longer course of disease and high incidence rates [13], it is necessary to specify multiple interventions as a research topic to find more effective treatments intended to slow cognitive decline.

In addition to focusing on the distribution of the studies under consideration in this study, studies on the application of eHealth technology were also examined. According to the findings, eHealth technology has been used in recent years as an intervention tool for health promotion. These tools include virtual reality (*n* = 3), smart homes and home health monitoring technologies (*n* = 1), socially assistive robots (*n* = 5), and electronic assistive technology (*n* = 3).

Some studies explored whether the use of virtual reality technology can improve problems that lead to debilitation in the elderly and prevent them from falling. The results show that virtual reality games are better than traditional interventions for improving balance and preventing falls in the elderly and can strengthen the awareness of the harm caused by falls in the elderly [14]. Scoglio and Abdi et al. investigated whether socially assistive robots can help the mental health and well-being of the elderly, and their findings suggested that there is positive potential for companionship, cognition, and mental health improvements associated with their use. However, due to the limited number of people involved in the related research and some methodological problems, the helpfulness and value of socially assistive robots in the health promotion of elderly people still requires further research. [15,16]. Elderly people have both positive and negative views of socially assistive robots in geriatric care. A further understanding of experience with and willingness toward use is needed to consider the use of socially assistive robots in this area [17]. Brims and Oliver suggested that the effectiveness of assistive technology in reducing hospital admission rates is inconclusive. However, assistive technology has been tested to reduce the risk of falls, accidents, and other risky behavior, as well as to improve home safety for people with dementia [18]. Nevertheless, Roest’s study indicated that it is not clear whether AT can actually help resolve dementia-related memory problems [19]. Tangcharoensathien’s research pointed out that the promotion of assistive technologies requires the establishment of a policy framework and the encouragement of assistive technology product development through the training of professionals and support and promotion programs [20]. Liu et al. reviewed the impact of smart homes and health monitoring technologies on the health of the elderly, and their results show that eHealth technology could be used to monitor daily life, cognitive decline, mental health, and heart conditions in elderly individuals with complex needs. However, smart homes and home health monitoring technologies are not yet mature [21].

The authors found that most of the studies on eHealth technology interventions were focused on treatment rather than prevention. Seven out of 12 studies using eHealth technology focused more on treatment. For example, Roest et al. [19] focused on how to make use of assistive technology (AT) to help dementia patients and their caregivers manage their daily lives and enhance their safety [17]. Abdi et al. [15] suggested five roles for social assistive technology (SAR), which included affective therapy, cognitive training, social facilitation, companionship, and physiological therapy. Four of the studies focused more on prevention. Neri et al. studied improvements in balance and mobility in the elderly [17]. Liu et al. investigated the interaction between smart homes and home health monitoring technologies and life functioning, cognitive ability, mental health, and cardiac status among older adults [17]. There was also one study that focused on both treatment and prevention, Afsaneh et al. [22] found that self-care ATs can efficiently reduce care hours and also help to increase levels of independence. In this study, only 2.5% (*n* = 12) of the studies were on the use of eHealth technologies among the searchable reviews. However, with the rapid development of technology (e.g., where robots may gradually replace people, and 5G technology will be mature), older people are becoming increasingly more receptive to technology. It is expected that the use of eHealth technology as an intervention tool for health promotion in the elderly will become a trend, and in the future, more in-depth research can be conducted on the use of eHealth technology as an intervention tool for health promotion in the elderly. Therefore, before using eHealth technology in this way, it is important to know how receptive older people are to eHealth technology in order to develop strategies for its use among different groups, such as women, aborigines, and people with dementia. In this study, the results of these studies also show that eHealth technology as a means of health promotion for the elderly is not yet mature and requires larger samples and more rigorous research designs.

The present study has the following limitations: First, there were different research standards for each paper. Through the comparison, it was found that in fact, there are different types of studies, such as meta-analyses and systematic reviews, different types of participants, including different health conditions and ages, different types of interventions, and various outcome measures and analysis technologies used for research statistics and analysis purposes. This may have led to errors in the literature comparison. Secondly, not all of the studies under review had specific results due to such things as time limitation, outcomes being both positive and negative, and a lack of maturity of the technologies leading to adequate experience and data to analyze, etc. Muellmann et al.’s study noted that due to a lack of research time, it was impossible to confirm whether long-term health interventions increase physical activity in 55 year-old individuals [17]. Vandemeulebroucke’s research mentioned that there were both positive and negative results found for the use of socially assistive robots in the area of elderly care. They demonstrated that technology intervention in the area of elderly care is not yet mature, and it is necessary to learn more about the past life experiences of the elderly and to use more rigorous research designs in more groups [17]. In addition, some studies mentioned that before using technology as an intervention for health promotion in the elderly, it is important to determine how receptive older people are to this technology in order to develop strategies suitable for different groups, such as women, aborigines, and people with dementia. It was also mentioned in many studies that more study is needed for corroboration due to the shortage of existing research on this topic. Therefore, there is also no definitive conclusion as to whether technology promotes behavioral change [23]. Another limitation is language restrictions. This study includes reviews written in English. Further studies are suggested to include works written in other languages.

It was found in this study that the presence of diseases and co-morbidities is an important direction of concern for health promotion in the elderly. Health promotion in the elderly typically has three purposes: maintaining and increasing function, maintaining or improving self-managed health, and creating active social networks. However, all of these aims are intended to contribute to independence and a better quality of life. Older people struggle to stay healthy, not only because of the challenges of aging, but also because they are more likely to be ostracized and socially isolated than younger people. However, subjective or psychosocial factors are often neglected in the implementation or formulation of advanced age health promotion programs. Therefore, it is important to go beyond the physical to further explore the psychosocial components of a strategy and how these factors affect overall happiness. Self-reported survey data can be used to measure psychosocial factors (e.g., loneliness, self-efficacy) so as to provide a clearer picture of the real needs of this population.

## Figures and Tables

**Table 1:** Keywords used in the search related to health promotion.

Population(P): Elderly	Concept(C): Health Promotion	Context(C): Effectiveness
Ag(e)ing Aged Advanced aged Elderly Elder/Elders Geriatric Old age Old people Senior(s) Sensorial Senior citizen	Addiction Behavior modification Campaign(s) Community mobilization Environment change Habits Healthy environment Health program Health program Health education Health literacy Health communication Health advocacy Health coaching Health changes Health screening Intervention Nutrition Preventive/Prevention interventions Primary prevention Prophylaxis Physical activity Screening Social careSocial support social intervention Social campaign(s) Strategies Support groups Social network Social gathering	Efficiency Evidence Efficacy Effectiveness Impact Outcomes
	Systematic review Meta analysis/Metanalysis/Meta-analysis	

## Supplementary Materials

The following are available online at https://www.mdpi.com/1660-4601/17/15/5335/s1. Supplementary 1: Definitions used in the process of classification, Supplementary 2: Preferred Reporting Items for Systematic reviews and Meta-Analyses extension for Scoping Reviews (PRISMA-ScR) Checklist, Supplementary 3: List of systematic reviews and meta-analyses included in this study. Supplementary 4: Comparison between the current study and Duplaga et al. [4]
