# Supplementary material for: Health Promotion and Disease Prevention Interventions for the Elderly: A Scoping Review from 2015–2019
Source: Int J Environ Res Public Health. 2020 Jul 24;17(15):5335. doi: 10.3390/ijerph17155335 (PMC7432678; doi:10.3390/ijerph17155335)
Supplement: Supplementary file 1 [file ijerph-17-05335-s001.pdf]

Supplementary 1

1. Health promotion

the process of enabling people to increase control over their health and its determinants, and thereby improve their health. 〔1〕

2. Primary prevention

Primary prevention refers to actions aimed at avoiding the manifestation of a disease. 〔2〕

3. Screening

a preliminary procedure, such as a test or examination, to detect the most characteristic sign or signs of a disorder that may require further investigation. 〔3〕

4. Social support

Social support refers to a person’ s perception of the availability of help or support from others in their social network〔4〕

5. E Health

eHealth is the cost-efective and secure use of ICT in support of health and health-related felds, including health-care services, health surveillance, health literature, and health education, knowledge and research. 〔5〕〔6〕

References

〔1〕World Health Organization (WHO). The Bangkok Charter for Health Promotion in a Globalized World, Global Conference on Health Promotion, Bangkok, Thailand, August 2005

〔2〕Gullotta, T. P., & Bloom, M. (Eds.). (2003). Encyclopedia of primary prevention and health promotion. Springer Science & Business Media.

〔3〕O'Toole, Marie T., ed. (2013). Mosby's medical dictionary (9th ed.). St. Louis, Mo. Elsevier/Mosby. Kindle loc. 145535

〔4〕Berkman LF, Glass T, Brissette I, Seeman TE. From social integration to health: Durkheim in the new millennium. Soc Sci Med. 2000;51(6):843–57.

〔5〕World Health Organization (WHO). WHA58. 28 e-Health. Geneva: WHO.

〔6〕Blobel, B., Pharow, P., & Nerlich, M. (Eds.). (2008). eHealth: Combining health telematics, telemedicine, biomedical engineering, and bioinformatics to the edge: global experts summit textbook (Vol. 134). Ios Press.

Supplementary 2

Preferred Reporting Items for Systematic reviews and Meta-Analyses extension for Scoping Reviews (PRISMA-ScR) Checklist

SECTION	ITEM	PRISMA-ScR CHECKLIST ITEM	REPORTED ON PAGE #
**TITLE**			
Title	1	Identify the report as a scoping review.	1
**ABSTRACT**			
Structured summary	2	Provide a structured summary that includes (as applicable): background, objectives, eligibility criteria, sources of evidence, charting methods, results, and conclusions that relate to the review questions and objectives.	1
**INTRODUCTION**			
Rationale	3	Describe the rationale for the review in the context of what is already known. Explain why the review questions/objectives lend themselves to a scoping review approach.	2
Objectives	4	Provide an explicit statement of the questions and objectives being addressed with reference to their key elements (e.g., population or participants, concepts, and context) or other relevant key elements used to conceptualize the review questions and/or objectives.	2<br>Supplementary 1
**METHODS**			
Protocol and registration	5	Indicate whether a review protocol exists; state if and where it can be accessed (e.g., a Web address); and if available, provide registration information, including the registration number.	x
Eligibility criteria	6	Specify characteristics of the sources of evidence used as eligibility criteria (e.g., years considered, language, and publication status), and provide a rationale.	3-4
Information sources*	7	Describe all information sources in the search (e.g., databases with dates of coverage and contact with authors to identify additional sources), as well as the date the most recent search was executed.	5
Search	8	Present the full electronic search strategy for at least 1 database, including any limits used, such that it could be	4-5

SECTION	ITEM	PRISMA-ScR CHECKLIST ITEM	REPORTED ON PAGE #
		repeated.	
Selection of sources of evidence†	9	State the process for selecting sources of evidence (i.e., screening and eligibility) included in the scoping review.	3
Data charting process‡	10	Describe the methods of charting data from the included sources of evidence (e.g., calibrated forms or forms that have been tested by the team before their use, and whether data charting was done independently or in duplicate) and any processes for obtaining and confirming data from investigators.	4-5
Data items	11	List and define all variables for which data were sought and any assumptions and simplifications made.	Not applicable
Critical appraisal of individual sources of evidence§	12	If done, provide a rationale for conducting a critical appraisal of included sources of evidence; describe the methods used and how this information was used in any data synthesis (if appropriate).	Not applicable
Synthesis of results	13	Describe the methods of handling and summarizing the data that were charted.	4-5
**RESULTS**			
Selection of sources of evidence	14	Give numbers of sources of evidence screened, assessed for eligibility, and included in the review, with reasons for exclusions at each stage, ideally using a flow diagram.	6
Characteristics of sources of evidence	15	For each source of evidence, present characteristics for which data were charted and provide the citations.	6-9
Critical appraisal within sources of evidence	16	If done, present data on critical appraisal of included sources of evidence (see item 12).	Not applicable
Results of individual sources of evidence	17	For each included source of evidence, present the relevant data that were charted that relate to the review questions and objectives.	Supplementry 4
Synthesis of results	18	Summarize and/or present the charting results as they relate to the review questions and objectives.	6-8

SECTION	ITEM	PRISMA-ScR CHECKLIST ITEM	REPORTED ON PAGE #
**DISCUSSION**			
Summary of evidence	19	Summarize the main results (including an overview of concepts, themes, and types of evidence available), link to the review questions and objectives, and consider the relevance to key groups.	8-10
Limitations	20	Discuss the limitations of the scoping review process.	11-12
Conclusions	21	Provide a general interpretation of the results with respect to the review questions and objectives, as well as potential implications and/or next steps.	12
**FUNDING**			
Funding	22	Describe sources of funding for the included sources of evidence, as well as sources of funding for the scoping review. Describe the role of the funders of the scoping review.	12

JBI = Joanna Briggs Institute; PRISMA-ScR = Preferred Reporting Items for Systematic reviews and Meta-Analyses extension for Scoping Reviews.

* Where *sources of evidence* (see second footnote) are compiled from, such as bibliographic databases, social media platforms, and Web sites.

† A more inclusive/heterogeneous term used to account for the different types of evidence or data sources (e.g., quantitative and/or qualitative research, expert opinion, and policy documents) that may be eligible in a scoping review as opposed to only studies. This is not to be confused with *information sources* (see first footnote).

‡ The frameworks by Arksey and O'Malley (6) and Levac and colleagues (7) and the JBI guidance (4, 5) refer to the process of data extraction in a scoping review as data charting.

§ The process of systematically examining research evidence to assess its validity, results, and relevance before using it to inform a decision. This term is used for items 12 and 19 instead of "risk of bias" (which is more applicable to systematic reviews of interventions) to include and acknowledge the various sources of evidence that may be used in a scoping review (e.g., quantitative and/or qualitative research, expert opinion, and policy document).

From: Tricco AC, Lillie E, Zarin W, O'Brien KK, Colquhoun H, Levac D, et al. PRISMA Extension for Scoping Reviews (PRISMAScR): Checklist and Explanation. Ann Intern Med. 2018;169:467–473. doi: 10.7326/M18-0850.

St. Michael's
Inspired Care.
Inspiring Science.

Supplementary 3

Title	Year of publication	Population, gender	General area of intervention	Targeted area
A Bespoke Kinect Stepping Exergame for Improving Physical and Cognitive Function in Older People: A Pilot Study.[1]	2016	70+, both gender	health promotion & primary prevention	physical activity, frailty
A critical appraisal of nutritional intervention studies in malnourished, community dwelling older persons.[2]	2016	general elderly, both gender	health promotion & screening	nutrition
A Meta-Analysis of the Correlates of Successful Aging in Older Adults[3]	2017	general elderly, both gender	screening	general health
A meta-analytic review on social relationships and suicidal ideation among older adults.[4]	2017	general elderly, both gender	screening & social support	psychosocial functioning, quality of life
A realist review to understand the efficacy and outcomes of interventions designed to minimise, reverse or prevent the progression of frailty.[5]	2018	general elderly, both gender	health promotion & primary prevention & screening	general health, frailty
A shared mealtime approach to improving social and nutritional functioning among older adults living alone: study protocol for a randomized controlled trial.[6]	2015	general elderly, both gender	health promotion & social support	nutrition,independence
A systematic literature review and meta-analysis of real-world interventions for cognitive ageing in healthy older adults.[7]	2019	general elderly, both gender	ALL	physical activity,
A systematic review and meta-analysis of interventions designed to prevent or stop elder maltreatment.[8]	2016	general elderly, both gender	primary prevention & screening	quality of life
A systematic review and meta-analysis of outcome measures to assess postural control in older adults who undertake exergaming[9]	2017	60+, both gender	health promotion & screening	physical activity

A systematic review and meta-analysis of structural magnetic resonance imaging studies investigating cognitive and social activity levels in older adults.[10]	2018	general elderly, both gender	primary prevention	psychosocial functioning,
A systematic review and meta-analysis of the criterion validity of nutrition assessment tools for diagnosing protein-energy malnutrition in the older community setting (the MACRo study).[11]	2018	65+, both gender	primary prevention & screening	nutrition
A systematic review identifying the impact of patient decision aids in older patients with problematic polypharmacy.[12]	2019	65+, both gender	primary prevention & screening & social support	disease oriented
A systematic review of correlates of depression among older Chinese and Korean immigrants: what we know and do not know.[13]	2018	general elderly, both gender	screening & social support	mental health,
A systematic review of economic evaluations of seasonal influenza vaccination for the elderly population in the European Union.[14]	2017	general elderly, both gender	primary prevention & screening	disease oriented
A systematic review of intervention approaches for driving cessation in older adults.[15]	2017	general elderly, both gender	screening	addicition
A systematic review of interventions for older adults living in jails and prisons.[16]	2019	general elderly, both gender	ALL	general health,
A systematic review of interventions to prevent suicidal behaviors and reduce suicidal ideation in older people.[17]	2017	general elderly, both gender	primary prevention & screening	mental health,
A systematic review of non-pharmacological interventions for BPSD in nursing home residents with dementia: from a perspective of ergonomics.[18]	2019	general elderly, both gender	health promotion & screening	disease oriented
A Systematic Review of Reminiscence Therapy for Older Adults in Taiwan.[19]	2018	general elderly, both gender	health promotion & screening & social support	mental health,disease oriented, cognitive function

A systematic review of research into how robotic technology can help older people.[20]	2018	general elderly, both gender	health promotion & screening & social support	independence,cognitive function
A systematic review of self-care assistive technologies for aging population.[21]	2018	general elderly, both gender	health promotion & screening & social support	quality of life,
A Systematic Review of the Measurement of Function in Late-Life Depression.[22]	2018	60+, both gender	health promotion & screening	mental health,disease oriented
A systematic review of the prevalence of comorbid cancer and dementia and its implications for cancer-related care.[23]	2018	general elderly, both gender	health promotion	disease oriented
A systematic review on community-based interventions for elder abuse and neglect.[24]	2017	general elderly, both gender	health promotion & primary prevention	quality of life
A systematic review and meta-analysis of exercise-based falls prevention strategies in adults aged 50+ years with visual impairment.[25]	2018	50+, both gender	health promotion	independence,
A systematic review of interventions to promote work participation in older workers.[26]	2017	general elderly, both gender	health promotion	general health
A systematic review of social support interventions for caregivers of people with dementia: Are they doing what they promise?[27]	2016	general elderly, both gender	health promotion & social support	disease oriented,
A Systematic Review on the Cognitive Benefits and Neurophysiological Correlates of Exergaming in Healthy Older Adults.[28]	2019	60+, both gender	health promotion & primary prevention	cognitive function,
Acupoint Massage for Managing Cognitive Alterations in Older Adults: A Systematic Review and Meta-Analysis.[29]	2018	60+, both gender	health promotion	cognitive function
Acutherapy for Knee Osteoarthritis Relief in the Elderly: A Systematic Review and Meta-Analysis.[30]	2019	general elderly, both gender	health promotion	disease oriented

Addressing social isolation to improve the health of older adults: A rapid evidence review.[31]	2019	60+, both gender	health promotion & screening & social support	quality of life,
Adherence to community based group exercise interventions for older people: A mixed-methods systematic review.[32]	2016	general elderly, both gender	health promotion & screening	physical activity,
Adherence to the WCRF/AICR dietary recommendations for cancer prevention and risk of cancer in elderly from Europe and the United States: A meta-Analysis within the CHANCES Project.[33]	2017	60+, both gender	health promotion & primary prevention	disease oriented,
Aerobic exercise ameliorates cognitive function in older adults with mild cognitive impairment: a systematic review and meta-analysis of randomised controlled trials.[34]	2016	general elderly, both gender	health promotion	cognitive function,
Aerobic exercise effects upon cognition in Alzheimer's Disease: A systematic review of randomized controlled trials.[35]	2018	general elderly, both gender	health promotion	cognitive function,disease oriented
Age at menopause and duration of reproductive period in association with dementia and cognitive function: A systematic review and meta-analysis.[36]	2016	general elderly，女	screening	frailty,
Ageing and Learning: What Do They Mean to Elders Themselves?[37]	2016	55-75, both gender	health promotion & screening	general health,
Ageing, Muscle Power and Physical Function: A Systematic Review and Implications for Pragmatic Training Interventions.[38]	2016	general elderly, both gender	health promotion & screening	frailty,
Aging and Functional Health Literacy: A Systematic Review and Meta-Analysis.[39]	2015	50+, both gender	screening	frailty
Aging, Precarity, and the Struggle for Indigenous Elsewheres.[40]	2018	65+, both gender	screening	psychosocial functioning,
AGITE-a medication self-management and adherence in the elderly questionnaire.[41]	2017	64+, both gender	screening	disease oriented

Alcohol Use Disorder in Older Adults: Challenges in Assessment and Treatment.[42]	2017	general elderly, both gender	health promotion & screening & social support	quality of life,
An Architecture to Support Wearables in Education and Wellbeing.[43]	2017	general population, both gender	primary prevention & screening	mental health,quality of life
An economic case for the surgical treatment of type-II odontoid fractures in the elderly: A markov cost-utility analysis based on the prospective aospine geriatric odontoid fracture study.[44]	2017	general elderly, both gender	health promotion	disease oriented
Anorexia in older people and its treatment: A systematic review.[45]	2019	65+, both gender	health promotion	nutrition,
Antidepressants for anxiety disorders in late-life: A systematic review.[46]	2019	general elderly, both gender	health promotion & screening	disease oriented
Antipsychotics, antidepressants, anticonvulsants, melatonin, and benzodiazepines for behavioral and psychological symptoms of dementia: A systematic review of meta-analyses.[47]	2018	general elderly, both gender	screening	mental health,
Are falls prevention programs effective at reducing the risk factors for falls in people with type-2 diabetes mellitus and peripheral neuropathy: A systematic review with narrative synthesis.[48]	2017	general elderly, both gender	primary prevention & screening	frailty,
Are interventions effective in improving the ability of older adults to rise from the floor independently? A mixed method systematic review.[49]	2018	60+, both gender	health promotion	frailty,
Assessing prevention strategies for Alzheimer's dementia: A new approach.[50]	2017	general elderly, both gender	health promotion & primary prevention	disease oriented
Assessment and Treatment of the Anorexia of Aging: A Systematic Review.[51]	2019	65+, both gender	health promotion	nutrition,

Assessment of Questionnaire Measuring Quality of Life in Menopausal Women: A Systematic Review.[52]	2015	general elderly, female	screening	quality of life
Assistance at mealtimes in hospital settings and rehabilitation units for patients (>65years) from the perspective of patients, families and healthcare professionals: A mixed methods systematic review.[53]	2017	65+, both gender	health promotion & social support	disease oriented,
Assistive technology for memory support in dementia.[54]	2017	general elderly, both gender	ALL	disease oriented,cognitive function
Association Between Cerebral Microbleeds and Depression in the General Elderly Population: A Meta-Analysis.[55]	2018	general elderly, both gender	health promotion & screening	mental health,
Association between frailty and quality of life among community-dwelling older people: a systematic review and meta-analysis.[56]	2016	general elderly, both gender	health promotion & screening	frailty,
Association Between Hormone Therapy and Muscle Mass in Postmenopausal Women: A Systematic Review and Meta-analysis.[57]	2019	50+, female	health promotion	frailty,
Association Between Non-Steroidal Anti-Inflammatory Drug Use and Cognitive Decline: A Systematic Review and Meta-Analysis of Prospective Cohort Studies.[58]	2016	general elderly, both gender	screening	cognitive function
Association Between Psychological Interventions and Chronic Pain Outcomes in Older Adults: A Systematic Review and Meta-analysis. [59]	2018	60+, both gender	health promotion	mental health,
Association between sarcopenia and osteoarthritis-related knee structural changes: a systematic review.[60]	2019	general elderly, both gender	health promotion	frailty,
Association between stroke and Alzheimer's disease: Systematic review and Meta-analysis.[61]	2015	general elderly, both gender	health promotion & screening	disease oriented

Association of a priori dietary patterns with depressive symptoms: A harmonised meta-analysis of observational studies.[62]	2019	general population, both gender	screening	nutrition,mental health
Associations between Sarcopenia and Metabolic Risk Factors: A Systematic Review and Meta-Analysis.[63]	2019	general elderly, both gender	health promotion & screening	general health,
Auditory deprivation and health in the elderly.[64]	2016	general elderly, both gender	health promotion	general health,quality of life
Barriers and facilitators to adherence to group exercise in institutionalized older people living with dementia: a systematic review.[65]	2018	general elderly, both gender	health promotion & social support	physical activity,
Behavioral Activation Therapy for Older Adults with Depression: A Systematic Review of Effectiveness.[66]	2018	50+, both gender	health promotion & screening & social support	mental health,general health, psychosocial functioning
Behavioral Interventions in Six Dimensions of Wellness That Protect the Cognitive Health of Community-Dwelling Older Adults: A Systematic Review.[67]	2016	60+, both gender	health promotion & screening	general health,
Benefits and Dynamics of Learning Gained through Volunteering: A Qualitative Exploration Guided by Seniors' Self-Defined Successful Aging.[68]	2016	60+, both gender	social support & screening	psychosocial functioning,
Benefits and Harms of Treating Blood Pressure in Older Adults: A Systematic Review and Meta-analysis.[69]	2016	general elderly, both gender	health promotion & screening	cognitive function,
Best-Practice Guideline on the Prevention of Abuse and Neglect of Older Adults.[70]	2016	general elderly, both gender	health promotion & screening	quality of life
Built environmental correlates of older adults' total physical activity and walking: a systematic review and meta-analysis.[71]	2017	65+, both gender	health promotion & screening	quality of life,

Can physical activity be used to maintain cognitive function in nursing home residents with dementia? A literature review[72]	2019	general elderly, both gender	health promotion & screening	physical activity,cognitive function
Can we reduce falls and fractures?[73]	2017	general elderly, both gender	primary prevention	frailty
Cause or Effect? Selective Serotonin Reuptake Inhibitors and Falls in Older Adults: A Systematic Review.[74]	2015	general elderly, both gender	screening	frailty
Center of pressure characteristics from quiet standing measures to predict the risk of falling in older adults: a protocol for a systematic review and meta-analysis.[75]	2019	60+, both gender	health promotion & primary prevention & screening	frailty
Cerebral changes improved by physical activity during cognitive decline: A systematic review on MRI studies.[76]	2019	general elderly, both gender	health promotion & primary prevention	cognitive function,
Challenges for the Healthy Ageing in People with Intellectual Disabilities.[77]	2019	60+, both gender	screening	disease oriented,disability
Changes in Sleep Predict Changes in Affect in Older Caregivers of Individuals with Alzheimer's Dementia: A Multilevel Model Approach.[78]	2016	50+, both gender	screening	sleep quality,
Characteristics and effectiveness of fall prevention programs in nursing homes: a systematic review and meta-analysis of randomized controlled trials.[79]	2015	general elderly, both gender	health promotion & primary prevention	frailty
Characteristics of Older Adults in Medication-Assisted Treatment for Opioid Use Disorder: A Systematic Review.[80]	2019	general elderly, both gender	health promotion & screening	frailty,
Clinical medication review as part of an elderly fallers patient care screening service.[81]	2015	general elderly, both gender	health promotion & screening	frailty

Cognitive and gait decrements among non-demented older adults with Type 2 diabetes or hypertension: a systematic review.[82]	2018	general elderly, both gender	screening	disease oriented,frailty
Cognitive and neural plasticity in old age: A systematic review of evidence from executive functions cognitive training.[83]	2019	general elderly, both gender	health promotion & primary prevention	cognitive function
Cognitive effects of video games in older adults and their moderators: a systematic review with meta-analysis and meta-regression.[84]	2019	general elderly, both gender	health promotion	cognitive function
Cognitive Function and Calcium. Hypertension treatment in consideration of dementia.[85]	2015	general elderly, both gender	health promotion	cognitive function,
Cognitive Interventions for Cognitively Healthy, Mildly Impaired, and Mixed Samples of Older Adults: A Systematic Review and Meta-Analysis of Randomized-Controlled Trials.[86]	2017	60+, both gender	health promotion & screening	cognitive function,
Cognitive leisure activities and future risk of cognitive impairment and dementia: systematic review and meta-analysis.[87]	2016	general elderly, both gender	health promotion & primary prevention & screening	cognitive function,disease oriented
Cognitive Training Interventions for Patients with Alzheimer's Disease: A Systematic Review.[88]	2017	general elderly, both gender	health promotion	disease oriented,
Collaborative Care for Psychiatric Disorders in Older Adults: A Systematic Review.[89]	2017	60+, both gender	health promotion	disease oriented,
Combining music and reminiscence therapy interventions for wellbeing in elderly populations: A systematic review.[90]	2017	general elderly, both gender	health promotion & social support	general health,
Community screening for visual impairment in older people.[91]	2018	65+, both gender	screening	disease oriented
Comparative Effectiveness of Published Interventions for Elderly Fall Prevention: A Systematic Review and Network Meta-Analysis.[92]	2018	general elderly, both gender	health promotion & screening	physical activity,general health

Comparison of diagnostic performance of Two-Question Screen and 15 depression screening instruments for older adults: systematic review and meta-analysis[93]	2017	general elderly, both gender	screening	mental health,
Comparisons of Interventions for Preventing Falls in Older Adults: A Systematic Review and Meta-analysis.[94]	2017	65+, both gender	primary prevention & screening	frailty
Comprehensive geriatric assessment for older adults admitted to hospital.[95]	2017	general elderly, both gender	screening	general health
Computer-based cognitive interventions for people living with dementia: a systematic literature review and meta-analysis.[96]	2017	general elderly, both gender	screening	cognitive function
Consequences of Elder Abuse and Neglect: A Systematic Review of Observational Studies.[97]	2018	general elderly, both gender	screening	quality of life
Constipation is casting a shadow over everyday life - a systematic review on older people's experience of living with constipation.[98]	2016	general elderly, both gender	health promotion & screening	general health,
Contemporary views on dementia as witchcraft in sub-Saharan Africa: A systematic literature review.[99]	2019	general elderly, both gender	health promotion	psychosocial functioning,
Continuation and maintenance treatments for depression in older people.[100]	2016	60+, both gender	health promotion & screening	disease oriented,
Contribution of NO and impact of aging in flow mediated dilatation in healthy patients: Meta-analysis and meta-regression.[101]	2018	general elderly, both gender	screening	frailty,
Controversies about insomnia therapy in elderly.[102]	2016	general elderly, both gender	screening	sleep quality
Cost-effectiveness analysis of universal vaccination of adults aged 60 years with 23-valent pneumococcal polysaccharide vaccine versus current practice in Brazil.[103]	2015	60+, both gender	health promotion & primary prevention	disease oriented

Cost-effectiveness of the treatment of depression in old age: A systematic review.[104]	2015	general elderly, both gender	social support & screening	mental health,
Dance for People with Alzheimer's Disease: A mini-Review.[105]	2019	general elderly, both gender	health promotion & screening	quality of life,physical activity
Defining quality of life measures in elderly populations with dementia: A Systematic Review.[106]	2019	65+, both gender	screening	quality of life,
Delaying and reversing frailty: A systematic review of primary care interventions.[107]	2018	general elderly, both gender	primary prevention & screening	physical activity,nutrition
Dementia and co-occurring chronic conditions: a systematic literature review to identify what is known and where are the gaps in the evidence?[108]	2017	general elderly, both gender	screening	frailty,cognitive function
Depression and anxiety after total joint replacement among older adults: a meta-analysis.[109]	2016	50+, both gender	health promotion & screening	mental health,
Depression and frailty in later life: A systematic review.[110]	2015	general elderly, both gender	screening	mental health,frailty
Depression and Reduced Bone Mineral Density at the Hip and Lumbar Spine: A Comparative Meta-Analysis of Studies in Adults 60 Years and Older.[111]	2016	60+, both gender	screening	mental health,
Design of controls in trials of computerised cognitive training is ineffectual: A meta-analysis in healthy older adults.[112]	2017	general elderly, both gender	primary prevention & screening	cognitive function
Detection and Prevention of Cognitive Decline.[113]	2016	general elderly, both gender	health promotion & primary prevention & screening	cognitive function

Development and Validation of an Interactive Internet Platform for Older People: The Healthy Ageing Through Internet Counselling in the Elderly Study.[114]	2016	65+, both gender	health promotion	general health
Development of an Instrument for Assessing Elder Care Needs.[115]	2017	general elderly, both gender	social support & screening	psychosocial functioning,
Dexmedetomidine for the prevention of postoperative delirium in elderly patients undergoing noncardiac surgery: A meta-analysis of randomized controlled trials.[116]	2019	general elderly, both gender	primary prevention	disease oriented
Diabetes Treatment in the Elderly: Incorporating Geriatrics, Technology, and Functional Medicine.[117]	2018	general elderly, both gender	health promotion	disease oriented
Diet Quality and Sarcopenia in Older Adults: A Systematic Review.[118]	2018	general elderly, both gender	health promotion	nutrition,
Dietary Patterns and Quality of Life in Older Adults: A Systematic Review.[119]	2018	general elderly, both gender	health promotion	nutrition,
Dietary patterns and successful ageing: a systematic review.[120]	2016	general elderly, both gender	screening	general health,
Digital behavior change interventions to promote physical activity and/or reduce sedentary behavior in older adults: A systematic review and meta-analysis.[121]	2019	50+, both gender	health promotion & screening	physical activity,
Do low-serum vitamin E levels increase the risk of Alzheimer disease in older people? Evidence from a meta-analysis of case-control studies.[122]	2018	60+, both gender	screening	disease oriented

Do virtual reality games improve mobility skills and balance measurements in community-dwelling older adults? Systematic review and meta-analysis.[123]	2017	general elderly, both gender	health promotion	frailty,
Does aquatic exercise improve commonly reported predisposing risk factors to falls within the elderly? A systematic review.[124]	2019	60-90, both gender	health promotion	physical activity
Does Social Support Affect the Health of the Elderly in Rural China? A Meta-Analysis Approach.[125]	2019	general elderly, both gender	social support	general health,
Dog Visiting Programs for Managing Depressive Symptoms in Older Adults: A Meta-analysis.[126]	2018	general elderly, both gender	health promotion & primary prevention	mental health
Drinking in later life: a systematic review and thematic synthesis of qualitative studies exploring older people's perceptions and experiences.[127]	2018	60+, both gender	screening	frailty,
Early Detection of Mild Cognitive Impairment With In-Home Monitoring Sensor Technologies Using Functional Measures: A Systematic Review.[128]	2019	general elderly, both gender	health promotion	disease oriented,cognitive function
Ecological Gait as a Fall Indicator in Older Adults: A Systematic Review.[129]	2019	general elderly, both gender	health promotion & primary prevention	frailty
Economic evaluation of health promotion and primary prevention actions for older people-a systematic review.[130]	2017	general elderly, both gender	health promotion & primary prevention	general health
Economic evaluation of health promotion interventions for older people: do applied economic studies meet the methodological challenges?[131]	2018	general elderly, both gender	health promotion & screening	general health
Economic potential of probiotic supplementation in institutionalized elderly with chronic constipation.[132]	2018	general elderly, both gender	health promotion & screening	quality of life,independence

Effect of a multifactorial, interdisciplinary intervention on falls and fall rate of the older people in the community.[133]	2016	general elderly, both gender	primary prevention & screening	frailty
Effect of advanced age and/or systemic medical conditions on dental implant survival: A systematic review and meta-analysis.[134]	2018	75+, both gender	screening	frailty,
Effect of Apolipoprotein E ε4 Carrier Status on Cognitive Response to Acetylcholinesterase Inhibitors in Patients with Alzheimer's Disease: A Systematic Review and Meta-Analysis.[135]	2018	general elderly, both gender	screening	cognitive function,
Effect of Aromatherapy on the Treatment of Psychological Symptoms in Postmenopausal and Elderly Women: A Systematic Review and Meta-analysis.[136]	2018	general elderly, female	screening	mental health
Effect of creatine supplementation during resistance training on lean tissue mass and muscular strength in older adults: a meta-analysis.[137]	2017	55+, both gender	health promotion & screening	nutrition,
Effect of food plans and training in glycemic and lipid profiles in elderly diabetic type 2: Systematic Review.[138]	2017	general elderly, both gender	health promotion & screening	nutrition,
Effect of Implementing a Discharge Plan on Functional Abilities of Geriatric Patients with Hip Fractures.[139]	2016	60+, both gender	health promotion & screening	disease oriented,
Effect of Interval and Continuous Aerobic Training on Basal Serum and Plasma Brain-Derived Neurotrophic Factor Values in Seniors: A Systematic Review of Intervention Studies.[140]	2017	55+, both gender	health promotion & primary prevention	cognitive function,
Effect of Lavender on Sleep, Sexual Desire, Vasomotor, Psychological and Physical Symptom among Menopausal and Elderly Women: A Systematic Review.[141]	2019	general public, female	screening	sleep quality,mental health, quality of life

Effect of long-term nutraceutical and dietary supplement use on cognition in the elderly: a 10-year systematic review of randomised controlled trials.[142]	2018	65+, both gender	health promotion & screening	cognitive function,
Effect of Milk and Other Dairy Products on the Risk of Frailty, Sarcopenia, and Cognitive Performance Decline in the Elderly: A Systematic Review.[143]	2019	general elderly, both gender	health promotion & primary prevention	disease oriented,cognitive function, frailty
Effect of nonmeat, high-protein supplementation on quality of life and clinical outcomes in older residents of care homes: a systematic review and meta-analysis.[144]	2018	65+, both gender	health promotion	nutrition,
Effect of pilates exercise for improving balance and decreasing falls risk in older adults: A systematic review with meta-analysis.[145]	2015	65+, both gender	health promotion	frailty,
Effect of Tai Chi Exercise on Fall Prevention in Older Adults: Systematic Review and Meta-analysis of Randomized Controlled Trials.[146]	2016	general elderly, both gender	health promotion & primary prevention	physical activity,
Effect of Tai Chi for the prevention or treatment of osteoporosis in elderly adults: protocol for a systematic review and meta-analysis.[147]	2018	general elderly, both gender	health promotion & primary prevention	physical activity,
Effect of vibration on postural control and gait of elderly subjects: a systematic review.[148]	2018	general elderly, both gender	health promotion	quality of life,
Effect of interventions using physical activity trackers on physical activity in people aged 60 years and over: a systematic review and meta-analysis.[149]	2019	60+, both gender	health promotion	general health,
Effect of physical activity and dietary restriction interventions on weight loss and the musculoskeletal function of overweight and obese	2017	55+, both gender	health promotion & screening	disease oriented,nutrition

older adults with knee osteoarthritis: a systematic review and mixed method data synthesis.[150]				
Effectiveness and cost-effectiveness of proactive and multidisciplinary integrated care for older people with complex problems in general practice: an individual participant data meta-analysis.[151]	2018	general elderly, both gender	screening & social support	quality of life,psychosocial functioning
Effectiveness and meaningfulness of art therapy as a tool for healthy aging: A comprehensive systematic review protocol.[152]	2015	general elderly, both gender	health promotion	psychosocial functioning,mental health
Effectiveness of assistive technology in improving the safety of people with dementia: a systematic review and meta-analysis.[153]	2019	general public, both gender	screening	general health,independence, quality of life
Effectiveness of bedrails in preventing falls among hospitalized older adults: A systematic review.[154]	2017	general elderly, both gender	primary prevention	frailty
Effectiveness of eHealth interventions for the promotion of physical activity in older adults: A systematic review.[155]	2018	55+, both gender	health promotion	general health,
Effectiveness of family interventions in nursing homes. A systematic review.[156]	2015	general public, both gender	social support	psychosocial functioning
Effectiveness of Food-Based Fortification in Older People. A Systematic Review and Meta-Analysis.[157]	2016	65+, both gender	health promotion	nutrition
Effectiveness of hospital avoidance interventions among elderly patients: A systematic review.[158]	2018	65+, both gender	screening	quality of life
Effectiveness of interventions aimed at improving physical and psychological outcomes of fall-related injuries in people with dementia: a narrative systematic review.[159]	2018	general elderly, both gender	health promotion	physical activity,disease oriented

Effectiveness of non-pharmacological interventions in treating orthostatic hypotension in the elderly and people with a neurological condition: A systematic review protocol.[160]	2017	general elderly, both gender	health promotion & screening	disease oriented
Effectiveness of nurse-delivered patient education interventions on quality of life in elders in the hospital: A systematic review.[161]	2016	general elderly, both gender	health promotion & social support	quality of life
Effectiveness of nursing discharge planning interventions on health-related outcomes in discharged elderly inpatients: A systematic review.[162]	2016	65+, both gender	health promotion	quality of life,
Effectiveness of nutritional interventions addressed to elderly persons: umbrella systematic review with meta-analysis.[163]	2018	65+, both gender	health promotion & primary prevention	nutrition,
Effectiveness of occupational therapy interventions in acute geriatric wards: A systematic review.[164]	2019	65+, both gender	health promotion	quality of life,
Effectiveness of probiotics in reducing the incidence of Clostridium difficile-associated diarrhea in elderly patients: A systematic review.[165]	2017	60+, both gender	health promotion & screening	nutrition,
Effectiveness of the interventions in preventing the progression of pre-frailty and frailty in older adults: A systematic review protocol.[166]	2016	general elderly, both gender	health promotion & primary prevention	general health,
Effectiveness of training interventions aimed at reducing physical restraints in institutionalised older people: A systematic review.[167]	2017	65+, both gender	health promotion & screening	physical activity,quality of life
Effectiveness of multidisciplinary nutritional support in older hospitalised patients: A systematic review and meta-analyses.[168]	2018	65+, both gender	health promotion	quality of life & nutrition
Effectiveness of peers in delivering programs or motivating older people to increase their participation in physical activity: Systematic review and meta-analysis.[169]	2017	general elderly, both gender	health promotion & social support	psychosocial functioning,

Effectiveness of rehabilitation interventions in improving emotional and functional status in hearing or visually impaired older adults: a systematic review with meta-analyses.[170]	2015	55+, both gender	health promotion	mental health,psychosocial functioning, quality of life
Effectiveness of preventive house visits for elderly people : Systematic assessment of current literature.[171]	2017	60+, both gender	health promotion & social support	quality of life
Effects of dance on cognitive function among older adults: a protocol for systematic review and meta-analysis.[172]	2018	general elderly, both gender	health promotion	cognitive function,
Effects of dance intervention on global cognition, executive function and memory of older adults: a meta-analysis and systematic review.[173]	2019	60+, both gender	health promotion	cognitive function
Effects of Different Chair-Based Exercises on Salivary Biomarkers and Functional Autonomy in Institutionalized Older Women.[174]	2019	71-89, female	health promotion & primary prevention	physical activity,frailty, psychosocial functioning
Effects of exercise interventions for specific cognitive domains in old adults with mild cognitive impairment: A protocol of subgroup meta-analysis of randomized controlled trials.[175]	2018	general elderly, both gender	health promotion & primary prevention	cognitive function,
Effects of Exergaming in People with Dementia: Results of a Systematic Literature Review.[176]	2018	general elderly, both gender	health promotion	physical activity,disease oriented
Effects of Meditation and Mind-Body Exercises on Older Adults' Cognitive Performance: A Meta-analysis.[177]	2019	60+, both gender	primary prevention	cognitive function,
Effects of Milk and Dairy Products on the Prevention of Osteoporosis and Osteoporotic Fractures in Europeans and Non-Hispanic Whites from North America: A Systematic Review and Updated Meta-Analysis.[178]	2019	general elderly, both gender	health promotion & primary prevention	disease oriented,

Effects of Milk Proteins Supplementation in Older Adults Undergoing Resistance Training: A Meta-Analysis of Randomized Control Trials.[179]	2018	60+, both gender	health promotion & screening	nutrition,
Effects of Mind-Body Exercises on Cognitive Function in Older Adults: A Meta-Analysis.[180]	2019	general elderly, both gender	health promotion	cognitive function,
Effects of multi-domain interventions in (pre)frail elderly on frailty, functional, and cognitive status: a systematic review.[181]	2017	65+, both gender	health promotion	frailty,cognitive function, general health
Effects of Oral Health Programs on Xerostomia in Community-Dwelling Elderly: A Systematic Review and Meta-Analysis.[182]	2019	general elderly, both gender	health promotion & screening	disease oriented,general health
Effects of Physical Activity Governmental Programs on Health Status in Independent Older Adults: A Systematic Review.[183]	2019	general elderly, both gender	screening	physical activity,general health
Effects of physical activity on the P300 component in elderly people: a systematic review.[184]	2017	general elderly, both gender	health promotion & screening	cognitive function,
Effects of preventive use of compression stockings for elderly with chronic venous insufficiency and swollen legs: a systematic review and meta-analysis.[185]	2019	general elderly, both gender	health promotion	disease oriented
Effects of Protein Supplementation Combined with Exercise Intervention on Frailty Indices, Body Composition, and Physical Function in Frail Older Adults.[186]	2018	general elderly, both gender	primary prevention	nutrition,frailty
Effects of protein supplementation combined with resistance exercise on body composition and physical function in older adults: a systematic review and meta-analysis.[187]	2017	general elderly, both gender	health promotion	nutrition,quality of life
Effects of relaxation interventions on depression and anxiety among older adults: a systematic review.[188]	2015	general elderly, both gender	screening	mental health,

Effects of resistance training concentric velocity on older adults' functional capacity: A systematic review and meta-analysis of randomised trials.[189]	2019	60+, both gender	health promotion & primary prevention	quality of life,
Efficacy of Cognitive Behavioral Therapy for Generalized Anxiety Disorder in Older Adults: Systematic Review, Meta-Analysis, and Meta-Regression.[190]	2016	general elderly, both gender	health promotion	quality of life,cognitive function
Efficacy of group psychotherapy for geriatric depression: A systematic review.[191]	2018	60+, both gender	health promotion & social support	mental health,psychosocial functioning
Efficacy of Vitamin B Supplementation on Cognition in Elderly Patients With Cognitive-Related Diseases.[192]	2017	general elderly, both gender	health promotion & screening	nutrition,disease oriented
Efficacy and tolerability of pharmacological and non-pharmacological interventions in older patients with major depressive disorder: A systematic review, pairwise and network meta-analysis.[193]	2019	65+, both gender	health promotion	mental health
Efficacy of video game-based interventions for active aging. A systematic literature review and meta-analysis.[194]	2018	general elderly, both gender	health promotion	frailty
Egocentric and allocentric spatial reference frames in aging: A systematic review.[195]	2017	general elderly, both gender	health promotion & screening & social support	quality of life,general health
Elder and Caregiver Solutions to Improve Medication Adherence.[196]	2015	60+, both gender	health promotion	disease oriented,
Electroconvulsive treatment for geriatric depression: A systematic review of randomized clinical trials.[197]	2019	general elderly, both gender	screening	disease oriented
Enhancing Cognitive Functioning in Healthly Older Adults: a Systematic Review of the Clinical Significance of Commercially	2017	general elderly, both gender	health promotion	disease oriented,

Available Computerized Cognitive Training in Preventing Cognitive Decline.[198]				
Environmental and behavioural modifications for improving food and fluid intake in people with dementia.[199]	2018	general elderly, both gender	health promotion & screening	quality of life,disease oriented
Evaluating Exercise Prescription and Instructional Methods Used in Tai Chi Studies Aimed at Improving Balance in Older Adults: A Systematic Review.[200]	2017	60+, both gender	health promotion	physical activity
Evaluating the cost of endocrine therapy vs. radiation therapy alone for low risk hormone positive early stage breast cancer in elderly patients.[201]	2019	general elderly, both gender	primary prevention & screening	disease oriented
Evaluating the effectiveness of exercise training on elderly patients who require haemodialysis: study protocol for a systematic review and meta-analysis.[202]	2016	general elderly, both gender	health promotion & screening	disease oriented,general health
Evaluation of a Computer-Tailored Healthy Ageing Intervention to Promote Physical Activity among Single Older Adults with a Chronic Disease.[203]	2018	68+, both gender	health promotion & screening	physical activity,disease oriented
Evidence-based guidelines for fall prevention in Korea.[204]	2017	65+, both gender	primary prevention & screening	disease oriented,
Evidence-based nutritional and pharmacological interventions targeting chronic low-grade inflammation in middle-age and older adults: A systematic review and meta-analysis.[205]	2018	general elderly, both gender	health promotion & screening	disease oriented,
Exercise for cognitive brain health in aging: A systematic review for an evaluation of dose.[206]	2018	general elderly, both gender	health promotion	physical activity,
Exercise to prevent falls in older adults: an updated systematic review and meta-analysis.[207]	2017	general elderly, both gender	health promotion & primary prevention	physical activity,

Exercise Training for Preventing Dementia, Mild Cognitive Impairment, and Clinically Meaningful Cognitive Decline: A Systematic Review and Meta-analysis.[208]	2017	general elderly, both gender	health promotion & primary prevention	physical activity,
Exercise interventions for cognitive function in adults older than 50: a systematic review with meta-analysis.[209]	2017	50+, both gender	health promotion	physical activity,
Exploring assistive technology as a potential beneficial intervention tool for people with alzheimer's disease – a systematic review.[210]	2018	general elderly, both gender	health promotion	disease oriented
Extending international access to doubly-labelled water data in older adults.[211]	2018	65+, both gender	screening	quality of life
Factors associated with fear of falling and associated activity restriction in community-dwelling older adults: a systematic review.[212]	2015	general elderly, female	health promotion	frailty
Factors Influencing Driving Ability and Its Measurements in Older Drivers: A Systematic Review.[213]	2018	general elderly, both gender	health promotion & screening	frailty,
Fall prevention visit in a residency primary care clinic.[214]	2019	65+, both gender	primary prevention	frailty
Falls in the elderly and senior age prevention perspectives.[215]	2019	55+, both gender	primary prevention	frailty
Feeling younger and identifying with older adults: Testing two routes to maintaining well-being in the face of age discrimination.[216]	2017	50-75, both gender	screening	psychosocial functioning
Fish intake, alzheimer disease genes, and cognitive decline in five cohorts of older subjects.[217]	2015	general elderly, both gender	health promotion & screening	cognitive function,disease oriented
Folic acid supplementation with or without vitamin B12 to prevent cognitive decline in elderly subjects: Critical analysis of literature.[218]	2015	general elderly, both gender	screening	cognitive function,
Formulation of evidence-based messages to promote the use of physical activity to prevent and manage Alzheimer's disease.[219]	2017	general elderly, both gender	health promotion & primary prevention	disease oriented,

Fostering a New Model of Multigenerational Learning: Older Adult Perspectives, Community Partners, and Higher Education.[220]	2016	55+, both gender	social support & screening	independence,psychosocial functioning, general health
Frailty as a Risk Factor for Falls Among Community Dwelling People: Evidence From a Meta-Analysis.[221]	2017	65+, both gender	health promotion & screening	frailty
Functional and Cognitive Impairment, Frailty, and Adverse Health Outcomes in Older Patients Reaching ESRD-A Systematic Review.[222]	2016	60+, both gender	screening	frailty,disability
Functional brain changes associated with cognitive training in healthy older adults: A preliminary ALE meta-analysis.[223]	2019	general elderly, both gender	screening	cognitive function
Functional, nutritional and social factors associated with mobility limitations in the elderly: a systematic review.[224]	2019	general elderly, both gender	health promotion & social support	psychosocial functioning,nutrition
General practice based psychosocial interventions for supporting carers of people with dementia or stroke: a systematic review.[225]	2016	general elderly, both gender	health promotion & social support	psychosocial functioning,mental health
Health and prolonging working lives: an advisory report of the Health Council of The Netherlands.[226]	2019	general elderly, both gender	health promotion	general health
Health benefits of aerobic training programs in adults aged 70 and over: a systematic review.[227]	2017	70+, both gender	health promotion	physical activity
Health benefits of multicomponent training programmes in seniors: a systematic review.[228]	2016	65+, both gender	health promotion	quality of life,
Health benefits of traditional Chinese sports and physical activity for older adults: A systematic review of evidence.[229]	2016	50+, both gender	health promotion	general health,
Health Benefits of β-Hydroxy-β-Methylbutyrate (HMB) Supplementation in Addition to Physical Exercise in Older Adults: A Systematic Review with Meta-Analysis.[230]	2019	50+, both gender	health promotion	nutrition,

Health Effects of the Relocation of Patients With Dementia: A Scoping Review to Inform Medical and Policy Decision-Making.[231]	2018	general elderly, both gender	health promotion	general health
Healthy aging and physical activity: A systematic review on the effects of exercise on cardiovascular diseases.[232]	2017	general elderly, both gender	health promotion & primary prevention & screening	disease oriented,general health
Healthy cognitive aging and dementia prevention.[233]	2016	general elderly, both gender	health promotion & primary prevention	cognitive function,
Healthy Aging in Older Women Living with HIV Infection: a Systematic Review of Psychosocial Factors.[234]	2017	50+, female	health promotion & social support	psychosocial functioning,general health
Heart rate variability alterations in late life depression: A meta-analysis.[235]	2018	60+, both gender	primary prevention & screening	mental health,general health
Help-Seeking Behavior in Victims of Elder Abuse: A Systematic Review.[236]	2019	general elderly, both gender	health promotion	quality of life
Herbal medicine for insomnia in elderly with hypertension: A systematic review and meta-analysis.[237]	2019	general elderly, both gender	screening	sleep quality,
Hospital-at-home in older patients: A scoping review on opportunities of developing comprehensive geriatric assessment based services.[238]	2015	general elderly, both gender	health promotion & social support	quality of life,
How do different physical exercise parameters modulate brain-derived neurotrophic factor in healthy and non-healthy adults? A systematic review, meta-analysis and meta-regression.[239]	2019	general elderly, both gender	health promotion & screening	physical activity,
How do older adults experience and perceive socially assistive robots in aged care: a systematic review of qualitative evidence.[240]	2018	general public, both gender	health promotion & social support	psychosocial functioning,
How do views on aging affect health outcomes in adulthood and late life?: Explanations for an established connection.[241]	2017	general elderly, both gender	screening	frailty

How Exercise Influences Cognitive Performance When Mild Cognitive Impairment Exists: A Literature Review.[242]	2016	general elderly, both gender	health promotion & screening	disease oriented,
How to improve the quality of life of elderly people with intellectual disability: A systematic literature review of support strategies.[243]	2019	50+, both gender	screening	disease oriented,quality of life, disability
ICT-based applications to improve social health and social participation in older adults with dementia. A systematic literature review.[244]	2017	general elderly, both gender	health promotion	independence,disease oriented
Impact of behavioural dietary intervention coupled with oral rehabilitation on the health and nutritional status of older adults: A systematic review and meta-analysis.[245]	2019	general elderly, both gender	screening	general health
Impact of universal interventions on social inequalities in physical activity among older adults: an equity-focused systematic review.[246]	2017	50+, both gender	social support & screening	psychosocial functioning,
Impact of Vitamin C supplementation on Risk for Cardiovascular events: Future scenario for the elderly US population.[247]	2016	general elderly, both gender	primary prevention	disease oriented,
Impact of mind-body interventions in older adults with mild cognitive impairment: a systematic review.[248]	2019	55+, both gender	health promotion & primary prevention	independence,disease oriented, general health
Improved 1-year mortality in elderly patients with a hip fracture following integrated orthogeriatric treatment.[249]	2017	70+, both gender	health promotion & screening	quality of life,
Increasing physical activity for veterans in the Mental Health Intensive Case Management Program: A community-based intervention.[250]	2018	general elderly, both gender	screening	physical activity,quality of life
Influence of computer-based cognitive training on mobility in healthy older adults : A systematic review.[251]	2018	general elderly, both gender	health promotion	quality of life,

Influenza vaccination for healthcare workers who care for people aged 60 or older living in long-term care institutions.[252]	2016	60+, both gender	health promotion & primary prevention	disease oriented
Insomnia and risk of dementia in older adults: Systematic review and meta-analysis.[253]	2016	general elderly, both gender	health promotion & primary prevention	sleep quality,
Instrument Playing as a Cognitive Intervention Task for Older Adults: A Systematic Review and Meta-Analysis.[254]	2019	60+, both gender	health promotion & primary prevention	cognitive function
Integrated Care for Depression in Older Primary Care Patients.[255]	2018	general elderly, both gender	health promotion & screening	disease oriented
Internet-Based Interventions Aimed at Supporting Family Caregivers of People With Dementia: Systematic Review.[256]	2018	general elderly, both gender	health promotion & social support	disease oriented
Interventions based on exercise and physical environment for preventing falls in cognitively impaired older people living in long-term care facilities: A systematic review and meta-analysis.[257]	2016	general elderly, both gender	primary prevention & screening	disease oriented,frailty, cognitive function
Interventions designed to increase older people's exercise engagement: A systematic review.[258]	2016	general elderly, both gender	screening	physical activity
Interventions for preventing cognitive decline and dementia: Recommendations from a national academies of sciences, engineering, and medicine study.[259]	2017	general elderly, both gender	health promotion	cognitive function,
Interventions that support major life transitions in older adulthood: a systematic review.[260]	2019	50+, both gender	screening & social support	psychosocial functioning,
Interventions to reduce dependency in bathing in community dwelling older adults: a systematic review.[261]	2017	general elderly, both gender	screening	frailty,quality of life

Interventions for preventing elder abuse: applying findings of a new Cochrane review.[262]	2017	general elderly, both gender	primary prevention	quality of life
Interventions targeting loneliness and social isolation among the older people: An update systematic review.[263]	2017	general elderly, both gender	social support	quality of life,
Interventions to optimise prescribing for older people in care homes.[264]	2016	65+, both gender	health promotion & screening	disease oriented,
Interventions to optimize pharmacologic treatment in hospitalized older adults: a systematic review.[265]	2016	65+, both gender	health promotion	disease oriented
Interventions to Prevent Cognitive Decline and Dementia in Adults without Cognitive Impairment: A Systematic Review.[266]	2015	general elderly, both gender	primary prevention	cognitive function,
Is age a factor in the success or failure of remote monitoring in heart failure? Telemonitoring and structured telephone support in elderly heart failure patients.[267]	2015	70+, both gender	screening	disease oriented
Is exercise therapy effective in the older people? a systematic review.[268]	2015	general elderly, both gender	health promotion & screening	physical activity
Is vitamin D supplementation effective for the prevention of falls in elderly people?.[269]	2016	general elderly, both gender	health promotion	nutrition,
Issues of trial selection and subgroup considerations in the recent meta-analysis of Zhao and colleagues on fracture reduction by calcium and vitamin D supplementation in community-dwelling older adults.[270]	2018	50+, both gender	health promotion & primary prevention	nutrition,
Jigsaw puzzles as cognitive enrichment (PACE) - the effect of solving jigsaw puzzles on global visuospatial cognition in adults 50 years of age and older: Study protocol for a randomized controlled trial.[271]	2017	50+, both gender	health promotion & primary prevention	cognitive function,independence

Latent structure of the Centre for Epidemiologic Studies Depression Scale (CES-D) in older adult populations: a systematic review.[272]	2019	65+, both gender	screening	mental health,
Later Life Learning Experiences: Listening to the Voices of Chinese Elders in Hong Kong.[273]	2016	55-75, both gender	health promotion	quality of life,independence
Learning through Political Participation: A Case Study of Spanish Elders Involved in Political Organizations.[274]	2016	65+, both gender	social support & screening	psychosocial functioning,
Length of hospital stay and dementia: A systematic review of observational studies.[275]	2019	general elderly, both gender	screening	general health,
Life story books for people with dementia: a systematic review.[276]	2018	general elderly, both gender	screening & social support	disease oriented,
Lifelong Learning for Aging People in Taiwan: Innovative Programs and Social Effects.[277]	2019	55+, both gender	health promotion & screening & social support	independence,
Light therapy for older patients with non-seasonal depression: A systematic review and meta-analysis.[278]	2018	general elderly, both gender	health promotion & screening	mental health,
Loneliness and social isolation causal association with health-related lifestyle risk in older adults: a systematic review and meta-analysis protocol.[279]	2019	general elderly, both gender	health promotion & social support	general health,
Long-Term Aerobic Exercise Improves Vascular Function Into Old Age: A Systematic Review, Meta-Analysis and Meta Regression of Observational and Interventional Studies.[280]	2019	62-75, both gender	health promotion & primary prevention	physical activity
Low bone mineral density is the main contributor to falls-related health burden in the elderly.[281]	2017	50+, 女	screening	frailty,
Malnutrition in older adults: screening and determinants.[282]	2018	general elderly, both gender	screening	general health,

Management of Frailty: A Systematic Review and Network Meta-analysis of Randomized Controlled Trials.[283]	2019	general elderly, both gender	health promotion & primary prevention	frailty,general health, nutrition
Measurement of physical activity in older adult interventions: a systematic review.[284]	2015	65+, both gender	screening	physical activity
Measurement properties of patient-reported outcome measures (PROMs) for women with Genitourinary Syndrome of Menopause: a systematic review.[285]	2019	general elderly, both gender	screening	disease oriented,
Mediterranean Diet and Musculoskeletal-Functional Outcomes in Community-Dwelling Older People: A Systematic Review and Meta-Analysis.[286]	2018	60+, both gender	health promotion & primary prevention	disease oriented,frailty
'Memory in Two Voices:' An Aesthetics of Care in Performance Groups for Older Adults.[287]	2019	general elderly, both gender	health promotion	disease oriented,
Meta-analysis of aging effects in mind wandering: Methodological and sociodemographic factors.[288]	2019	general elderly, both gender	screening	frailty,mental health
Meta-analysis of problem solving therapy for the treatment of major depressive disorder in older adults.[289]	2016	60+, both gender	health promotion	mental health,
Meta-analysis of psychosocial interventions for people with dementia and anxiety or depression.[290]	2019	general elderly, both gender	health promotion & social support	disease oriented,psychosocial functioning, addiction
Mini‐Mental State Examination (MMSE) for the detection of dementia in clinically unevaluated people aged 65 and over in community and primary care populations.[291]	2016	65+, both gender	screening	cognitive function,

Mission accomplished? Active ageing in nursing homes: A new approach to enable meaningful activities and quality of life.[292]	2015	general elderly, both gender	health promotion	quality of life,
Mobile Health Interventions for Physical Activity, Sedentary Behavior, and Sleep in Adults Aged 50 Years and Older: A Systematic Literature Review.[293]	2018	50+, both gender	health promotion & primary prevention	sleep quality,quality of life
Movements of older adults during exergaming interventions that are associated with the Systems Framework for Postural Control: A systematic review.[294]	2018	general elderly, both gender	health promotion & primary prevention	frailty,
Multidisciplinary nutritional intervention during hospitalization and after discharge in elderly patients-a meta-analysis.[295]	2016	general elderly, both gender	health promotion & screening	general health,
Multifactorial assessment and targeted intervention to prevent recurrent falls in community dwelling elderly individuals presenting to emergency departments: A systematic review and meta-analysis.[296]	2016	general elderly, both gender	screening	frailty
Multifactorial interventions for preventing falls in older people living in the community: a systematic review and meta-analysis of 41 trials and almost 20 000 participants.[297]	2019	65+, both gender	primary prevention & screening	general health,
Multimorbidity and perceived stress: a population-based cross-sectional study among older adults across six low- and middle-income countries.[298]	2018	50+, both gender	primary prevention & screening	cognitive function,quality of life
Musical practice as an enhancer of cognitive function in healthy aging - A systematic review and meta-analysis.[299]	2018	59+, both gender	health promotion	frailty,
Music-based therapeutic interventions for people with dementia.[300]	2018	general elderly, both gender	health promotion	cognitive function,

Narrative Medicine: Community Poetry Heals Young and Old.[301]	2017	general elderly, both gender	health promotion & social support	general health,quality of life
Network Meta-Analysis of Various Nonpharmacological Interventions on Pain Relief in Older Adults With Osteoarthritis.[302]	2019	60+, both gender	health promotion	physical activity,
New ways to count sleep: A review of suvorexant for insomnia in older adults.[303]	2017	general elderly, both gender	health promotion & primary prevention	sleep quality,
No Place Like Home: A Systematic Review of Home Care for Older Adults in Canada.[304]	2018	general elderly, both gender	health promotion & social support	independence
Non-Pharmacologic Interventions for Older Adults with Subjective Cognitive Decline: Systematic Review, Meta-Analysis, and Preliminary Recommendations.[305]	2017	general elderly, both gender	health promotion & screening	disease oriented,general health
Nonpharmacological approaches for behavioural and psychological symptoms of dementia in older adults: A systematic review of reviews.[306]	2018	general elderly, both gender	health promotion	mental health,
Non-pharmacological strategies to reduce blood pressure in older adults: A systematic review and meta-analysis.[307]	2017	65+, both gender	health promotion	physical activity,
Novel aquatic physiotherapy programme for elderly Chinese adults with osteoarthritis of the knee: A randomised controlled trial.[308]	2017	55+, both gender	health promotion & primary prevention	physical activity,frailty
Nurse-Led Care Models in the Context of Community Elders With Chronic Disease Management: A Systematic Review.[309]	2016	general elderly, both gender	social support & screening	general health,
Nutrition and Bipolar Disorders in Older Adults: A systematic Review.[310]	2019	general elderly, both gender	social support & screening	mental health,

Nutrition, or Nutrition and Exercise? A Systematic Review of Interventions in Frail Elderly People.[311]	2015	general elderly, both gender	health promotion & screening	physical activity,general health
Nutritional interventions and cognitive-related outcomes in patients with late-life cognitive disorders: A systematic review.[312]	2018	60+, both gender	health promotion & screening	nutrition,cognitive function
Nutritional risk factors, microbiota and parkinson's disease: What is the current evidence?[313]	2019	general elderly, both gender	health promotion & screening	nutrition,
Nutritional Strategies in the Management of Alzheimer Disease: Systematic Review With Network Meta-Analysis.[314]	2017	general elderly, both gender	health promotion & screening	nutrition,
On the Life Aesthetics of Aging People--Slowness Makes Life Beautiful.[315]	2015	other, both gender	ALL	quality of life,general health
Oral Health and Cognitive Function in Older Adults: A Systematic Review.[316]	2019	general elderly, both gender	health promotion & primary prevention	cognitive function,
Oral nutritional support with or without exercise in the management of malnutrition in nutritionally vulnerable older people: A systematic review and meta-analysis.[317]	2017	general elderly, both gender	health promotion	physical activity,
Orthostatic hypotension and symptomatic subclinical orthostatic hypotension increase risk of cognitive impairment: An integrated evidence review and analysis of a large older adult hypertensive cohort.[318]	2018	80+, both gender	screening	cognitive function,
Overview of community-based studies of depression screening interventions among the elderly population in Japan.[319]	2016	general elderly, both gender	primary prevention & screening & social support	disease oriented,mental health
Pain-Reducing Effects of Physical Therapist-Delivered Interventions: A Systematic Review of Randomized Trials Among Older Adults With Dementia.[320]	2019	60+, both gender	health promotion	disease oriented

Pharmacotherapy for hypertension in adults 60 years or older.[321]	2019	60+, both gender	health promotion	disease oriented
Physical activity programs for balance and fall prevention in elderly: A systematic review.[322]	2019	65+, both gender	primary prevention	physical activity,
Physical activity and cognitive function among older adults in China: A systematic review.[323]	2016	general elderly, both gender	health promotion & primary prevention	physical activity,
Physical activity and loneliness among adults aged 50 years or older in six low- and middle-income countries.[324]	2019	50+, both gender	health promotion & social support	physical activity,
Physical Activity in Community Dwelling Older People: A Systematic Review of Reviews of Interventions and Context.[325]	2017	general elderly, both gender	health promotion	general health,
Physical activity monitors to enhance amount of physical activity in older adults - a systematic review and meta-analysis.[326]	2019	65+, both gender	primary prevention & screening	physical activity
Physical activity monitors to enhance the daily amount of physical activity in elderly-a protocol for a systematic review and meta-analysis.[327]	2018	65+, both gender	health promotion & screening	physical activity
Physical Activity Throughout the Adult Life Span and Domain-Specific Cognitive Function in Old Age: A Systematic Review of Cross-Sectional and Longitudinal Data.[328]	2018	60+, both gender	health promotion & screening	cognitive function,
Physical and Psychological Health Outcomes of Qigong Exercise in Older Adults: A Systematic Review and Meta-Analysis.[329]	2019	62-83, both gender	screening	disease oriented,general health
Physical exercise in osteoporotic fracture prevention in older adults.[330]	2016	50+，女	health promotion & primary prevention & screening	disease oriented,frailty
Post-traumatic stress disorder in older adults: a systematic review of the psychotherapy treatment literature.[331]	2015	55+, both gender	health promotion	mental health

Potentially modifiable determinants of malnutrition in older adults: A systematic review.[332]	2019	general elderly, both gender	screening	nutrition,
Predicting risk and outcomes for frail older adults: A protocol for an umbrella review of available frailty screening tools.[333]	2015	general elderly, both gender	screening	frailty
Predictive and Reactive Locomotor Adaptability in Healthy Elderly: A Systematic Review and Meta-Analysis.[334]	2015	60+, both gender	screening	physical activity
Predictors of treatment outcome in depression in later life: A systematic review and meta-analysis.[335]	2018	60+, both gender	health promotion & screening	psychosocial functioning,mental health
Prevalence and correlates of major depressive disorder, bipolar disorder and schizophrenia among nursing home residents without dementia: systematic review and meta-analysis.[336]	2019	general elderly, both gender	health promotion & screening & social support	disease oriented,
Preventing delirium: Should non-pharmacological, multicomponent interventions be used? A systematic review and meta-analysis of the literature.[337]	2015	general elderly, both gender	primary prevention & screening	mental health,
Prevention of sexually transmitted and blood borne infections among older adults.[338]	2015	general elderly, both gender	primary prevention & screening	disease oriented
Prevention of stroke in the elderly with nonvalvular atrial fibrillation: A systematic review and meta-analysis of randomized controlled trials comparing novel oral anticoagulants and left atrial appendage occlusion device versus warfarin.[339]	2015	general elderly, both gender	primary prevention & screening	disease oriented
Preventive Interventions for the Second Half of Life: A Systematic Review.[340]	2017	50+, both gender	primary prevention	general health
Primary prevention of dementia: from modifiable risk factors to a public brain health agenda?[341]	2018	general elderly, both gender	primary prevention	general health,physical activity

Prognostic significance of social network, social support and loneliness for course of major depressive disorder in adulthood and old age.[342]	2018	60+ vs 60-, both gender	health promotion & screening	quality of life,psychosocial functioning
Programs to combat loneliness in the institutionalised elderly: A review of the scientific literature.[343]	2018	general elderly, both gender	screening	quality of life
Protein Supplementation Does Not Significantly Augment the Effects of Resistance Exercise Training in Older Adults: A Systematic Review.[344]	2016	77.4, both gender	health promotion & screening	nutrition,
Psychological Treatment of Depression in People Aged 65 Years and Over: A Systematic Review of Efficacy, Safety, and Cost-Effectiveness.[345]	2016	65+, both gender	screening	mental health,
Psychosocial interventions for people with dementia: a synthesis of systematic reviews.[346]	2019	general elderly, both gender	health promotion & screening	psychosocial functioning,general health
Qualitative studies of psychosocial interventions for dementia: a systematic review.[347]	2015	general elderly, both gender	health promotion & screening	psychosocial functioning,
Quality of life and psychological consequences in elderly patients after a hip fracture: a review.[348]	2018	65+, both gender	screening	disease oriented,general health, quality of life
Quality of life in dementia: a systematically conducted narrative review of dementia-specific measurement scales.[349]	2015	general elderly, both gender	screening	disease oriented,
Quantitative Mobility Assessment for Fall Risk Prediction in Dementia: A Systematic Review.[350]	2018	general elderly, both gender	primary prevention	frailty,
Recognizing and Nurturing Giftedness in Gifted Elders.[351]	2018	general elderly, both gender	health promotion	independence,
Reducing social isolation and loneliness in older people: a systematic review protocol.[352]	2017	60+, both gender	health promotion & primary prevention	quality of life,

Refresher course: Regional anesthesia to avoid postoperative cognitive dysfunction: What is the evidence?[353]	2015	general elderly, both gender	primary prevention & screening	cognitive function
Rehabilitation with sleep? Results from a systematic review on motor learning and sleep in the aging population and after stroke.[354]	2015	general elderly, both gender	health promotion & screening	disease oriented,physical activity, independence
Relationship between sarcopenia and the incidence of fall in elderly-a systematic review of advanced nutritional and physical interventions studies.[355]	2017	general elderly, both gender	health promotion & screening	disease oriented,frailty
Relationships of Dietary Patterns, Foods, and Micro- and Macronutrients with Alzheimer's Disease and Late-Life Cognitive Disorders: A Systematic Review.[356]	2017	general elderly, both gender	health promotion	nutrition,cognitive function
Relevance of a Geriatric Assessment for Elderly Patients With Lung Cancer-A Systematic Review.[357]	2016	73-81, both gender	primary prevention & screening	general health,cognitive function, quality of life
Relevance of vitamin D in fall prevention.[358]	2017	general elderly, both gender	primary prevention	nutrition,
Relevant outcomes for nutrition interventions to treat and prevent malnutrition in older people: a collaborative senator-on top and manuel delphi study.[359]	2018	general elderly, both gender	health promotion & primary prevention	nutrition
Review of Programs to Combat Elder Mistreatment: Focus on Hospitals and Level of Resources Needed.[360]	2019	general elderly, both gender	health promotion & primary prevention & social support	quality of life
Risk factors and protective factors associated with incident or increase of frailty among community-dwelling older adults: A systematic review of longitudinal studies.[361]	2017	60+, both gender	health promotion & screening	general health,
Risk factors for sleep disturbances in older adults: Evidence from prospective studies.[362]	2015	general elderly, both gender	screening	frailty,

Role of dietary protein and thiamine intakes on cognitive function in healthy older people: a systematic review.[363]	2015	general elderly, both gender	screening	cognitive function,
Role of fatty acids and micronutrients in healthy ageing: a systematic review of randomised controlled trials set in the context of European dietary surveys of older adults.[364]	2015	general elderly, both gender	screening	frailty,
Sarcopenic obesity and complex interventions with nutrition and exercise in community-dwelling older persons--a narrative review.[365]	2015	general elderly, both gender	health promotion & screening	nutrition,disease oriented, independence
Scoping review of health promotion and disease prevention interventions addressed to elderly people.[366]	2016	55+, both gender	health promotion & primary prevention	frailty,
Scoping review on the use of socially assistive robot technology in elderly care.[367]	2018	general elderly, both gender	health promotion & social support	psychosocial functioning,cognitive function
Screening for Elder Abuse among Turkish Older People: Validity of the Hwalek-Sengstock Elder Abuse Screening Test (H-S "East")[368]	2017	60+, both gender	screening	general health,
Screening of nutritional status among elderly people at family medicine.[369]	2015	general elderly, both gender	health promotion & screening	nutrition
Screening for oropharyngeal dysphagia in older adults: A systematic review of self-reported questionnaires.[370]	2018	general elderly, both gender	health promotion & screening	nutrition,
Screening for vestibular schwannoma in the context of an ageing population.[371]	2019	70+, both gender	screening	disease oriented
Screening tools for identification of elder abuse: a systematic review.[372]	2017	general elderly, both gender	health promotion & screening	quality of life

Self-Report Tools for Assessing Physical Activity in Community-Living Older Adults with Multiple Chronic Conditions: A Systematic Review of Psychometric Properties and Feasibility.[373]	2019	general elderly, both gender	health promotion & screening	disease oriented,physical activity, mental health
Seniors centre-based health intervention programmes in the United States and South Korea: A systematic review.[374]	2017	general elderly, both gender	health promotion	general health
Seniors' Demographic Correlates for Motivations to Enroll in Degree-Conferring Programs in Universities.[375]	2016	60+, both gender	social support & screening	independence,
Service Delivery Models to Maximize Quality of Life for Older People at the End of Life: A Rapid Review.[376]	2019	general elderly, both gender	health promotion	quality of life
Similarities and Differences in Views of Ageing and Learning in Hong Kong and Australia.[377]	2017	55+, both gender	screening	independence,
Sleep Duration in Relation to Cognitive Function among Older Adults: A Systematic Review of Observational Studies.[378]	2016	general elderly, both gender	health promotion	sleep quality,
Smart homes and home health monitoring technologies for older adults: A systematic review.[379]	2016	general elderly, both gender	health promotion	general health,
Smoking cessation in elderly: Is there a better way?[380]	2016	general elderly, both gender	health promotion	addiction
Social capital interventions targeting older people and their impact on health: a systematic review.[381]	2017	general elderly, both gender	health promotion	general health,
Social isolation, chronic diseases and prevention in old age.[382]	2019	general elderly, both gender	social support & screening	psychosocial functioning,quality of life
Social network typologies and mortality risk among older people in China, India, and Latin America: A 10/66 Dementia Research Group population-based cohort study.[383]	2015	65+, both gender	screening & social support	disease oriented,general health

Socio-economic position and subjective health and well-being among older people in Europe: a systematic narrative review.[384]	2016	general elderly, both gender	ALL	general health,
Statins for Primary Prevention of Cardiovascular Disease in Elderly Patients: Systematic Review and Meta-Analysis.[385]	2015	65+, both gender	primary prevention	disease oriented
Step training improves reaction time, gait and balance and reduces falls in older people: a systematic review and meta-analysis.[386]	2016	60+, both gender	health promotion & primary prevention	physical activity,
Strategies employed by older people to manage loneliness: systematic review of qualitative studies and model development.[387]	2018	general elderly, both gender	screening & social support	quality of life,
Strategy-Based Cognitive Training for Improving Executive Functions in Older Adults: a Systematic Review.[388]	2016	general elderly, both gender	screening	general health,cognitive function
Structural brain plasticity induced by physical training in adults affected by aging or disease related impairments: A systematic review.[389]	2015	general elderly, both gender	health promotion	diseased oriented,cognitive function
Supplemental protein from dairy products increases body weight and vitamin D improves physical performance in older adults: A systematic review and meta-analysis.[390]	2018	55+, both gender	health promotion & screening	general health,
Surgical versus nonsurgical management for type II odontoid fractures in the elderly population: a systematic review.[391]	2018	65+, both gender	screening	disease oriented
Systematic Literature Review and Meta-Analysis of the Association of Sarcopenia With Mortality.[392]	2016	general elderly, both gender	screening	disease oriented
Systematic literature review of determinants of sedentary behaviour in older adults: a DEDIPAC study.[393]	2015	general elderly, both gender	health promotion & screening	general health
Systematic review and meta-analyses of useful field of view cognitive training.[394]	2018	60+, both gender	health promotion & screening	cognitive function,

Systematic review and meta-analysis of the effect of protein and amino acid supplements in older adults with acute or chronic conditions.[395]	2018	general elderly, both gender	primary prevention & screening	diseased oriented,
Systematic review of behaviour change techniques to promote participation in physical activity among people with dementia.[396]	2018	general elderly, both gender	health promotion & primary prevention	physical activity,psychosocial functioning
Systematic Review of Statins Effectiveness in Prevention Secondary in Elderly.[397]	2017	general elderly,	primary prevention	disease oriented
Systematic Review of the Effects of Cognitive and Behavioural Interventions on Fall-Related Psychological Concerns in Older Adults.[398]	2019	general elderly, both gender	screening	cognitive function,mental health
Systematic review of the efficacy of non-pharmacological interventions in people with Lewy body dementia.[399]	2018	general elderly, both gender	health promotion & screening	disease oriented
Systematic review of the impact of arts for health activities on health, wellbeing and quality of life of older people living in care homes.[400]	2018	general elderly, both gender	health promotion	general health,quality of life, psychosocial functioning
Systematic Review and Meta-Analysis of the Association between Sarcopenia and Dysphagia.[401]	2018	general elderly, both gender	screening	frailty,
Systematic review of nutritional screening methods for elderly Brazilians living at home.[402]	2019	general elderly, both gender	screening	nutrition
Systolic blood pressure target for cardiovascular prevention in the elderly-a meta-analysis of randomized controlled trials.[403]	2016	general elderly, both gender	primary prevention	general health,
Tai Chi as an intervention on health promotion for older adults: A systematic review.[404]	2015	general elderly, both gender	health promotion	general health,

Taichi exercise for self-rated sleep quality in older people: a systematic review and meta-analysis.[405]	2015	general elderly, both gender	health promotion	general health,sleep quality
Telehealth in the Elderly with Chronic Heart Failure: What Is the Evidence?[406]	2018	70+, both gender	health promotion	disease oriented
The Application of Virtual Reality in Geriatric Mental Health: The State of the Evidence.[407]	2019	general elderly, both gender	health promotion	cognitive function,general health
The art of successful implementation of psychosocial interventions in residential dementia care: a systematic review of the literature based on the RE-AIM framework.[408]	2015	general elderly, both gender	health promotion & screening & social support	general health,cognitive function
The assessment of anxiety in aged care residents: a systematic review of the psychometric properties of commonly used measures.[409]	2018	general elderly, both gender	screening	mental health
The Association Between Gait Speed and Cognitive Status in Community-Dwelling Older People: A Systematic Review and Meta-analysis.[410]	2018	general elderly, both gender	health promotion & screening	cognitive function,
The association between medication non-adherence and adverse health outcomes in ageing populations: A systematic review and meta-analysis.[411]	2019	50+, both gender	health promotion	disease oriented
The association between physical frailty and polypharmacy in community dwelling older adults.[412]	2019	65+, both gender	screening	quality of life,
The association between SI treatment and suicidality in elderly patients with major depression: A systematic review.[413]	2015	general elderly, both gender	health promotion & primary prevention & screening	mental health,
The association between social support and physical activity in older adults: a systematic review.[414]	2017	60+, both gender	health promotion & social support	psychosocial functioning,

The association of late-life depression with all-cause and cardiovascular mortality among community-dwelling older adults: systematic review and meta-analysis.[415]	2019	general elderly, both gender	screening	disease oriented,mental health
The burden of respiratory infections among older adults in long-term care: a systematic review.[416]	2019	60+, both gender	health promotion & screening	disease oriented
The Effect of a Meditative Movement Intervention on Quality of Sleep in the Elderly: A Systematic Review and Meta-Analysis.[417]	2015	general elderly, both gender	health promotion	sleep quality
The effect of diet, lifestyle and/or cognitive interventions in Mild Cognitive Impairment: A systematic review.[418]	2017	general elderly, both gender	screening	nutrition,general health, cognitive function
The Effect of Information Communication Technology Interventions on Reducing Social Isolation in the Elderly: A Systematic Review.[419]	2016	general elderly, both gender	health promotion & social support	quality of life,
The effect of music on postoperative recovery in older patients: A systematic review.[420]	2018	60+, both gender	health promotion & social support	quality of life,
The effect of music therapy on cognitive functions in patients with dementia: a systematic review and meta-analysis.[421]	2018	78-87, both gender	screening & social support	cognitive function,general health
The Effect of Physical Exercise on Frail Older Persons: A Systematic Review.[422]	2017	general elderly, both gender	health promotion	frailty,
The effect of resistance training on cognitive function in the older adults: a systematic review of randomized clinical trials.[423]	2018	general elderly, both gender	health promotion	cognitive function,general health
The effect of sleep on motor learning in the aging and stroke population - a systematic review.[424]	2016	general elderly, both gender	health promotion	disease oriented,physical activity
The effect of spinal braces on balance of elderly subjects with thoracic hyperkyphosis: Protocol for a systematic review.[425]	2018	general elderly, both gender	health promotion	disease oriented,

The effectiveness of nonpharmacological interventions in older adults with depressive disorders: a systematic review.[426]	2015	general elderly, both gender	health promotion & screening	disease oriented,
The effectiveness of patient education in improving pain, disability and quality of life among older people with low back pain: A systematic review.[427]	2019	general elderly, both gender	screening	disability,quality of life, disease oriented
The effectiveness of peer-based interventions on health promoting behaviors in older people: A systematic review protocol of quantitative evidence.[428]	2015	general elderly, both gender	health promotion & social support	general health,
The effectiveness of Tai Chi Chuan in osteoarthritis of the knee: A systematic review.[429]	2019	general elderly, both gender	health promotion & screening	physical activity,
The effectiveness of Tai Chi for short-term cognitive function improvement in the early stages of dementia in the elderly: a systematic literature review.[430]	2019	general elderly, both gender	health promotion	cognitive function,physical activity, general health
The Effectiveness of Technology-Mediated Dance Interventions and Their Impact on Psychosocial Factors in Older Adults: A Systematic Review and Meta-Analysis.[431]	2018	65+, both gender	health promotion & social support	physical activity,general health, psychosocial functioning
The Effects of Mind-Body Exercise on Cognitive Performance in Elderly: A Systematic Review and Meta-Analysis.[432]	2019	general elderly, both gender	health promotion	cognitive function,
The effects of physical exercise on executive function in community-dwelling older adults living with Alzheimer's-type dementia: A systematic review.[433]	2018	general elderly, both gender	primary prevention	physical activity,
The effects of Pilates exercise training on physical fitness and wellbeing in the elderly: A systematic review for future exercise prescription.[434]	2015	60-80, both gender	health promotion & screening	quality of life,

The effects of Qigong exercises on blood lipid profiles of middle-aged and elderly individuals: A systematic review and network meta-analysis.[435]	2019	general elderly, both gender	health promotion & screening	physical activity,
The effects of the Pilates method in the elderly: a systematic review.[436]	2016	60+, both gender	health promotion	general health,
The Effects of the Pilates Training Method on Balance and Falls of Older Adults: A Systematic Review and Meta-Analysis of Randomized Controlled Trials.[437]	2017	60+, both gender	health promotion & primary prevention	frailty,
The Efficacy and Safety of Lower-Limb Plyometric Training in Older Adults: A Systematic Review.[438]	2019	general elderly, both gender	health promotion & screening	frailty,
The efficacy of cognitive intervention programs for mild cognitive impairment: A systematic review.[439]	2015	general elderly, both gender	screening	cognitive function,
The exercise effect on psychological well-being in older adults - A systematic review of longitudinal studies.[440]	2018	general elderly, both gender	health promotion & screening	mental health,
The features of interventions associated with long-term effectiveness of physical activity interventions in adults aged 55-70 years: a systematic review and meta-analysis.[441]	2015	55-70, both gender	health promotion & screening	general health,
The impact of cognitive interventions on cognitive symptoms in idiopathic Parkinson's disease: a systematic review.[442]	2019	general elderly, both gender	screening	disease oriented,general health
The impact of dietary factors on indices of chronic disease in older people: A systematic review.[443]	2018	65+, both gender	screening	disease oriented,
The impact of dietary protein or amino acid supplementation on muscle mass and strength in elderly people: Individual participant data and meta-analysis of RCT’s.[444]	2017	general elderly, both gender	health promotion	frailty,quality of life

The impact of memory-strategy training interventions on participant-reported outcomes in healthy older adults: A systematic review and meta-analysis.[445]	2019	general elderly, both gender	health promotion & screening	quality of life,frailty
The Impact of Quality of Life on the Health of Older People from a Multidimensional Perspective.[446]	2018	general elderly, both gender	ALL	general health,
The influence of occupation on wellbeing, as experienced by the elderly: a systematic review.[447]	2018	65+, both gender	health promotion & screening	quality of life,
The Mental Health of Older Persons After Human-Induced Disasters: A Systematic Review and Meta-Analysis of Epidemiological Data.[448].	2016	general elderly, both gender	health promotion & screening & social support	quality of life,
The neighbourhood physical environment and active travel in older adults: a systematic review and meta-analysis.[449]	2017	65+, both gender	ALL	physical activity,mental health
The occurrence, risk factors, and prevention of contrast-induced nephropathy in the elderly.[450]	2017	general elderly, both gender	primary prevention	disease oriented
The optimal treatment for improving cognitive function in elder people with mild cognitive impairment incorporating Bayesian network meta-analysis and systematic review.[451]	2019	general elderly, both gender	health promotion & screening	cognitive function,
The personal active aging strategies of older adults in Europe: a systematic review of qualitative evidence.[452]	2016	55+, both gender	ALL	physical activity,quality of life, frailty
The relationship between sarcopenia and fragility fracture-a systematic review.[453]	2019	general elderly, both gender	health promotion & screening	frailty,
The relationship between physical activity, appetite and energy intake in older adults: A systematic review.[454]	2018	general elderly, both gender	health promotion & screening	nutrition,

The relationship of nutrition to cognitive function in older adults: A systematic review of RCTs.[455]	2015	general elderly, both gender	health promotion & screening	cognitive function,
The Role of Muscle Mass Gain Following Protein Supplementation Plus Exercise Therapy in Older Adults with Sarcopenia and Frailty Risks: A Systematic Review and Meta-Regression Analysis of Randomized Trials.[456]	2019	general elderly, both gender	health promotion & primary prevention	nutrition,frailty, disease oriented
The role of nutritional interventions in increasing fruit and vegetable intake in the elderlies: a systematic review.[457]	2015	general elderly, both gender	screening	nutrition
The Use of Motion-Based Technology for People Living With Dementia or Mild Cognitive Impairment: A Literature Review.[458]	2017	general elderly, both gender	health promotion & primary prevention	disease oriented,general health, independence
The Use of Tablet Technology by Older Adults in Health Care Settings-Is It Effective and Satisfying? A Systematic Review and Meta Analysis.[459]	2017	65+, both gender	health promotion	general health,
The use of technology for mental healthcare delivery among older adults with depressive symptoms: A systematic literature review.[460]	2019	general elderly, both gender	health promotion & screening & social support	mental health,
THE VIBRATION TRAINING AS SARCOPENIA INTERVENTION: IMPACT ON THE NEUROMUSCULAR SYSTEM OF THE ELDERLY.[461]	2015	general elderly, both gender	health promotion	physical activity,frailty
The Effectiveness of Horticultural Therapy on Older Adults: A Systematic Review.[462]	2019	60+, both gender	health promotion & screening	quality of life,
The effectiveness of pneumococcal polysaccharide vaccine 23 (PPV23) in the general population of 50 years of age and older: A systematic review and meta-analysis.[463]	2016	50+, both gender	primary prevention	disease oriented

The Impact of Different Types of Exercise Training on Peripheral Blood Brain-Derived Neurotrophic Factor Concentrations in Older Adults: A Meta-Analysis.[464]	2019	60+, both gender	health promotion	disease oriented,
Theory-Based Interventions to Improve Medication Adherence in Older Adults Prescribed Polypharmacy: A Systematic Review.[465]	2017	65+, both gender	health promotion & screening	disease oriented,
Thyroidectomy for thyroid cancer in the elderly: A meta-analysis.[466]	2019	60+, both gender	health promotion	disease oriented
Tobacco smoking and risk for dementia: evidence from the 10/66 population-based longitudinal study.[467]	2019	65+, both gender	screening	addiction,
Tooth Loss Increases the Risk of Diminished Cognitive Function: A Systematic Review and Meta-analysis.[468]	2016	general elderly, both gender	screening	cognitive function,
Transitions between frailty states among community-dwelling older people: A systematic review and meta-analysis.[469]	2019	60+, both gender	screening	frailty
Treating trauma-related disorders in later life.[470]	2019	55+, both gender	screening	mental health,
Treatment of obesity in older persons-A systematic review.[471]	2019	60+, both gender	health promotion	disease oriented
Treatment of xerostomia and hyposalivation in the elderly: A systematic review.[472]	2016	general elderly, both gender	health promotion	disease oriented
Use of Music Therapy to Improve Cognition in Older Adults With Dementia: An Integrative Review.[473]	2019	general elderly, both gender	health promotion	cognitive function,
Use of Social Robots in Mental Health and Well-Being Research: Systematic Review.[474]	2019	general elderly, both gender	health promotion	general health,
Use of socially assistive robot technology in elderly care.[475]	2017	general elderly, both gender	health promotion & social support	general health
Using exercise to fight depression in older adults. A systematic review and meta-analysis.[476]	2015	60+, both gender	health promotion & screening	physical activity,

Using the cumulative model of patient complexity to understand supported self management in older people living with multi morbidity and cancer: A theory-led qualitative systematic review.[477]	2019	general elderly, both gender	screening	independence,
Utility of Probiotics for Maintenance or Improvement of Health Status in Older People - A Scoping Review.[478]	2019	60+, both gender	health promotion	general health,
Virtual reality therapy for rehabilitation of balance in the elderly: a systematic review and META-analysis.[479]	2019	general elderly, both gender	health promotion & primary prevention	disease oriented,
Vitamin and mineral supplementation for preventing dementia or delaying cognitive decline in people with mild cognitive impairment.[480]	2018	general elderly, both gender	primary prevention	disease oriented,cognitive function
What is the effect of health coaching on physical activity participation in people aged 60 years and over? A systematic review of randomised controlled trials.[481]	2017	60+, both gender	health promotion & screening	physical activity
What is the impact on health and wellbeing of interventions that foster respect and social inclusion in community-residing older adults? A systematic review of quantitative and qualitative studies.[482]	2018	60+, both gender	screening & social support	general health,
What to say if your patients ask about tai chi: Evidence-based response.[483]	2017	general elderly, both gender	health promotion	physical activity
Which interventions are effective in improving nutritional status in elderly/frail patients with CKD stage 3b-5d? a preliminary report by EBPG group.[484]	2016	65+, both gender	health promotion & screening	nutrition
Which nutrients may contribute to healthy aging? A systematic review.[485]	2016	general elderly, both gender	health promotion	nutrition

Yoga-based exercise improves health-related quality of life and mental well-being in older people: a systematic review of randomised controlled trials.[486]	2018	60+, both gender	health promotion	quality of life, mental health

1. Garcia, J.A., et al., *A Bespoke Kinect Stepping Exergame for Improving Physical and Cognitive Function in Older People: A Pilot Study.* Games Health J, 2016. **5**(6): p. 382-388.
2. de van der Schueren, M.A., et al., *A critical appraisal of nutritional intervention studies in malnourished, community dwelling older persons.* Clin Nutr, 2016. **35**(5): p. 1008-14.
3. Kim, S.H. and S. Park, *A Meta-Analysis of the Correlates of Successful Aging in Older Adults.* Res Aging, 2017. **39**(5): p. 657-677.
4. Chang, Q.S., C.H. Chan, and P.S.F. Yip, *A meta-analytic review on social relationships and suicidal ideation among older adults.* Social Science & Medicine, 2017. **191**: p. 65-76.
5. Gwyther, H., et al., *A realist review to understand the efficacy and outcomes of interventions designed to minimise, reverse or prevent the progression of frailty.* Health Psychol Rev, 2018. **12**(4): p. 382-404.
6. McHugh, J., et al., *A Shared Mealtime Approach to Improving Social and Nutritional Functioning Among Older Adults Living Alone: Study Protocol for a Randomized Controlled Trial.* Jmir Research Protocols, 2015. **4**(2): p. e43.
7. Vaportzis, E., M.A. Niechcial, and A.J. Gow, *A systematic literature review and meta-analysis of real-world interventions for cognitive ageing in healthy older adults.* Ageing Res Rev, 2019. **50**: p. 110-130.
8. Ayalon, L., et al., *A systematic review and meta-analysis of interventions designed to prevent or stop elder maltreatment.* Age Ageing, 2016. **45**(2): p. 216-27.
9. Tahmosybayat, R., et al., *A systematic review and meta-analysis of outcome measures to assess postural control in older adults who undertake exergaming.* Maturitas, 2017. **98**: p. 35-45.
10. Anaturk, M., et al., *A systematic review and meta-analysis of structural magnetic resonance imaging studies investigating cognitive and social activity*

levels in older adults. Neuroscience and Biobehavioral Reviews, 2018. **93**: p. 71-84.
11. Marshall, S., et al., *A systematic review and meta-analysis of the criterion validity of nutrition assessment tools for diagnosing protein-energy malnutrition in the older community setting (the MACRo study).* Clin Nutr, 2018. **37**(6 Pt A): p. 1902-1912.
12. Khan, A.H., et al., *Oral Abstracts.* International Journal of Pharmacy Practice, 2019. **27**(S2): p. 4-26.
13. Guo, M., M.J.A. Stensland, and M. Health, *A systematic review of correlates of depression among older Chinese and Korean immigrants: What we know and do not know.* 2018. **22**(12): p. 1535-1547.
14. Shields, G.E., J. Elvidge, and L.M. Davies, *A systematic review of economic evaluations of seasonal influenza vaccination for the elderly population in the European Union.* BMJ Open, 2017. **7**(6): p. e014847.
15. Rapoport, M.J., et al., *A systematic review of intervention approaches for driving cessation in older adults.* Int J Geriatr Psychiatry, 2017. **32**(5): p. 484-491.
16. Canada, K.E., et al., *A systematic review of interventions for older adults living in jails and prisons.* Aging Ment Health, 2020. **24**(7): p. 1019-1027.
17. Okolie, C., et al., *A systematic review of interventions to prevent suicidal behaviors and reduce suicidal ideation in older people.* Int Psychogeriatr, 2017. **29**(11): p. 1801-1824.
18. Wang, G., A. Albayrak, and T.J.M. van der Cammen, *A systematic review of non-pharmacological interventions for BPSD in nursing home residents with dementia: from a perspective of ergonomics.* Int Psychogeriatr, 2019. **31**(8): p. 1137-1149.
19. Yen, H.Y. and L.J. Lin, *A Systematic Review of Reminiscence Therapy for Older Adults in Taiwan.* J Nurs Res, 2018. **26**(2): p. 138-150.
20. Shishehgar, M., D. Kerr, and J.J.S.H. Blake, *A systematic review of research into how robotic technology can help older people.* 2018. **7**: p. 1-18.
21. Abrilahij, A. and T.J.B.T. Boll, Ferring D Valsiner J, editiors. Cultures of care in aging. Charlotte : Information Age Publishing, *A systematic review of self-care assistive technologies for aging population.* 2018: p. 273-313.
22. Bingham, K.S., et al., *A Systematic Review of the Measurement of Function in Late-Life Depression.* Am J Geriatr Psychiatry, 2018. **26**(1): p. 54-72.
23. McWilliams, L., et al., *A systematic review of the prevalence of comorbid cancer and dementia and its implications for cancer-related care.* Aging Ment Health, 2018. **22**(10): p. 1254-1271.
24. Fearing, G., et al., *A systematic review on community-based interventions for elder abuse and neglect.* J Elder Abuse Negl, 2017. **29**(2-3): p. 102-133.
25. Dillon, L., et al., *A systematic review and meta-analysis of exercise-based falls prevention strategies in adults aged 50+ years with visual impairment.*

Ophthalmic Physiol Opt, 2018. **38**(4): p. 456-467.
26. Steenstra, I., et al., *A systematic review of interventions to promote work participation in older workers.* J Safety Res, 2017. **60**: p. 93-102.
27. Dam, A.E., et al., *A systematic review of social support interventions for caregivers of people with dementia: Are they doing what they promise?* 2016. **85**: p. 117-130.
28. Stojan, R. and C. Voelcker-Rehage, *A Systematic Review on the Cognitive Benefits and Neurophysiological Correlates of Exergaming in Healthy Older Adults.* J Clin Med, 2019. **8**(5): p. 734.
29. Liu, F., et al., *Acupoint Massage for Managing Cognitive Alterations in Older Adults: A Systematic Review and Meta-Analysis.* J Altern Complement Med, 2018. **24**(6): p. 532-540.
30. Gong, Z., et al., *Acutherapy for Knee Osteoarthritis Relief in the Elderly: A Systematic Review and Meta-Analysis.* Evid Based Complement Alternat Med, 2019. **2019**: p. 1868107.
31. Veazie, S., et al., *Addressing Social Isolation To Improve the Health of Older Adults: A Rapid Review.* 2019.
32. Farrance, C., F. Tsofliou, and C. Clark, *Adherence to community based group exercise interventions for older people: A mixed-methods systematic review.* Prev Med, 2016. **87**: p. 155-166.
33. Jankovic, N., et al., *Adherence to the WCRF/AICR Dietary Recommendations for Cancer Prevention and Risk of Cancer in Elderly from Europe and the United States: A Meta-Analysis within the CHANCES Project.* Cancer Epidemiol Biomarkers Prev, 2017. **26**(1): p. 136-144.
34. Zheng, G., et al., *Aerobic exercise ameliorates cognitive function in older adults with mild cognitive impairment: a systematic review and meta-analysis of randomised controlled trials.* Br J Sports Med, 2016. **50**(23): p. 1443-1450.
35. Cammisuli, D., et al., *Aerobic exercise effects upon cognition in Alzheimer's disease: a systematic review of randomized controlled trials.* 2018. **156**(1-2): p. 54-63.
36. Georgakis, M.K., et al., *Age at menopause and duration of reproductive period in association with dementia and cognitive function: A systematic review and meta-analysis.* Psychoneuroendocrinology, 2016. **73**: p. 224-243.
37. Tam, M. and E.J.S.i.c.e. Chui, *Ageing and learning: What do they mean to elders themselves?* 2016. **38**(2): p. 195-212.
38. Byrne, C., et al., *Ageing, Muscle Power and Physical Function: A Systematic Review and Implications for Pragmatic Training Interventions.* Sports Med, 2016. **46**(9): p. 1311-32.

39. Kobayashi, L.C., et al., *Aging and Functional Health Literacy: A Systematic Review and Meta-Analysis.* J Gerontol B Psychol Sci Soc Sci, 2016. **71**(3): p. 445-57.
40. Grande, S., *Aging, precarity, and the struggle for Indigenous elsewheres.* International Journal of Qualitative Studies in Education, 2018. **31**(3): p. 168-176.
41. Neves, H., et al., *AGITE-a medication self-management and adherence in the elderly questionnaire.* European Geriatric Medicine, 2017. **8**: p. S211.
42. DiBartolo, M.C. and J.M. Jarosinski, *Alcohol Use Disorder in Older Adults: Challenges in Assessment and Treatment.* Issues Ment Health Nurs, 2017. **38**(1): p. 25-32.
43. Luis-Ferreira, F., et al., *An Architecture to Support Wearables in Education and Wellbeing.* 2017.
44. Wilson, J., et al., *Oral Presentations.* Global Spine Journal, 2017. **7**(2_suppl): p. 2S-189S.
45. N.J., C., et al., *Anorexia in older people and its treatment: A systematic review.* 2019. **48**(Supplement_1): p. i26-i26.
46. Balasubramaniam, M., et al., *Antidepressants for anxiety disorders in late-life: A systematic review.* Ann Clin Psychiatry, 2019. **31**(4): p. 277-291.
47. Tampi, R.R., D.J. Tampi, and S.J.C.T.O.i.P. Balachandran, *Antipsychotics, antidepressants, anticonvulsants, melatonin, and benzodiazepines for behavioral and psychological symptoms of dementia: a systematic review of meta-analyses.* 2017. **4**(1): p. 55-79.
48. Gu, Y. and S.M. Dennis, *Are falls prevention programs effective at reducing the risk factors for falls in people with type-2 diabetes mellitus and peripheral neuropathy: A systematic review with narrative synthesis.* Journal of Diabetes and Its Complications, 2017. **31**(2): p. 504-516.
49. Burton, E., et al., *Are interventions effective in improving the ability of older adults to rise from the floor independently? A mixed method systematic review.* Disabil Rehabil, 2020. **42**(6): p. 743-753.
50. Kelley, M., *Assessing prevention strategies for Alzheimer's dementia: A new approach.* Alzheimer's & Dementia, 2017. **13**(7S_Part_25): p. P1213-P1213.
51. Cox, N.J., et al., *Assessment and Treatment of the Anorexia of Aging: A Systematic Review.* Nutrients, 2019. **11**(1): p. 144.
52. Jenabi, E., et al., *Assessment of Questionnaire Measuring Quality of Life in Menopausal Women: A Systematic Review.* Oman Med J, 2015. **30**(3): p. 151-6.
53. Edwards, D., J. Carrier, and J. Hopkinson, *Assistance at mealtimes in hospital settings and rehabilitation units for patients (>65years) from the perspective of patients, families and healthcare professionals: A mixed methods systematic review.* Int J Nurs Stud, 2017. **69**: p. 100-118.
54. Van der Roest, H.G., et al., *Assistive technology for memory support in dementia.* Cochrane Database Syst Rev, 2017. **6**(6): p. CD009627.
55. Wang, R., et al., *Association Between Cerebral Microbleeds and Depression in the General Elderly Population: A Meta-Analysis.* Front Psychiatry, 2018. **9**:

p. 94.
56. Kojima, G., et al., *Association between frailty and quality of life among community-dwelling older people: a systematic review and meta-analysis.* J Epidemiol Community Health, 2016. **70**(7): p. 716-21.
57. Javed, A.A., et al., *Association Between Hormone Therapy and Muscle Mass in Postmenopausal Women: A Systematic Review and Meta-analysis.* JAMA Netw Open, 2019. **2**(8): p. e1910154.
58. Wang, W., Y. Sun, and D. Zhang, *Association Between Non-Steroidal Anti-Inflammatory Drug Use and Cognitive Decline: A Systematic Review and Meta-Analysis of Prospective Cohort Studies.* Drugs Aging, 2016. **33**(7): p. 501-9.
59. Niknejad, B., et al., *Association Between Psychological Interventions and Chronic Pain Outcomes in Older Adults A Systematic Review and Meta-analysis.* Jama Internal Medicine, 2018. **178**(6): p. 830-839.
60. Amirthalingam, H., et al., *Association between Sarcopenia and Osteoarthritis-Related Knee Structural Changes: A Systematic Review.* Osteoarthritis and Cartilage, 2019. **27**: p. S472-S472.
61. Zhou, J., et al., *Association between Stroke and Alzheimer's Disease: Systematic Review and Meta-Analysis.* Journal of Alzheimers Disease, 2015. **43**(2): p. 479-489.
62. Nicolaou, M., et al., *Association of a priori dietary patterns with depressive symptoms: a harmonised meta-analysis of observational studies.* Psychol Med, 2019: p. 1-12.
63. Du, Y., C. Oh, and J. No, *Associations between Sarcopenia and Metabolic Risk Factors: A Systematic Review and Meta-Analysis.* Journal of Obesity & Metabolic Syndrome, 2018. **27**(3): p. 175-185.
64. Cherko, M., L. Hickson, and M. Bhutta, *Auditory deprivation and health in the elderly.* Maturitas, 2016. **88**: p. 52-7.
65. Vseteckova, J., et al., *Barriers and facilitators to adherence to group exercise in institutionalized older people living with dementia: a systematic review.* Eur Rev Aging Phys Act, 2018. **15**(1): p. 11.
66. Predescu, I., et al., *Behavioral Activation Therapy for Older Adults with Depression: A Systematic Review of Effectiveness.* American Journal of Geriatric Psychiatry, 2018. **26**(3): p. S132-S132.
67. Strout, K.A., et al., *Behavioral interventions in six dimensions of wellness that protect the cognitive health of community-dwelling older adults: a systematic review.* 2016. **64**(5): p. 944-958.

68. Chen, L.-K.J.E.G., *Benefits and dynamics of learning gained through volunteering: A qualitative exploration guided by seniors' self-defined successful aging.* 2016. **42**(3): p. 220-230.
69. Weiss, J., et al., *Benefits and Harms of Treating Blood Pressure in Older Adults: A Systematic Review and Meta-analysis.* 2017.
70. Hirst, S.P., et al., *Best-Practice Guideline on the Prevention of Abuse and Neglect of Older Adults.* Can J Aging, 2016. **35**(2): p. 242-60.
71. Barnett, D.W., et al., *Built environmental correlates of older adults' total physical activity and walking: a systematic review and meta-analysis.* 2017. **14**(1): p. 103.
72. Learner, N.A. and J.M.J.P.T.R. Williams, *Can physical activity be used to maintain cognitive function in nursing home residents with dementia? A literature review.* 2016. **21**(3-6): p. 184-191.
73. Rizzoli, R. *CAN WE REDUCE FALLS AND FRACTURES?* in *OSTEOPOROSIS INTERNATIONAL*. 2017. SPRINGER LONDON LTD 236 GRAYS INN RD, 6TH FLOOR, LONDON WC1X 8HL, ENGLAND.
74. Gebara, M.A., et al., *Cause or Effect? Selective Serotonin Reuptake Inhibitors and Falls in Older Adults: A Systematic Review.* Am J Geriatr Psychiatry, 2015. **23**(10): p. 1016-28.
75. Quijoux, F., et al., *Center of pressure characteristics from quiet standing measures to predict the risk of falling in older adults: a protocol for a systematic review and meta-analysis.* Syst Rev, 2019. **8**(1): p. 232.
76. Haeger, A., et al., *Cerebral changes improved by physical activity during cognitive decline: A systematic review on MRI studies.* Neuroimage Clin, 2019. **23**: p. 101933.
77. Santos, F.H., *Challenges for the Healthy Ageing in People with Intellectual Disabilities.* Journal of Intellectual Disability Research, 2019. **63**(7): p. 650-650.
78. McCrae, C.S., et al., *Changes in sleep predict changes in affect in older caregivers of individuals with Alzheimer's dementia: a multilevel model approach.* 2016. **71**(3): p. 458-462.
79. Vlaeyen, E., et al., *Characteristics and effectiveness of fall prevention programs in nursing homes: a systematic review and meta-analysis of randomized controlled trials.* J Am Geriatr Soc, 2015. **63**(2): p. 211-21.
80. Joshi, P., N. Shah, and H. Kirane, *Characteristics of Older Adults in Medication-Assisted Treatment for Opioid Use Disorder: A Systematic Review.* American Journal on Addictions, 2019. **28**(3): p. 179-179.
81. Tanna, N.K., et al., *Clinical medication review as part of an elderly fallers patient care screening service.* 2015. **81**(1): p. 221-222.

82. Niermeyer, M.A., *Cognitive and gait decrements among non-demented older adults with Type 2 diabetes or hypertension: a systematic review.* Clinical Neuropsychologist, 2018. **32**(7): p. 1256-1281.
83. Nguyen, L., K. Murphy, and G. Andrews, *Cognitive and neural plasticity in old age: A systematic review of evidence from executive functions cognitive training.* Ageing Res Rev, 2019. **53**: p. 100912.
84. Mansor, N.S., et al., *Cognitive effects of video games in older adults and their moderators: a systematic review with meta-analysis and meta-regression.* 2019: p. 1-16.
85. Nishihira, J., T. Tokashiki, and Y. Ohya, *Cognitive Function and Calcium. Hypertension treatment in consideration of dementia.* Clin Calcium, 2015. **25**(2): p. 255-62.
86. Mewborn, C.M., C.A. Lindbergh, and L. Stephen Miller, *Cognitive Interventions for Cognitively Healthy, Mildly Impaired, and Mixed Samples of Older Adults: A Systematic Review and Meta-Analysis of Randomized-Controlled Trials.* Neuropsychol Rev, 2017. **27**(4): p. 403-439.
87. Yates, L.A., et al., *Cognitive leisure activities and future risk of cognitive impairment and dementia: systematic review and meta-analysis.* Int Psychogeriatr, 2016. **28**(11): p. 1791-1806.
88. Kallio, E.-L., et al., *Cognitive training interventions for patients with Alzheimer's disease: a systematic review.* 2017. **56**(4): p. 1349-1372.
89. Dham, P., et al., *Collaborative Care for Psychiatric Disorders in Older Adults: A Systematic Review.* Can J Psychiatry, 2017. **62**(11): p. 761-771.
90. Istvandity, L., *Combining music and reminiscence therapy interventions for wellbeing in elderly populations: A systematic review.* Complement Ther Clin Pract, 2017. **28**: p. 18-25.
91. Clarke, E.L., J.R. Evans, and L. Smeeth, *Community screening for visual impairment in older people.* Cochrane Database Syst Rev, 2018. **2**(2): p. CD001054.
92. Cheng, P., et al., *Comparative Effectiveness of Published Interventions for Elderly Fall Prevention: A Systematic Review and Network Meta-Analysis.* Int J Environ Res Public Health, 2018. **15**(3): p. 498.
93. Tsoi, K.K., et al., *Comparison of diagnostic performance of Two-Question Screen and 15 depression screening instruments for older adults: systematic review and meta-analysis.* Br J Psychiatry, 2017. **210**(4): p. 255-260.
94. Tricco, A.C., et al., *Comparisons of Interventions for Preventing Falls in Older Adults: A Systematic Review and Meta-analysis.* JAMA, 2017. **318**(17): p. 1687-1699.
95. Ellis, G., et al., *Comprehensive geriatric assessment for older adults admitted to hospital.* Cochrane Database Syst Rev, 2017. **9**(9): p. CD006211.

96. García-Casal, J.A., et al., *Computer-based cognitive interventions for people living with dementia: a systematic literature review and meta-analysis.* 2017. **21**(5): p. 454-467.
97. Yunus, R.M., N.N. Hairi, and W.Y. Choo, *Consequences of Elder Abuse and Neglect: A Systematic Review of Observational Studies.* Trauma Violence Abuse, 2019. **20**(2): p. 197-213.
98. Tvistholm, N., L. Munch, and A.K.J.J.o.C.N. Danielsen, *Constipation is casting a shadow over everyday life–a systematic review on older people's experience of living with constipation.* 2017. **26**(7-8): p. 902-914.
99. Brooke, J. and O.J.J.o.c.n. Ojo, *Contemporary views on dementia as witchcraft in sub-Saharan Africa: A systematic literature review.* 2020. **29**(1-2): p. 20-30.
100. Wilkinson, P. and Z. Izmeth, *Continuation and maintenance treatments for depression in older people.* Cochrane Database Syst Rev, 2016. **9**(9): p. CD006727.
101. Djerada, Z., et al., *Contribution of NO and impact of aging in flow mediated dilatation in healthy patients: meta-analysis and meta-regression.* Fundamental & Clinical Pharmacology, 2018. **32**: p. 87-87.
102. Gallego Galisteo, M., et al., *Controversies about insomnia therapy in elderly.* International Journal of Clinical Pharmacy, 2016. **38**(6): p. 583.
103. de Soárez, P.C., et al., *Cost-effectiveness analysis of universal vaccination of adults aged 60 years with 23-valent pneumococcal polysaccharide vaccine versus current practice in Brazil.* 2015. **10**(6): p. e0130217.
104. Bock, J.O., et al., *Cost-Effectiveness of the Treatment of Depression in Old Age.* Psychiatr Prax, 2015. **42**(5): p. 240-7.
105. Muelle, A. and M.J.C.A.R. Rodriguez, *Dance for people with alzheimer's disease: A mini-review.* 2019. **25**(1): p. 1-6.
106. Burks, H. and N. Rianon, *Defining quality of life measures in elderly populations with dementia: A Systematic Review.* Journal of the American Geriatrics Society, 2019. **67**: p. S173-S173.
107. Travers, J., et al., *Delaying and reversing frailty: a systematic review of primary care interventions.* Br J Gen Pract, 2019. **69**(678): p. e61-e69.
108. Snowden, M.B., et al., *Dementia and co-occurring chronic conditions: a systematic literature review to identify what is known and where are the gaps in the evidence?* 2017. **32**(4): p. 357-371.
109. Scott, J., et al., *Depression and anxiety after total joint replacement among older adults: a meta-analysis.* 2016. **20**(12): p. 1243-1254.
110. Vaughan, L., A.L. Corbin, and J.S. Goveas, *Depression and frailty in later life: a systematic review.* Clin Interv Aging, 2015. **10**: p. 1947-58.

111. Stubbs, B., et al., *Depression and Reduced Bone Mineral Density at the Hip and Lumbar Spine: A Comparative Meta-Analysis of Studies in Adults 60 Years and Older.* Psychosomatic Medicine, 2016. **78**(4): p. 492-500.
112. Hallock, H., et al., *Design of controls in trials of computerised cognitive training is ineffectual: A meta-analysis in healthy older adults.* Alzheimer's and Dementia, 2017. **13**(7): p. P526.
113. Small, G.W., *Detection and Prevention of Cognitive Decline.* Am J Geriatr Psychiatry, 2016. **24**(12): p. 1142-1150.
114. Jongstra, S., et al., *Development and Validation of an Interactive Internet Platform for Older People: The Healthy Ageing Through Internet Counselling in the Elderly Study.* Telemedicine and E-Health, 2017. **23**(2): p. 96-104.
115. Åhsberg, E., et al., *Development of an instrument for assessing elder care needs.* 2017. **27**(3): p. 291-306.
116. Zeng, H., et al., *Dexmedetomidine for the prevention of postoperative delirium in elderly patients undergoing noncardiac surgery: A meta-analysis of randomized controlled trials.* PLoS One, 2019. **14**(8): p. e0218088.
117. Valencia, W.M., et al., *Diabetes Treatment in the Elderly: Incorporating Geriatrics, Technology, and Functional Medicine.* Curr Diab Rep, 2018. **18**(10): p. 95.
118. Bloom, I., et al., *Diet Quality and Sarcopenia in Older Adults: A Systematic Review.* Nutrients, 2018. **10**(3): p. 308.
119. Govindaraju, T., et al., *Dietary Patterns and Quality of Life in Older Adults: A Systematic Review.* Nutrients, 2018. **10**(8): p. 971.
120. Milte, C.M. and S.A. McNaughton, *Dietary patterns and successful ageing: a systematic review.* Eur J Nutr, 2016. **55**(2): p. 423-450.
121. Stockwell, S., et al., *Digital behavior change interventions to promote physical activity and/or reduce sedentary behavior in older adults: A systematic review and meta-analysis.* 2019. **120**: p. 68-87.
122. Dong, Y., et al., *Do low-serum vitamin E levels increase the risk of Alzheimer disease in older people? Evidence from a meta-analysis of case-control studies.* 2018. **33**(2): p. e257-e263.
123. Neri, S.G., et al., *Do virtual reality games improve mobility skills and balance measurements in community-dwelling older adults? Systematic review and meta-analysis.* Clin Rehabil, 2017. **31**(10): p. 1292-1304.
124. Guillamón, E.M.-C., et al., *Does aquatic exercise improve commonly reported predisposing risk factors to falls within the elderly? A systematic review.* 2019. **19**(1): p. 52.
125. Zhuori, N., et al., *Does Social Support Affect the Health of the Elderly in Rural China? A Meta-Analysis Approach.* 2019. **16**(18): p. 3471.

126. Borgi, M., et al., *Dog Visiting Programs for Managing Depressive Symptoms in Older Adults: A Meta-Analysis.* Gerontologist, 2020. **60**(1): p. e66-e75.
127. Bareham, B.K., et al., *Drinking in later life: a systematic review and thematic synthesis of qualitative studies exploring older people's perceptions and experiences.* 2019. **48**(1): p. 134-146.
128. Lussier, M., et al., *Early detection of mild cognitive impairment with in-home monitoring sensor technologies using functional measures: a systematic review.* 2018. **23**(2): p. 838-847.
129. Job, M., et al., *Ecological gait as a fall indicator in older adults: a systematic review.* 2019.
130. Dubas-Jakóbczyk, K., et al., *Economic evaluation of health promotion and primary prevention actions for older people—a systematic review.* 2017. **27**(4): p. 670-679.
131. Huter, K., et al., *Economic evaluation of health promotion interventions for older people: do applied economic studies meet the methodological challenges?* 2018. **16**(1): p. 1-11.
132. Flach, J., et al., *Economic potential of probiotic supplementation in institutionalized elderly with chronic constipation.* 2018. **6**(4): p. 198-206.
133. Lee, S.H. and H.S. Kim, *Effect of a multifactorial, interdisciplinary intervention on falls and fall rate of the older people in the community.* Value in Health, 2016. **19**(7): p. A400.
134. Schimmel, M., et al., *Effect of advanced age and/or systemic medical conditions on dental implant survival: A systematic review and meta-analysis.* 2018. **29**: p. 311-330.
135. Cheng, Y.-C., et al., *Effect of apolipoprotein E ε4 carrier status on cognitive response to acetylcholinesterase inhibitors in patients with Alzheimer's disease: a systematic review and meta-analysis.* 2018. **45**(5-6): p. 335-352.
136. Babakhanian, M., et al., *Effect of Aromatherapy on the Treatment of Psychological Symptoms in Postmenopausal and Elderly Women: A Systematic Review and Meta-analysis.* J Menopausal Med, 2018. **24**(2): p. 127-132.
137. Chilibeck, P.D., et al., *Effect of creatine supplementation during resistance training on lean tissue mass and muscular strength in older adults: a meta-analysis.* Open Access J Sports Med, 2017. **8**: p. 213-226.
138. Barreira, E., et al., *Effect of food plans and training in glycemic and lipid profiles in elderly diabetic type 2: Systematic Review.* Revista Espanola de Nutricion Comunitaria, 2017. **23**: p. 40.
139. Khayya, A.J.J.o.E. and Practice, *Effect of Implementing a Discharge Plan on Functional Abilities of Geriatric Patients with Hip Fractures.* 2016. **7**(5): p. 42-

52.
140. Enette, L., et al., *Effect of Interval and Continuous Aerobic Training on Basal Serum and Plasma Brain-Derived Neurotrophic Factor Values in Seniors: A Systematic Review of Intervention Studies.* Rejuvenation Res, 2017. **20**(6): p. 473-483.
141. Roozbeh, N., et al., *Effect of Lavender on Sleep, Sexual Desire, Vasomotor, Psychological and Physical Symptom among Menopausal and Elderly Women: A Systematic Review.* 2019. **25**(2): p. 88-93.
142. D'Cunha, N.M., et al., *Effect of long-term nutraceutical and dietary supplement use on cognition in the elderly: A 10-year systematic review of randomised controlled trials.* 2018. **119**(3): p. 280-298.
143. Cuesta-Triana, F., et al., *Effect of Milk and Other Dairy Products on the Risk of Frailty, Sarcopenia, and Cognitive Performance Decline in the Elderly: A Systematic Review.* Adv Nutr, 2019. **10**(suppl_2): p. S105-S119.
144. Donaldson, A.I.C., et al., *Effect of nonmeat, high-protein supplementation on quality of life and clinical outcomes in older residents of care homes: a systematic review and meta-analysis.* Nutr Rev, 2019. **77**(2): p. 116-127.
145. Barker, A., J. Talevski, and M.-L.J.P. Bird, *Effect of pilates exercise for improving balance and decreasing falls risk in older adults: a systematic review with meta-analysis.* 2015. **101**: p. e111-e112.
146. Hu, Y.N., et al., *Effect of Tai Chi Exercise on Fall Prevention in Older Adults: Systematic Review and Meta-analysis of Randomized Controlled Trials.* International Journal of Gerontology, 2016. **10**(3): p. 131-136.
147. Mu, W.Q., et al., *Effect of Tai Chi for the prevention or treatment of osteoporosis in elderly adults: protocol for a systematic review and meta-analysis.* BMJ Open, 2018. **8**(4): p. e020123.
148. Aboutorabi, A., et al., *Effect of vibration on postural control and gait of elderly subjects: a systematic review.* Aging Clin Exp Res, 2018. **30**(7): p. 713-726.
149. Oliveira, J.S., et al., *Effect of interventions using physical activity trackers on physical activity in people aged 60 years and over: A systematic review and meta-analysis.* 2019: p. bjsports-2018-100324.
150. Alrushud, A.S., et al., *Effect of physical activity and dietary restriction interventions on weight loss and the musculoskeletal function of overweight and obese older adults with knee osteoarthritis: a systematic review and mixed method data synthesis.* BMJ Open, 2017. **7**(6): p. e014537.
151. Blom, J.W., et al., *Effectiveness and cost-effectiveness of proactive and multidisciplinary integrated care for older people with complex problems in general practice: an individual participant data meta-analysis.* Age and Ageing, 2018. **47**(5): p. 705-714.

152. Gall, D.J., Z. Jordan, and C.J.J.E.S. Stern, *Effectiveness and meaningfulness of art therapy as a tool for healthy aging: a comprehensive systematic review protocol.* 2015. **13**(3): p. 3-17.
153. Brims, L. and K. Oliver, *Effectiveness of assistive technology in improving the safety of people with dementia: a systematic review and meta-analysis.* Aging Ment Health, 2019. **23**(8): p. 942-951.
154. Marques, P., et al., *Effectiveness of bedrails in preventing falls among hospitalized older adults: a systematic review.* 2017. **15**(10): p. 2527-2554.
155. Muellmann, S., et al., *Effectiveness of eHealth interventions for the promotion of physical activity in older adults: A systematic review.* Prev Med, 2018. **108**: p. 93-110.
156. Villanueva, A.L., et al. *Effectiveness of family interventions in nursing homes. A systematic review*. in *Anales del sistema sanitario de Navarra*. 2015.
157. Morilla-Herrera, J.C., et al., *Effectiveness of Food-Based Fortification in Older People. A Systematic Review and Meta-Analysis.* J Nutr Health Aging, 2016. **20**(2): p. 178-84.
158. Pritchard, C., et al., *Effectiveness of hospital avoidance interventions among elderly patients: A systematic review.* 2020: p. 1-10.
159. Robalino, S., et al., *Effectiveness of interventions aimed at improving physical and psychological outcomes of fall-related injuries in people with dementia: a narrative systematic review.* Systematic Reviews, 2018. **7**(1): p. 31.
160. Logan, A., et al., *Effectiveness of non-pharmacological interventions in treating orthostatic hypotension in the elderly and people with a neurological condition: a systematic review protocol.* JBI Database System Rev Implement Rep, 2017. **15**(4): p. 948-960.
161. Strupeit, S., A. Buss, and T. Dassen, *Effectiveness of nurse-delivered patient education interventions on quality of life in elders in the hospital: A systematic review.* Appl Nurs Res, 2016. **32**: p. 217-221.
162. Mabire, C., et al., *Effectiveness of nursing discharge planning interventions on health-related outcomes in discharged elderly inpatients: a systematic review.* JBI Database System Rev Implement Rep, 2016. **14**(9): p. 217-260.
163. Poscia, A., et al., *Effectiveness of nutritional interventions addressed to elderly persons: umbrella systematic review with meta-analysis.* Eur J Public Health, 2018. **28**(2): p. 275-283.
164. Cuevas-Lara, C., et al., *Effectiveness of occupational therapy interventions in acute geriatric wards: A systematic review.* Maturitas, 2019. **127**: p. 43-50.
165. Vernaya, M., J. McAdam, and M.D. Hampton, *Effectiveness of probiotics in reducing the incidence of Clostridium difficile-associated diarrhea in elderly patients: a systematic review.* JBI Database System Rev Implement Rep, 2017. **15**(1): p. 140-164.

166. Apostolo, J., et al., *Effectiveness of the interventions in preventing the progression of pre-frailty and frailty in older adults: a systematic review protocol.* JBI Database System Rev Implement Rep, 2016. **14**(1): p. 4-19.
167. Briones-Peralta, M. and B.J.R.e.d.g.y.g. Rodríguez-Martín, *Effectiveness of training interventions aimed at reducing physical restraints in institutionalised older people: A systematic review.* 2017. **52**(2): p. 93-101.
168. Rasmussen, N.M.L., et al., *Effectiveness of multidisciplinary nutritional support in older hospitalised patients: A systematic review and meta-analyses.* Clin Nutr ESPEN, 2018. **27**: p. 44-52.
169. Burton, E., et al., *Effectiveness of peers in delivering programs or motivating older people to increase their participation in physical activity: Systematic review and meta-analysis.* Journal of Sports Sciences, 2018. **36**(6): p. 666-678.
170. Roets-Merken, L.M., et al., *Effectiveness of rehabilitation interventions in improving emotional and functional status in hearing or visually impaired older adults: a systematic review with meta-analyses.* Clin Rehabil, 2015. **29**(2): p. 107-19.
171. Renz, J.C. and M. Meinck, *Effectiveness of preventive house visits for elderly people : Systematic assessment of current literature.* Z Gerontol Geriatr, 2018. **51**(8): p. 924-930.
172. Borhan, A.S.M., et al., *Effects of dance on cognitive function among older adults: a protocol for systematic review and meta-analysis.* Systematic Reviews, 2018. **7**(1): p. 24.
173. Meng, X., et al., *Effects of dance intervention on global cognition, executive function and memory of older adults: a meta-analysis and systematic review.* Aging Clin Exp Res, 2020. **32**(1): p. 7-19.
174. Rieping, T., et al., *Effects of Different Chair-Based Exercises on Salivary Biomarkers and Functional Autonomy in Institutionalized Older Women.* Res Q Exerc Sport, 2019. **90**(1): p. 36-45.
175. Zhou, X.L., et al., *Effects of exercise interventions for specific cognitive domains in old adults with mild cognitive impairment: A protocol of subgroup meta-analysis of randomized controlled trials.* Medicine (Baltimore), 2018. **97**(48): p. e13244.
176. van Santen, J., et al., *Effects of Exergaming in People with Dementia: Results of a Systematic Literature Review.* J Alzheimers Dis, 2018. **63**(2): p. 741-760.
177. Chan, J.S., et al., *Effects of meditation and mind–body exercises on older adults' cognitive performance: a meta-analysis.* 2019. **59**(6): p. e782-e790.
178. Matia-Martin, P., et al., *Effects of Milk and Dairy Products on the Prevention of Osteoporosis and Osteoporotic Fractures in Europeans and Non-Hispanic Whites from North America: A Systematic Review and Updated Meta-Analysis.* Advances in Nutrition, 2019. **10**(suppl_2): p. S120-S143.

179. Hidayat, K., et al., *Effects of Milk Proteins Supplementation in Older Adults Undergoing Resistance Training: A Meta-Analysis of Randomized Control Trials.* J Nutr Health Aging, 2018. **22**(2): p. 237-245.
180. Wu, C., et al., *Effects of Mind-Body Exercises on Cognitive Function in Older Adults: A Meta-Analysis.* J Am Geriatr Soc, 2019. **67**(4): p. 749-758.
181. Dedeyne, L., et al., *Effects of multi-domain interventions in (pre)frail elderly on frailty, functional, and cognitive status: a systematic review.* Clin Interv Aging, 2017. **12**: p. 873-896.
182. Seo, K. and H.N. Kim, *Effects of oral health programmes on xerostomia in community-dwelling elderly: A systematic review and meta-analysis.* Int J Dent Hyg, 2020. **18**(1): p. 52-61.
183. Valdes-Badilla, P.A., et al., *Effects of Physical Activity Governmental Programs on Health Status in Independent Older Adults: A Systematic Review.* Journal of Aging and Physical Activity, 2019. **27**(2): p. 265-275.
184. Pedroso, R.V., et al., *Effects of physical activity on the P 300 component in elderly people: a systematic review.* 2017. **17**(6): p. 479-487.
185. Dahm, K.T., et al., *Effects of preventive use of compression stockings for elderly with chronic venous insufficiency and swollen legs: a systematic review and meta-analysis.* Bmc Geriatrics, 2019. **19**(1): p. 76.
186. Liao, C.D., et al., *Effects of Protein Supplementation Combined with Exercise Intervention on Frailty Indices, Body Composition, and Physical Function in Frail Older Adults.* Nutrients, 2018. **10**(12): p. 1916.
187. Liao, C.D., et al., *Effects of protein supplementation combined with resistance exercise on body composition and physical function in older adults: a systematic review and meta-analysis.* Am J Clin Nutr, 2017. **106**(4): p. 1078-1091.
188. Klainin-Yobas, P., et al., *Effects of relaxation interventions on depression and anxiety among older adults: a systematic review.* Aging & Mental Health, 2015. **19**(12): p. 1043-1055.
189. da Rosa Orssatto, L.B., et al., *Effects of resistance training concentric velocity on older adults' functional capacity: A systematic review and meta-analysis of randomised trials.* Exp Gerontol, 2019. **127**: p. 110731.
190. Hall, J., et al., *Efficacy of Cognitive Behavioral Therapy for Generalized Anxiety Disorder in Older Adults: Systematic Review, Meta-Analysis, and Meta-Regression.* Am J Geriatr Psychiatry, 2016. **24**(11): p. 1063-1073.
191. Tavares, L.R. and M.R. Barbosa, *Efficacy of group psychotherapy for geriatric depression: A systematic review.* Arch Gerontol Geriatr, 2018. **78**: p. 71-80.
192. Zhang, D.M., et al., *Efficacy of Vitamin B Supplementation on Cognition in Elderly Patients With Cognitive-Related Diseases: A Systematic Review and*

Meta-Analysis. Journal of Geriatric Psychiatry and Neurology, 2017. **30**(1): p. 50-59.
193. Krause, M., et al., *Efficacy and tolerability of pharmacological and non-pharmacological interventions in older patients with major depressive disorder: A systematic review, pairwise and network meta-analysis.* Eur Neuropsychopharmacol, 2019. **29**(9): p. 1003-1022.
194. Vazquez, F.L., et al., *Efficacy of video game-based interventions for active aging. A systematic literature review and meta-analysis.* Plos One, 2018. **13**(12): p. e0208192.
195. Colombo, D., et al., *Egocentric and allocentric spatial reference frames in aging: A systematic review.* Neurosci Biobehav Rev, 2017. **80**: p. 605-621.
196. O'quin, K., T. Semalulu, and H.J.H.E.R. Orom, *Elder and caregiver solutions to improve medication adherence.* 2015. **30**(2): p. 323-335.
197. Liang, S., et al., *Electroconvulsive treatment for geriatric depression: a systematic review of randomized clinical trials.* 2018. **38**(1): p. 76.
198. Shah, T.M., et al., *Enhancing Cognitive Functioning in Healthly Older Adults: a Systematic Review of the Clinical Significance of Commercially Available Computerized Cognitive Training in Preventing Cognitive Decline.* Neuropsychol Rev, 2017. **27**(1): p. 62-80.
199. Herke, M., et al., *Environmental and behavioural modifications for improving food and fluid intake in people with dementia.* Cochrane Database Syst Rev, 2018. **7**(7): p. CD011542.
200. Wu, Y., H.V. MacDonald, and L.S. Pescatello, *Evaluating Exercise Prescription and Instructional Methods Used in Tai Chi Studies Aimed at Improving Balance in Older Adults: A Systematic Review.* Journal of the American Geriatrics Society, 2016. **64**(10): p. 2074-2080.
201. Ward, M.C., et al., *Evaluating the cost of endocrine therapy vs. radiation therapy alone for low risk hormone positive early stage breast cancer in elderly patients.* Cancer Research, 2019. **79**(4).
202. Matsuzawa, R., et al., *Evaluating the effectiveness of exercise training on elderly patients who require haemodialysis: study protocol for a systematic review and meta-analysis.* BMJ Open, 2016. **6**(5): p. e010990.
203. Boekhout, J.M., et al., *Evaluation of a Computer-Tailored Healthy Ageing Intervention to Promote Physical Activity among Single Older Adults with a Chronic Disease.* International Journal of Environmental Research and Public Health, 2018. **15**(2): p. 346.
204. Kim, K.I., et al., *Evidence-based guidelines for fall prevention in Korea.* Korean J Intern Med, 2017. **32**(1): p. 199-210.
205. Custodero, C., et al., *Evidence-based nutritional and pharmacological interventions targeting chronic low-grade inflammation in middle-age and older adults: A systematic review and meta-analysis.* Ageing Res Rev, 2018. **46**: p. 42-59.
206. Gomes-Osman, J., et al., *Exercise for cognitive brain health in aging: A systematic review for an evaluation of dose.* Neurol Clin Pract, 2018. **8**(3): p. 257-

265.
207. Sherrington, C., et al., *Exercise to prevent falls in older adults: an updated systematic review and meta-analysis.* Br J Sports Med, 2017. **51**(24): p. 1750-1758.
208. de Souto Barreto, P., et al., *Exercise Training for Preventing Dementia, Mild Cognitive Impairment, and Clinically Meaningful Cognitive Decline: A Systematic Review and Meta-analysis.* J Gerontol A Biol Sci Med Sci, 2018. **73**(11): p. 1504-1511.
209. Northey, J.M., et al., *Exercise interventions for cognitive function in adults older than 50: a systematic review with meta-analysis.* British Journal of Sports Medicine, 2018. **52**(3): p. 154-+.
210. Klimova, B., M. Valis, and K. Kuca, *exploring assistive technology as a potential beneficial intervention tool for people with Alzheimer's disease–a systematic review.* Neuropsychiatric disease and treatment, 2018. **14**: p. 3151.
211. Porter, J., et al., *Extending international access to doubly-labelled water data in older adults.* Proceedings of the Nutrition Society, 2018. **77**(Oce4): p. E170-E170.
212. Denkinger, M.D., et al., *Factors associated with fear of falling and associated activity restriction in community-dwelling older adults: a systematic review.* Am J Geriatr Psychiatry, 2015. **23**(1): p. 72-86.
213. Woo, Y.-S., et al., *Factors Influencing Driving Ability and Its Measurements in Older Drivers: A Systematic Review.* Alzheimer's & Dementia, 2018. **14**(7S_Part_25): p. P1363-P1364.
214. Bruns, M.P., H. Reich, and T. Chau, *Fall Prevention Visit in a Residency Primary Care Clinic.* Journal of the American Geriatrics Society, 2019. **67**: p. S135-S135.
215. Lapteva, E.S., et al., *Falls in the elderly and senior age prevention perspectives.* Adv Gerontol, 2019. **32**(3): p. 469-476.
216. Armenta, B.M., et al., *Feeling younger and identifying with older adults: Testing two routes to maintaining well-being in the face of age discrimination.* PLoS One, 2017. **12**(11): p. e0187805.
217. Samieri, C., et al., *Fish intake, Alzheimer disease genes and cognitive decline in five large cohorts of older subjects: 149/697.* Annals of Nutrition and Metabolism, 2015. **67**.
218. Alberto Aguilera-Eguia, R., et al., *Folic acid supplementation with or without vitamin B-12 to prevent cognitive decline in elderly subjects: critical analysis of literature.* REVISTA ESPANOLA DE NUTRICION HUMANA Y DIETETICA, 2015. **19**(4): p. 231-237.

219. Ginis, K.A.M., et al., *Formulation of evidence-based messages to promote the use of physical activity to prevent and manage Alzheimer's disease.* BMC Public Health, 2017. **17**(1): p. 209.
220. Dauenhauer, J., D.W. Steitz, and L.J. Cochran, *Fostering a new model of multigenerational learning: Older adult perspectives, community partners, and higher education.* Educational Gerontology, 2016. **42**(7): p. 483-496.
221. Cheng, M.H. and S.F. Chang, *Frailty as a risk factor for falls among community dwelling people: Evidence from a meta-analysis.* Journal of nursing scholarship, 2017. **49**(5): p. 529-536.
222. Kallenberg, M.H., et al., *Functional and cognitive impairment, frailty, and adverse health outcomes in older patients reaching ESRD—a systematic review.* Clinical Journal of the American Society of Nephrology, 2016. **11**(9): p. 1624-1639.
223. Duda, B.M. and L.H. Sweet, *Functional brain changes associated with cognitive training in healthy older adults: A preliminary ALE meta-analysis.* Brain Imaging Behav, 2019: p. 1-16.
224. Torres-de Araújo, J.R., et al., *Functional, nutritional and social factors associated with mobility limitations in the elderly: a systematic review.* salud pública de méxico, 2019. **60**: p. 579-585.
225. Greenwood, N., F. Pelone, and A.M. Hassenkamp, *General practice based psychosocial interventions for supporting carers of people with dementia or stroke: a systematic review.* BMC Fam Pract, 2016. **17**(1): p. 3.
226. van der Mark-Reeuwijk, K.G., et al., *Health and prolonging working lives: an advisory report of the Health Council of The Netherlands.* Scand J Work Environ Health, 2019. **45**(5): p. 514-519.
227. Bouaziz, W., et al., *Health benefits of aerobic training programs in adults aged 70 and over: a systematic review.* Archives of gerontology and geriatrics, 2017. **69**: p. 110-127.
228. Bouaziz, W., et al., *Health benefits of multicomponent training programmes in seniors: a systematic review.* Int J Clin Pract, 2016. **70**(7): p. 520-36.
229. Guo, Y., et al., *Health benefits of traditional Chinese sports and physical activity for older adults: A systematic review of evidence.* J Sport Health Sci, 2016. **5**(3): p. 270-280.
230. Courel-Ibáñez, J., et al., *Health Benefits of β-Hydroxy-β-Methylbutyrate (HMB) Supplementation in Addition to Physical Exercise in Older Adults: A Systematic Review with Meta-Analysis.* Nutrients, 2019. **11**(9): p. 2082.
231. Ryman, F.V.M., et al., *Health Effects of the Relocation of Patients With Dementia: A Scoping Review to Inform Medical and Policy Decision-Making.*

Gerontologist, 2019. **59**(6): p. e674-e682.
232. Estrela, A.L. and M.E. Bauer, *Healthy aging and physical activity: a systematic review on the effects of exercise on cardiovascular diseases.* Scientia Medica, 2017. **27**(1): p. 147-154.
233. Smith, G.E., *Healthy cognitive aging and dementia prevention.* Am Psychol, 2016. **71**(4): p. 268-75.
234. Rubtsova, A.A., et al., *Healthy Aging in Older Women Living with HIV Infection: a Systematic Review of Psychosocial Factors.* Curr HIV/AIDS Rep, 2017. **14**(1): p. 17-30.
235. Brown, L., et al., *Heart rate variability alterations in late life depression: A meta-analysis.* J Affect Disord, 2018. **235**: p. 456-466.
236. Fraga Dominguez, S., J.E. Storey, and E. Glorney, *Help-Seeking Behavior in Victims of Elder Abuse: A Systematic Review.* Trauma Violence Abuse, 2019: p. 1524838019860616.
237. Kwon, C.Y., et al., *Herbal medicine for insomnia in elderly with hypertension: A systematic review and meta-analysis.* European Journal of Integrative Medicine, 2019. **30**.
238. Mas, M.A. and S. Santaeugenia, *Hospital-at-home in older patients: a scoping review on opportunities of developing comprehensive geriatric assessment based services.* Rev Esp Geriatr Gerontol, 2015. **50**(1): p. 26-34.
239. Feter, N., et al., *How do different physical exercise parameters modulate brain-derived neurotrophic factor in healthy and non-healthy adults? A systematic review, meta-analysis and meta-regression.* Science & Sports, 2019. **34**(5): p. 293-304.
240. Vandemeulebroucke, T., B.D. de Casterle, and C. Gastmans, *How do older adults experience and perceive socially assistive robots in aged care: a systematic review of qualitative evidence.* Aging & Mental Health, 2018. **22**(2): p. 149-167.
241. Wurm, S., et al., *How do views on aging affect health outcomes in adulthood and late life? Explanations for an established connection.* Developmental Review, 2017. **46**: p. 27-43.
242. Cai, Y. and K. Abrahamson, *How exercise influences cognitive performance when mild cognitive impairment exists: A literature review.* Journal of psychosocial nursing and mental health services, 2015. **54**(1): p. 25-35.
243. Schepens, H.R.M.M., J. Van Puyenbroeck, and B. Maes, *How to improve the quality of life of elderly people with intellectual disability: A systematic literature review of support strategies.* Journal of Applied Research in Intellectual Disabilities, 2019. **32**(3): p. 483-521.
244. Pinto-Bruno, A.C., et al., *ICT-based applications to improve social health and social participation in older adults with dementia. A systematic literature*

review. Aging Ment Health, 2017. **21**(1): p. 58-65.
245. McGowan, L., et al., *Impact of behavioural dietary intervention coupled with oral rehabilitation on the health and nutritional status of older adults: A systematic review and meta-analysis.* Obesity Facts, 2019. **12**: p. 127.
246. Lehne, G. and G. Bolte, *Impact of universal interventions on social inequalities in physical activity among older adults: an equity-focused systematic review.* International Journal of Behavioral Nutrition and Physical Activity, 2017. **14**(1): p. 20.
247. Vecchio, M.G., C. Minto, and D. Gregori, *Impact of Vitamin C supplementation on Risk for Cardiovascular events: future scenario for the elderly US population.* Faseb Journal, 2016. **30**(1_supplement): p. 1172.3-1172.3.
248. Farhang, M., et al., *Impact of mind-body interventions in older adults with mild cognitive impairment: a systematic review.* Int Psychogeriatr, 2019. **31**(5): p. 643-666.
249. Folbert, E.C., et al., *Improved 1-year mortality in elderly patients with a hip fracture following integrated orthogeriatric treatment.* Osteoporos Int, 2017. **28**(1): p. 269-277.
250. Harrold, S.A., et al., *Increasing physical activity for veterans in the Mental Health Intensive Case Management Program: A community-based intervention.* Perspect Psychiatr Care, 2018. **54**(2): p. 266-273.
251. Geritz, J., W. Maetzler, and C. Schlenstedt, *Influence of computer-based cognitive training on mobility in healthy older adults : A systematic review.* Z Gerontol Geriatr, 2018. **51**(2): p. 184-192.
252. Thomas, R.E., T. Jefferson, and T.J. Lasserson, *Influenza vaccination for healthcare workers who care for people aged 60 or older living in long-term care institutions.* Cochrane Database Syst Rev, 2016(6): p. CD005187.
253. de Almondes, K.M., et al., *Insomnia and risk of dementia in older adults: Systematic review and meta-analysis.* J Psychiatr Res, 2016. **77**: p. 109-15.
254. Kim, S.J. and G.E. Yoo, *Instrument Playing as a Cognitive Intervention Task for Older Adults: A Systematic Review and Meta-Analysis.* Frontiers in Psychology, 2019. **10**: p. 151.
255. Bruce, M.L. and J.A. Sirey, *Integrated Care for Depression in Older Primary Care Patients.* Can J Psychiatry, 2018. **63**(7): p. 439-446.
256. Hopwood, J., et al., *Internet-Based Interventions Aimed at Supporting Family Caregivers of People With Dementia: Systematic Review.* Journal of Medical Internet Research, 2018. **20**(6): p. e216.
257. González-Román, L., et al., *Interventions based on exercise and physical environment for preventing falls in cognitively impaired older people living in*

long-term care facilities: A systematic review and meta-analysis. Revista Espanola de Geriatria y Gerontologia, 2016. **51**(2): p. 96-111.

258. Anthony, K., et al., *Interventions designed to increase older people's exercise engagement: a systematic review.* Physiotherapy, 2016. **102**: p. e232.
259. Petersen, R.C., *Interventions for preventing cognitive decline and dementia: Recommendations from a national academies of sciences, engineering, and medicine study.* Alzheimer's and Dementia, 2017. **13**(7): p. P1214.
260. Vrkljan, B., et al., *Interventions that support major life transitions in older adulthood: a systematic review.* International Psychogeriatrics, 2019. **31**(3): p. 393-415.
261. Golding-Day, M., et al., *Interventions to reduce dependency in bathing in community dwelling older adults: a systematic review.* Syst Rev, 2017. **6**(1): p. 198.
262. Baker, P.R., et al., *Interventions for preventing elder abuse: applying findings of a new Cochrane review.* Age Ageing, 2017. **46**(3): p. 346-348.
263. Poscia, A., et al., *Interventions targeting loneliness and social isolation among the older people: An update systematic review.* Experimental Gerontology, 2018. **102**: p. 133-144.
264. Alldred, D.P., et al., *Interventions to optimise prescribing for older people in care homes.* Cochrane Database Syst Rev, 2016. **2**(2): p. CD009095.
265. Valencia, M.G., et al., *Interventions to optimize pharmacologic treatment in hospitalized older adults: a systematic review.* Revista Clinica Espanola, 2016. **216**(4): p. 205-221.
266. Barnett, J., et al., *Interventions to prevent cognitive decline and dementia in adults without cognitive impairment: a systematic review.* J Prev Alzheimers Dis, 2015. **2**: p. 38-45.
267. Inglis, S.C., et al., *Is age a factor in the success or failure of remote monitoring in heart failure? Telemonitoring and structured telephone support in elderly heart failure patients.* European Journal of Cardiovascular Nursing, 2015. **14**(3): p. 248-255.
268. Sulaiman, M.S., F. Nizamuddin, and T.B. Sunny, *Is exercise therapy effective in the older people? A systematic review.* Physiotherapy, 2015. **101**: p. e1455.
269. Scheel, F. and M. Carrasco, *Is vitamin D supplementation effective for the prevention of falls in elderly people?* Medwave, 2016. **16**(Suppl 1).
270. Bischoff-Ferrari, H.A., B. Dawson-Hughes, and W.C. Willett, *Issues of trial selection and subgroup considerations in the recent meta-analysis of Zhao and colleagues on fracture reduction by calcium and vitamin D supplementation in community-dwelling older adults.* Osteoporos Int, 2018. **29**(9): p. 2151-2152.
271. Fissler, P., et al., *Jigsaw Puzzles As Cognitive Enrichment (PACE) - the effect of solving jigsaw puzzles on global visuospatial cognition in adults 50 years of*

age and older: study protocol for a randomized controlled trial. Trials, 2017. **18**(1): p. 415.
272. Cosco, T.D., et al., *Latent structure of the Centre for Epidemiologic Studies Depression Scale (CES-D) in older adult populations: a systematic review.* Aging Ment Health, 2020. **24**(5): p. 700-704.
273. Tam, M., *Later life learning experiences: listening to the voices of Chinese elders in Hong Kong.* International Journal of Lifelong Education, 2016. **35**(5): p. 569-585.
274. Serrat, R., et al., *Learning Through Political Participation: A Case Study of Spanish Elders Involved in Political Organizations.* Adult Education Quarterly, 2016. **66**(2): p. 169-187.
275. Mollers, T., et al., *Length of hospital stay and dementia: A systematic review of observational studies.* International Journal of Geriatric Psychiatry, 2019. **34**(1): p. 8-21.
276. Elfrink, T.R., et al., *Life story books for people with dementia: a systematic review.* Int Psychogeriatr, 2018. **30**(12): p. 1797-1811.
277. Li, A.T. and H.C. Wei, *Lifelong Learning for Aging People in Taiwan: Innovative Programs and Social Effects.* New Directions for Adult and Continuing Education, 2019. **2019**(162): p. 97-110.
278. Zhao, X., et al., *Light therapy for older patients with non-seasonal depression: A systematic review and meta-analysis.* J Affect Disord, 2018. **232**: p. 291-299.
279. Malcolm, M., H. Frost, and J. Cowie, *Loneliness and social isolation causal association with health-related lifestyle risk in older adults: a systematic review and meta-analysis protocol.* Syst Rev, 2019. **8**(1): p. 48.
280. Campbell, A., et al., *Long-Term Aerobic Exercise Improves Vascular Function Into Old Age: A Systematic Review, Meta-Analysis and Meta Regression of Observational and Interventional Studies.* Frontiers in Physiology, 2019. **10**: p. 31.
281. Sanchez-Riera, L., et al., *Low bone mineral density is the main contributor to falls-related health burden in the elderly.* 2017.
282. Corish, C.A. and L.A. Bardon, *Malnutrition in older adults: screening and determinants.* Proc Nutr Soc, 2019. **78**(3): p. 372-379.
283. Negm, A.M., et al., *Management of Frailty: A Systematic Review and Network Meta-analysis of Randomized Controlled Trials.* J Am Med Dir Assoc, 2019. **20**(10): p. 1190-1198.
284. Falck, R.S., et al., *Measurement of physical activity in older adult interventions: a systematic review.* Br J Sports Med, 2016. **50**(8): p. 464-70.
285. Gabes, M., et al., *Measurement properties of patient-reported outcome measures (PROMs) for women with genitourinary syndrome of menopause: a*

systematic review. Menopause, 2019. **26**(11): p. 1342-1353.

286. Silva, R., et al., *Mediterranean Diet and Musculoskeletal-Functional Outcomes in Community-Dwelling Older People: A Systematic Review and Meta-Analysis.* J Nutr Health Aging, 2018. **22**(6): p. 655-663.

287. Blair, K., *'Memory in two voices:'an aesthetics of care in performance groups for older adults.* Research in Drama Education: The Journal of Applied Theatre and Performance, 2019. **24**(1): p. 67-71.

288. Jordao, M., et al., *Meta-Analysis of Aging Effects in Mind Wandering: Methodological and Sociodemographic Factors.* Psychology and Aging, 2019. **34**(4): p. 531-544.

289. Kirkham, J.G., N. Choi, and D.P. Seitz, *Meta-analysis of problem solving therapy for the treatment of major depressive disorder in older adults.* Int J Geriatr Psychiatry, 2016. **31**(5): p. 526-35.

290. Noone, D., et al., *Meta-analysis of psychosocial interventions for people with dementia and anxiety or depression.* Aging Ment Health, 2019. **23**(10): p. 1282-1291.

291. Creavin, S.T., et al., *Mini-Mental State Examination (MMSE) for the detection of dementia in clinically unevaluated people aged 65 and over in community and primary care populations.* Cochrane Database Syst Rev, 2016(1): p. CD011145.

292. Cornelis, E. *Mission accomplished? Active ageing in nursing homes: A new approach to enable meaningful activities and quality of life*. in *INTERNATIONAL PSYCHOGERIATRICS*. 2015. CAMBRIDGE UNIV PRESS 32 AVENUE OF THE AMERICAS, NEW YORK, NY 10013-2473 USA.

293. Elavsky, S., et al., *Mobile Health Interventions for Physical Activity, Sedentary Behavior, and Sleep in Adults Aged 50 Years and Older: A Systematic Literature Review.* J Aging Phys Act, 2019. **27**(4): p. 565-593.

294. Tahmosybayat, R., et al., *Movements of older adults during exergaming interventions that are associated with the Systems Framework for Postural Control: A systematic review.* Maturitas, 2018. **111**: p. 90-99.

295. Lugnet, K., et al., *Multidisciplinary nutritional intervention during hospitalization and after discharge in elderly patients-a meta-analysis.* Clinical Nutrition, 2016. **35**: p. S196.

296. Abbas, M., C. D'Sylva, and L.A. Fraser, *Multifactorial assessment and targeted intervention to prevent recurrent falls in community dwelling elderly individuals presenting to emergency departments: A systematic review and meta-analysis.* Endocrine Reviews, 2016. **37**(2).

297. Hopewell, S., et al., *Multifactorial interventions for preventing falls in older people living in the community: a systematic review and meta-analysis of 41*

trials and almost 20 000 participants. British journal of sports medicine, 2019: p. bjsports-2019-100732.
298. Stubbs, B., et al., *Multimorbidity and perceived stress: a population-based cross-sectional study among older adults across six low- and middle-income countries.* Maturitas, 2018. **107**: p. 84-91.
299. Roman-Caballero, R., et al., *Musical practice as an enhancer of cognitive function in healthy aging - A systematic review and meta-analysis.* Plos One, 2018. **13**(11): p. e0207957.
300. van der Steen, J.T., et al., *Music-based therapeutic interventions for people with dementia.* Cochrane Database Syst Rev, 2018. **7**(7): p. CD003477.
301. Walker, A.S., *Narrative Medicine: Community Poetry Heals Young and Old.* Community Literacy Journal, 2016. **11**(1): p. 138-145.
302. Zhang, Q., L.F. Young, and F. Li, *Network Meta-Analysis of Various Nonpharmacological Interventions on Pain Relief in Older Adults With Osteoarthritis.* American Journal of Physical Medicine & Rehabilitation, 2019. **98**(6): p. 469-478.
303. Glover, J.A., S. Hrisko, and S. Srinivasan, *New Ways to Count Sleep: A Review of Suvorexant for Insomnia in Older Adults.* American Journal of Geriatric Psychiatry, 2017. **25**(3): p. S146-S147.
304. Johnson, S., et al., *No Place Like Home: A Systematic Review of Home Care for Older Adults in Canada.* Canadian Journal on Aging-Revue Canadienne Du Vieillissement, 2018. **37**(4): p. 400-419.
305. Smart, C.M., et al., *Non-Pharmacologic Interventions for Older Adults with Subjective Cognitive Decline: Systematic Review, Meta-Analysis, and Preliminary Recommendations.* Neuropsychol Rev, 2017. **27**(3): p. 245-257.
306. Legere, L.E., et al., *Nonpharmacological approaches for behavioural and psychological symptoms of dementia in older adults: A systematic review of reviews.* J Clin Nurs, 2018. **27**(7-8): p. e1360-e1376.
307. Herrod, P.J.J., et al., *Non-pharmacological strategies to reduce blood pressure in older adults: a systematic review and meta-analysis.* Lancet, 2017. **390**: p. S43-S43.
308. Fong, S.S.M., et al., *Novel aquatic physiotherapy programme for elderly Chinese adults with osteoarthritis of the knee: a randomised controlled trial.* Lancet, 2017. **390**: p. S33-S33.
309. Hsieh, P.L. and C.M. Chen, *[Nurse-Led Care Models in the Context of Community Elders With Chronic Disease Management: A Systematic Review].* Hu Li Za Zhi, 2016. **63**(4): p. 35-49.
310. Olagunju, A., et al., *Nutrition and Bipolar Disorders in Older Adults: A systematic Review.* The American Journal of Geriatric Psychiatry, 2019. **27**(3): p.

S207-S208.
311. Saleh, R.A., et al. *Nutrition, or Nutrition and Exercise? A Systematic Review of Interventions in Frail Elderly People*. in *JOURNAL OF THE AMERICAN GERIATRICS SOCIETY*. 2015. WILEY-BLACKWELL 111 RIVER ST, HOBOKEN 07030-5774, NJ USA.
312. Solfrizzi, V., et al., *Nutritional interventions and cognitive-related outcomes in patients with late-life cognitive disorders: A systematic review.* Neurosci Biobehav Rev, 2018. **95**: p. 480-498.
313. Boulos, C., et al., *Nutritional risk factors, microbiota and Parkinson's disease: what is the current evidence?* Nutrients, 2019. **11**(8): p. 1896.
314. Fernandez, S.S.M., T. Ivanauskas, and S.M.L. Ribeiro, *Nutritional Strategies in the Management of Alzheimer Disease: Systematic Review With Network Meta-Analysis.* Journal of the American Medical Directors Association, 2017. **18**(10): p. 897. e13-897. e30.
315. Chang, K.-C. and M.-J. Chou, *On the Life Aesthetics of Aging People--Slowness Makes Life Beautiful.* Universal Journal of Educational Research, 2015. **3**(12): p. 967-972.
316. Nangle, M.R., et al., *Oral Health and Cognitive Function in Older Adults: A Systematic Review.* Gerontology, 2019. **65**(6): p. 659-672.
317. Wright, J. and C. Baldwin, *Oral nutritional support with or without exercise in the management of malnutrition in nutritionally vulnerable older people: A systematic review and meta-analysis.* Clinical Nutrition, 2018. **37**(6): p. 1879-1891.
318. Peters, R., et al., *Orthostatic hypotension and symptomatic subclinical orthostatic hypotension increase risk of cognitive impairment: an integrated evidence review and analysis of a large older adult hypertensive cohort.* Eur Heart J, 2018. **39**(33): p. 3135-3143.
319. Sakashita, T. and H. Oyama, *Overview of community-based studies of depression screening interventions among the elderly population in Japan.* Aging & Mental Health, 2016. **20**(2): p. 231-239.
320. Coronado, R.A., et al., *Pain-Reducing Effects of Physical Therapist-Delivered Interventions: A Systematic Review of Randomized Trials Among Older Adults With Dementia.* Journal of Geriatric Physical Therapy (2001), 2019.
321. Musini, V.M., et al., *Pharmacotherapy for hypertension in adults 60 years or older.* Cochrane Database Syst Rev, 2019. **6**(6): p. CD000028.
322. Thomas, E., et al., *Physical activity programs for balance and fall prevention in elderly: A systematic review.* Medicine (Baltimore), 2019. **98**(27): p. e16218.
323. Lu, J.J., W.J. Fu, and Y. Liu, *Physical activity and cognitive function among older adults in China: A systematic review.* Journal of Sport and Health Science, 2016. **5**(3): p. 287-296.

324. Vancampfort, D., et al., *Physical activity and loneliness among adults aged 50 years or older in six low- and middle-income countries.* International Journal of Geriatric Psychiatry, 2019. **34**(12): p. 1855-1864.
325. Olanrewaju, O., et al., *Physical Activity in Community Dwelling Older People: A Systematic Review of Reviews of Interventions and Context.* PLoS One, 2016. **11**(12): p. e0168614.
326. Larsen, R.T., et al., *Physical activity monitors to enhance amount of physical activity in older adults–a systematic review and meta-analysis.* European Review of Aging and Physical Activity, 2019. **16**(1): p. 7.
327. Larsen, R.T., et al., *Physical activity monitors to enhance the daily amount of physical activity in elderly—a protocol for a systematic review and meta-analysis.* Systematic reviews, 2018. **7**(1): p. 69.
328. Engeroff, T., T. Ingmann, and W. Banzer, *Physical Activity Throughout the Adult Life Span and Domain-Specific Cognitive Function in Old Age: A Systematic Review of Cross-Sectional and Longitudinal Data.* Sports Med, 2018. **48**(6): p. 1405-1436.
329. Chang, P.S., et al., *Physical and Psychological Health Outcomes of Qigong Exercise in Older Adults: A Systematic Review and Meta-Analysis.* Am J Chin Med, 2019. **47**(2): p. 301-322.
330. Bento, C., J. Caçoilo, and I. Calvinho, *Physical exercise in osteoporotic fracture prevention in older adults.* European Geriatric Medicine, 2016. **7**: p. S181.
331. Dinnen, S., V. Simiola, and J.M. Cook, *Post-traumatic stress disorder in older adults: a systematic review of the psychotherapy treatment literature.* Aging Ment Health, 2015. **19**(2): p. 144-50.
332. O'Keeffe, M., et al., *Potentially modifiable determinants of malnutrition in older adults: A systematic review.* Clin Nutr, 2019. **38**(6): p. 2477-2498.
333. Apóstolo, J., et al., *Predicting risk and outcomes for frail older adults: a protocol for an umbrella review of available frailty screening tools.* JBI database of systematic reviews and implementation reports, 2015. **13**(12): p. 14-24.
334. Bohm, S., et al., *Predictive and Reactive Locomotor Adaptability in Healthy Elderly: A Systematic Review and Meta-Analysis.* Sports Med, 2015. **45**(12): p. 1759-77.
335. Tunvirachaisakul, C., et al., *Predictors of treatment outcome in depression in later life: A systematic review and meta-analysis.* J Affect Disord, 2018. **227**: p. 164-182.
336. Fornaro, M., et al., *Prevalence and correlates of major depressive disorder, bipolar disorder and schizophrenia among nursing home residents without dementia: systematic review and meta-analysis.* British Journal of Psychiatry, 2020. **216**(1): p. 6-15.

337. Martinez, F., C. Tobar, and N. Hill, *Preventing delirium: should non-pharmacological, multicomponent interventions be used? A systematic review and meta-analysis of the literature.* Age and ageing, 2015. **44**(2): p. 196-204.
338. Patton, K., C. Soon, and C. Boodram, *Prevention of sexually transmitted and blood borne infections among older adults.* Canadian Journal of Infectious Diseases and Medical Microbiology, 2015. **26**: p. 120B-121B.
339. Briceno, D.F., et al., *Prevention of stroke in the elderly with nonvalvular atrial fibrillation: A systematic review and meta-analysis of randomized controlled trials comparing novel oral anticoagulants and left atrial appendage occlusion device versus warfarin.* Heart Rhythm, 2015. **12**(5): p. S260.
340. Hajat, C., et al., *Preventive Interventions for the Second Half of Life: A Systematic Review.* Am J Health Promot, 2018. **32**(4): p. 1122-1139.
341. Hussenoeder, F.S. and S.G. Riedel-Heller, *Primary prevention of dementia: from modifiable risk factors to a public brain health agenda?* Soc Psychiatry Psychiatr Epidemiol, 2018. **53**(12): p. 1289-1301.
342. van den Brink, R.H.S., et al., *Prognostic significance of social network, social support and loneliness for course of major depressive disorder in adulthood and old age.* Epidemiol Psychiatr Sci, 2018. **27**(3): p. 266-277.
343. Bermeja, A.I. and B. Ausin, *Programs to combat loneliness in the institutionalised elderly: A review of the scientific literature.* Rev Esp Geriatr Gerontol, 2018. **53**(3): p. 155-164.
344. Thomas, D.K., et al., *Protein Supplementation Does Not Significantly Augment the Effects of Resistance Exercise Training in Older Adults: A Systematic Review.* J Am Med Dir Assoc, 2016. **17**(10): p. 959.e1-9.
345. Jonsson, U., et al., *Psychological Treatment of Depression in People Aged 65 Years and Over: A Systematic Review of Efficacy, Safety, and Cost-Effectiveness.* PLoS One, 2016. **11**(8): p. e0160859.
346. McDermott, O., et al., *Psychosocial interventions for people with dementia: a synthesis of systematic reviews.* Aging Ment Health, 2019. **23**(4): p. 393-403.
347. Dugmore, O., M. Orrell, and A. Spector, *Qualitative studies of psychosocial interventions for dementia: a systematic review.* Aging Ment Health, 2015. **19**(11): p. 955-67.
348. Alexiou, K.I., et al., *Quality of life and psychological consequences in elderly patients after a hip fracture: a review.* Clin Interv Aging, 2018. **13**: p. 143-150.
349. Bowling, A., et al., *Quality of life in dementia: a systematically conducted narrative review of dementia-specific measurement scales.* Aging Ment Health, 2015. **19**(1): p. 13-31.

350. Dolatabadi, E., et al., *Quantitative Mobility Assessment for Fall Risk Prediction in Dementia: A Systematic Review.* Dement Geriatr Cogn Disord, 2018. **45**(5-6): p. 353-367.
351. Friedrichs, T., N. Nauta, and E. Fiedler, *Recognizing and Nurturing Giftedness in Gifted Elders.* Parenting for High Potential, 2016. **5**(4): p. 11.
352. Landeiro, F., et al., *Reducing social isolation and loneliness in older people: a systematic review protocol.* BMJ Open, 2017. **7**(5): p. e013778.
353. Aguirre, J.A., *Refresher course: Regional anesthesia to avoid postoperative cognitive dysfunction: What is the evidence?* Regional Anesthesia and Pain Medicine, 2015. **40**(5): p. e36-e38.
354. Backhaus, W., S. Kempe, and F. Hummel, *Rehabilitation with sleep? Results from a systematic review on motor learning and sleep in the aging population and after stroke.* Physiotherapy, 2015. **101**: p. e99-e100.
355. Cui, M., et al., *Relationship between sarcopenia and the incidence of fall in elderly-a systematic review of advanced nutritional and physical interventions studies.* Chinese Journal of Clinical Nutrition, 2017. **25**(5): p. 278-285.
356. Solfrizzi, V., et al., *Relationships of dietary patterns, foods, and micro-and macronutrients with Alzheimer’s disease and late-life cognitive disorders: a systematic review.* Journal of Alzheimer's Disease, 2017. **59**(3): p. 815-849.
357. Schulkes, K.J., et al., *Relevance of a geriatric assessment for elderly patients with lung cancer—a systematic review.* Clinical Lung Cancer, 2016. **17**(5): p. 341-349. e3.
358. Bischoff-Ferrari, H.A., *Relevance of vitamin D in fall prevention.* Geriatr Psychol Neuropsychiatr Vieil, 2017. **15**(1): p. E1-E7.
359. Correa-Perez, A., et al., *Relevant outcomes for nutrition interventions to treat and prevent malnutrition in older people: a collaborative senator-ontop and manuel delphi study.* European Geriatric Medicine, 2018. **9**(2): p. 243-248.
360. Rosen, T., et al., *Review of Programs to Combat Elder Mistreatment: Focus on Hospitals and Level of Resources Needed.* J Am Geriatr Soc, 2019. **67**(6): p. 1286-1294.
361. Feng, Z.Y., et al., *Risk factors and protective factors associated with incident or increase of frailty among community-dwelling older adults: A systematic review of longitudinal studies.* Plos One, 2017. **12**(6): p. e0178383.
362. Smagula, S.F., et al., *Risk factors for sleep disturbances in older adults: Evidence from prospective studies.* Sleep Med Rev, 2016. **25**: p. 21-30.
363. Koh, F., et al., *Role of dietary protein and thiamine intakes on cognitive function in healthy older people: a systematic review.* Nutrients, 2015. **7**(4): p. 2415-39.

364. Ruxton, C.H., E. Derbyshire, and M. Toribio-Mateas, *Role of fatty acids and micronutrients in healthy ageing: a systematic review of randomised controlled trials set in the context of European dietary surveys of older adults.* J Hum Nutr Diet, 2016. **29**(3): p. 308-24.
365. Goisser, S., et al., *Sarcopenic obesity and complex interventions with nutrition and exercise in community-dwelling older persons–a narrative review.* Clinical interventions in aging, 2015. **10**: p. 1267.
366. Duplaga, M., et al., *Scoping review of health promotion and disease prevention interventions addressed to elderly people.* BMC Health Serv Res, 2016. **16 Suppl 5**(5): p. 278.
367. Abdi, J., et al., *Scoping review on the use of socially assistive robot technology in elderly care.* BMJ Open, 2018. **8**(2): p. e018815.
368. Özmete, E. and H.A. Megahead, *Screening for elder abuse among Turkish older people: Validity of the Hwalek–Sengstock elder abuse screening test (HS "East").* Research on social work practice, 2017. **27**(3): p. 387-398.
369. RAČIĆ, M., N. IVKOVIĆ, and S. KUSMUK, *Screening of nutritional status among elderly people at family medicine.* acta medica croatica, 2015. **69**(4): p. 347-356.
370. Magalhaes Junior, H.V., et al., *Screening for oropharyngeal dysphagia in older adults: A systematic review of self-reported questionnaires.* Gerodontology, 2018. **35**(3): p. 162-169.
371. Basu, S., R. Youngs, and A. Mitchell-Innes, *Screening for vestibular schwannoma in the context of an ageing population.* J Laryngol Otol, 2019. **133**(8): p. 640-649.
372. Gallione, C., et al., *Screening tools for identification of elder abuse: a systematic review.* J Clin Nurs, 2017. **26**(15-16): p. 2154-2176.
373. Garnett, A., et al., *Self-Report Tools for Assessing Physical Activity in Community-Living Older Adults with Multiple Chronic Conditions: A Systematic Review of Psychometric Properties and Feasibility.* Can J Aging, 2020. **39**(1): p. 12-30.
374. Song, M., et al., *Seniors centre-based health intervention programmes in the United States and South Korea: A systematic review.* Int J Nurs Pract, 2017. **23**(5): p. e12568.
375. Chen, L.-K. and S.T. Wang, *Seniors' demographic correlates for motivations to enroll in degree-conferring programs in universities.* Educational Gerontology, 2016. **42**(6): p. 431-442.
376. Evans, C.J., et al., *Service Delivery Models to Maximize Quality of Life for Older People at the End of Life: A Rapid Review.* Milbank Quarterly, 2019. **97**(1): p. 113-175.

377. Tam, M., et al., *Similarities and differences in views of ageing and learning in Hong Kong and Australia.* Educational Gerontology, 2017. **43**(8): p. 393-403.
378. Devore, E.E., F. Grodstein, and E.S. Schernhammer, *Sleep Duration in Relation to Cognitive Function among Older Adults: A Systematic Review of Observational Studies.* Neuroepidemiology, 2016. **46**(1): p. 57-78.
379. Liu, L., et al., *Smart homes and home health monitoring technologies for older adults: A systematic review.* Int J Med Inform, 2016. **91**: p. 44-59.
380. Marques Ferreira, M., et al., *Smoking cessation in elderly: Is there a better way?* European Geriatric Medicine, 2016. **7**: p. S183-S184.
381. Coll-Planas, L., et al., *Social capital interventions targeting older people and their impact on health: a systematic review.* Journal of Epidemiology and Community Health, 2017. **71**(7): p. 663-672.
382. Liu, G.D., H. Wang, and Z.G. Huang, *Social Isolation, Chronic Diseases and Prevention in Old Age.* Journal of the American Geriatrics Society, 2019. **67**: p. S663-S663.
383. Santini, Z.I., et al., *Social network typologies and mortality risk among older people in China, India, and Latin America: A 10/66 Dementia Research Group population-based cohort study.* Social Science & Medicine, 2015. **147**: p. 134-143.
384. Read, S., E. Grundy, and E. Foverskov, *Socio-economic position and subjective health and well-being among older people in Europe: a systematic narrative review.* Aging & Mental Health, 2016. **20**(5): p. 529-542.
385. Teng, M., et al., *Statins for Primary Prevention of Cardiovascular Disease in Elderly Patients: Systematic Review and Meta-Analysis.* Drugs Aging, 2015. **32**(8): p. 649-61.
386. Okubo, Y., D. Schoene, and S.R. Lord, *Step training improves reaction time, gait and balance and reduces falls in older people: a systematic review and meta-analysis.* Br J Sports Med, 2017. **51**(7): p. 586-593.
387. Kharicha, K., et al., *Strategies employed by older people to manage loneliness: systematic review of qualitative studies and model development.* Int Psychogeriatr, 2018. **30**(12): p. 1767-1781.
388. Mowszowski, L., et al., *Strategy-Based Cognitive Training for Improving Executive Functions in Older Adults: a Systematic Review.* Neuropsychol Rev, 2016. **26**(3): p. 252-270.
389. Van Oosterwijck, J., et al., *Structural brain plasticity induced by physical training in adults affected by aging or disease related impairments: a systematic review.* Physiotherapy, 2015. **101**: p. e989-e990.
390. Dewansingh, P., et al., *Supplemental protein from dairy products increases body weight and vitamin D improves physical performance in older adults: a*

systematic review and meta-analysis. Nutr Res, 2018. **49**: p. 1-22.

391. Sarode, D.P. and A.K. Demetriades, *Surgical versus nonsurgical management for type II odontoid fractures in the elderly population: a systematic review.* Spine J, 2018. **18**(10): p. 1921-1933.
392. Chang, S.F. and P.L. Lin, *Systematic Literature Review and Meta-Analysis of the Association of Sarcopenia With Mortality.* Worldviews Evid Based Nurs, 2016. **13**(2): p. 153-62.
393. Chastin, S.F., et al., *Systematic literature review of determinants of sedentary behaviour in older adults: a DEDIPAC study.* Int J Behav Nutr Phys Act, 2015. **12**(1): p. 127.
394. Edwards, J.D., et al., *Systematic review and meta-analyses of useful field of view cognitive training.* Neurosci Biobehav Rev, 2018. **84**: p. 72-91.
395. Cheng, H., et al., *Systematic review and meta-analysis of the effect of protein and amino acid supplements in older adults with acute or chronic conditions.* Br J Nutr, 2018. **119**(5): p. 527-542.
396. Nyman, S.R., N. Adamczewska, and N. Howlett, *Systematic review of behaviour change techniques to promote participation in physical activity among people with dementia.* British Journal of Health Psychology, 2018. **23**(1): p. 148-170.
397. Shoshima, A.Y., M.G. Costa, and B.R. Tura, *Systematic Review of Statins Effectiveness in Prevention Secondary in Elderly.* Value in Health, 2017. **20**(9): p. A914-A914.
398. Papadimitriou, A. and M. Perry, *Systematic Review of the Effects of Cognitive and Behavioral Interventions on Fall-Related Psychological Concerns in Older Adults.* Journal of Aging and Physical Activity, 2020. **28**(1): p. 155-168.
399. Morrin, H., et al., *Systematic review of the efficacy of non-pharmacological interventions in people with Lewy body dementia.* Int Psychogeriatr, 2018. **30**(3): p. 395-407.
400. Curtis, A., et al., *Systematic review of the impact of arts for health activities on health, wellbeing and quality of life of older people living in care homes.* Dementia (London), 2018. **17**(6): p. 645-669.
401. Zhao, W.T., et al., *Systematic Review and Meta-Analysis of the Association between Sarcopenia and Dysphagia.* Journal of Nutrition Health & Aging, 2018. **22**(8): p. 1003-1009.
402. Roediger, M.D., et al., *Systematic review of nutritional screening methods for elderly Brazilians living at home.* Ciencia & Saude Coletiva, 2019. **24**(6): p. 2307-2316.

403. Moraes, A.A.I., et al., *Systolic blood pressure target for cardiovascular prevention in the elderly - a meta-analysis of randomized controlled trials.* European Heart Journal, 2016. **37**: p. 1188-1188.
404. Wang, C., D.-C. Seo, and H. Li, *Tai Chi as an Intervention on Health Promotion for Older Adults: A Systematic Review.* Integrative Medicine Research, 2015. **4**(1): p. 117.
405. Du, S., et al., *Taichi exercise for self-rated sleep quality in older people: a systematic review and meta-analysis.* Int J Nurs Stud, 2015. **52**(1): p. 368-79.
406. van den Berg, M. and A. Maeder, *Telehealth in the elderly with chronic heart failure: what is the evidence?* Telehealth for Our Ageing Society: Selected Papers from Global Telehealth 2017, 2018. **246**: p. 18.
407. Mathias, L., et al., *The Application of Virtual Reality in Geriatric Mental Health: The State of the Evidence.* American Journal of Geriatric Psychiatry, 2019. **27**(3): p. S133-S134.
408. Boersma, P., et al., *The art of successful implementation of psychosocial interventions in residential dementia care: a systematic review of the literature based on the RE-AIM framework.* International Psychogeriatrics, 2015. **27**(1): p. 19.
409. Creighton, A.S., T.E. Davison, and D.W. Kissane, *The assessment of anxiety in aged care residents: a systematic review of the psychometric properties of commonly used measures.* International Psychogeriatrics, 2018. **30**(7): p. 967-979.
410. Peel, N.M., et al., *The Association Between Gait Speed and Cognitive Status in Community-Dwelling Older People: A Systematic Review and Meta-analysis.* Journals of Gerontology Series a-Biological Sciences and Medical Sciences, 2019. **74**(6): p. 943-948.
411. Walsh, C.A., et al., *The association between medication non-adherence and adverse health outcomes in ageing populations: A systematic review and meta-analysis.* Br J Clin Pharmacol, 2019. **85**(11): p. 2464-2478.
412. Tirambulo, C., et al. *The Association between Physical Frailty and Polypharmacy in Community Dwelling Older Adults*. in *JOURNAL OF THE AMERICAN GERIATRICS SOCIETY*. 2019. WILEY 111 RIVER ST, HOBOKEN 07030-5774, NJ USA.
413. Kokoaung, E. *The association between SI treatment and suicidality in elderly patients with major depression: A systematic review*. in *INTERNATIONAL PSYCHOGERIATRICS*. 2015. CAMBRIDGE UNIV PRESS 32 AVENUE OF THE AMERICAS, NEW YORK, NY 10013-2473 USA.
414. Smith, G.L., et al., *The association between social support and physical activity in older adults: a systematic review.* International Journal of Behavioral Nutrition and Physical Activity, 2017. **14**(1): p. 56.
415. Wei, J.K., et al., *The association of late-life depression with all-cause and cardiovascular mortality among community-dwelling older adults: systematic*

review and meta-analysis. British Journal of Psychiatry, 2019. **215**(2): p. 449-455.
416. Childs, A., et al., *The burden of respiratory infections among older adults in long-term care: a systematic review.* Bmc Geriatrics, 2019. **19**(1): p. 210.
417. Wu, W.W., et al., *The Effect of a Meditative Movement Intervention on Quality of Sleep in the Elderly: A Systematic Review and Meta-Analysis.* J Altern Complement Med, 2015. **21**(9): p. 509-19.
418. McGrattan, A., et al., *The effect of diet, lifestyle and/or cognitive interventions in mild cognitive impairment: a systematic review.* Proceedings of the Nutrition Society, 2017. **76**(OCE3).
419. Chen, Y.R. and P.J. Schulz, *The Effect of Information Communication Technology Interventions on Reducing Social Isolation in the Elderly: A Systematic Review.* J Med Internet Res, 2016. **18**(1): p. e18.
420. van der Wal-Huisman, H., et al., *The effect of music on postoperative recovery in older patients: A systematic review.* J Geriatr Oncol, 2018. **9**(6): p. 550-559.
421. Fusar-Poli, L., et al., *The effect of music therapy on cognitive functions in patients with dementia: a systematic review and meta-analysis.* Aging Ment Health, 2018. **22**(9): p. 1097-1106.
422. Silva, R.B., et al., *The Effect of Physical Exercise on Frail Older Persons: A Systematic Review.* J Frailty Aging, 2017. **6**(2): p. 91-96.
423. Li, Z., et al., *The effect of resistance training on cognitive function in the older adults: a systematic review of randomized clinical trials.* Aging Clin Exp Res, 2018. **30**(11): p. 1259-1273.
424. Backhaus, W., S. Kempe, and F.C. Hummel, *The effect of sleep on motor learning in the aging and stroke population–a systematic review.* Restorative neurology and neuroscience, 2016. **34**(1): p. 153-164.
425. Aboutorabi, A. and M. Hashemiyari. *The effect of spinal braces on balance of elderly subjects with thoracic hyperkyphosis: Protocol for a systematic review.* in *OSTEOPOROSIS INTERNATIONAL*. 2018. SPRINGER LONDON LTD 236 GRAYS INN RD, 6TH FLOOR, LONDON WC1X 8HL, ENGLAND.
426. Apostolo, J., et al., *The effectiveness of non-pharmacological interventions in older adults with depressive disorders: A systematic review.* International Journal of Nursing Studies, 2016. **58**: p. 59-70.
427. Zahari, Z., A. Ishak, and M. Justine, *The effectiveness of patient education in improving pain, disability and quality of life among older people with low back pain: A systematic review.* Journal of Back and Musculoskeletal Rehabilitation, 2020. **33**(2): p. 245-254.
428. Ramis, M.A., et al., *The effectiveness of peer-based interventions on health promoting behaviors in older people: a systematic review protocol of*

quantitative evidence. JBI Database System Rev Implement Rep, 2015. **13**(9): p. 177-86.
429. Tai, K., I.M. Batista de Souza, and A.P. Marques, *The effectiveness of Tai Chi Chuan in osteoarthritis of the knee: A systematic review.* Annals of the Rheumatic Diseases, 2019. **78**(Suppl 2): p. 1060-1061.
430. Lim, K.H., et al., *The effectiveness of Tai Chi for short-term cognitive function improvement in the early stages of dementia in the elderly: a systematic literature review.* Clin Interv Aging, 2019. **14**: p. 827-839.
431. Silva, P.A., A. Cochrane, and H. Farrell, *The Effectiveness of Technology-Mediated Dance Interventions and Their Impact on Psychosocial Factors in Older Adults: A Systematic Review and Meta-Analysis.* Games for Health Journal, 2018. **7**(6): p. 347-361.
432. Zhang, Y., et al., *The Effects of Mind-Body Exercise on Cognitive Performance in Elderly: A Systematic Review and Meta-Analysis.* Int J Environ Res Public Health, 2018. **15**(12): p. 2791.
433. Guitar, N.A., et al., *The effects of physical exercise on executive function in community-dwelling older adults living with Alzheimer's-type dementia: a systematic review.* Ageing research reviews, 2018. **47**: p. 159-167.
434. Bullo, V., et al., *The effects of Pilates exercise training on physical fitness and wellbeing in the elderly: A systematic review for future exercise prescription.* Prev Med, 2015. **75**: p. 1-11.
435. Pan, M.X., et al., *The effects of Qigong exercises on blood lipid profiles of middle-aged and elderly individuals: A systematic review and network meta-analysis.* European Journal of Integrative Medicine, 2019. **30**: p. 100950.
436. Engers, P.B., et al., *The effects of the Pilates method in the elderly: a systematic review.* Revista Brasileira De Reumatologia, 2016. **56**(4): p. 352-365.
437. Moreno-Segura, N., et al., *The Effects of the Pilates Training Method on Balance and Falls of Older Adults: A Systematic Review and Meta-Analysis of Randomized Controlled Trials.* J Aging Phys Act, 2018. **26**(2): p. 327-344.
438. Vetrovsky, T., et al., *The Efficacy and Safety of Lower-Limb Plyometric Training in Older Adults: A Systematic Review.* Sports Med, 2019. **49**(1): p. 113-131.
439. Jeong Hong, Y., et al., *The efficacy of cognitive intervention programs for mild cognitive impairment: a systematic review.* Current Alzheimer Research, 2015. **12**(6): p. 527-542.
440. Bragina, I. and C. Voelcker-Rehage, *The exercise effect on psychological well-being in older adults—a systematic review of longitudinal studies.* German Journal of Exercise and Sport Research, 2018. **48**(3): p. 323-333.
441. O'Brien, N., et al., *The features of interventions associated with long-term effectiveness of physical activity interventions in adults aged 55–70 years: a*

systematic review and meta-analysis. Health psychology review, 2015. **9**(4): p. 417-433.
442. Couture, M., A. Giguère-Rancourt, and M. Simard, *The impact of cognitive interventions on cognitive symptoms in idiopathic Parkinson's disease: a systematic review.* Aging, Neuropsychology, and Cognition, 2019. **26**(5): p. 637-659.
443. Nowson, C.A., et al., *The Impact of Dietary Factors on Indices of Chronic Disease in Older People: A Systematic Review.* J Nutr Health Aging, 2018. **22**(2): p. 282-296.
444. Tieland, M., et al., *The impact of dietary protein or amino acid supplementation on muscle mass and strength in elderly people: individual participant data and meta-analysis of RCT's.* The journal of nutrition, health & aging, 2017. **21**(9): p. 994-1001.
445. Hudes, R., et al., *The impact of memory-strategy training interventions on participant-reported outcomes in healthy older adults: A systematic review and meta-analysis.* Psychol Aging, 2019. **34**(4): p. 587-597.
446. Rondón García, L.M. and J.M. Ramírez Navarrro, *The impact of quality of life on the health of older people from a multidimensional perspective.* Journal of aging research, 2018. **2018**.
447. Jessen-Winge, C., M.N. Petersen, and A.-L. Morville, *The influence of occupation on wellbeing, as experienced by the elderly: a systematic review.* JBI database of systematic reviews and implementation reports, 2018. **16**(5): p. 1174-1189.
448. Siskind, D.J., et al., *The Mental Health of Older Persons After Human-Induced Disasters: A Systematic Review and Meta-Analysis of Epidemiological Data.* Am J Geriatr Psychiatry, 2016. **24**(5): p. 379-88.
449. Cerin, E., et al., *The neighbourhood physical environment and active travel in older adults: a systematic review and meta-analysis.* Int J Behav Nutr Phys Act, 2017. **14**(1): p. 15.
450. Dhawan, N., J. Core, and M. Khalili, *The occurence, risk factors, and prevention of contrast-induced nephropathy in the elderly.* Journal of the American Geriatrics Society, 2017. **65**: p. S215.
451. Liang, J.H., et al., *The optimal treatment for improving cognitive function in elder people with mild cognitive impairment incorporating Bayesian network meta-analysis and systematic review.* Ageing Res Rev, 2019. **51**: p. 85-96.
452. Klugar, M., et al., *The personal active aging strategies of older adults in Europe: a systematic review of qualitative evidence.* JBI Database System Rev Implement Rep, 2016. **14**(5): p. 193-257.
453. Wong, R., et al., *The relationship between sarcopenia and fragility fracture—a systematic review.* Osteoporosis International, 2019. **30**(3): p. 541-553.

454. Clegg, M.E. and A. Godfrey, *The relationship between physical activity, appetite and energy intake in older adults: A systematic review.* Appetite, 2018. **128**: p. 145-151.

455. Chen, S., H. Brodaty, and F. O'Leary. *The relationship of nutrition to cognitive function in older adults: a systematic review of RCTs*. in *INTERNATIONAL PSYCHOGERIATRICS*. 2015. CAMBRIDGE UNIV PRESS 32 AVENUE OF THE AMERICAS, NEW YORK, NY 10013-2473 USA.

456. Liao, C.-D., et al., *The Role of Muscle Mass Gain Following Protein Supplementation Plus Exercise Therapy in Older Adults with Sarcopenia and Frailty Risks: A Systematic Review and Meta-Regression Analysis of Randomized Trials.* Nutrients, 2019. **11**(8): p. 1713.

457. Hazavehei, S.M. and M. Afshari, *The role of nutritional interventions in increasing fruit and vegetable intake in the elderlies: a systematic review.* Aging Clin Exp Res, 2016. **28**(4): p. 583-98.

458. Dove, E. and A.J. Astell, *The Use of Motion-Based Technology for People Living With Dementia or Mild Cognitive Impairment: A Literature Review.* Journal of Medical Internet Research, 2017. **19**(1): p. e3.

459. Ramprasad, C., et al., *The use of tablet technology by older adults in health care settings—is it effective and satisfying? a systematic review and meta analysis.* Clinical gerontologist, 2019. **42**(1): p. 17-26.

460. Harerimana, B., C. Forchuk, and T. O'Regan, *The use of technology for mental healthcare delivery among older adults with depressive symptoms: A systematic literature review.* International Journal of Mental Health Nursing, 2019. **28**(3): p. 657-670.

461. Palop Montoro, M.V., et al., *[THE VIBRATION TRAINING AS SARCOPENIA INTERVENTION: IMPACT ON THE NEUROMUSCULAR SYSTEM OF THE ELDERLY].* Nutr Hosp, 2015. **32**(4): p. 1454-61.

462. Nicholas, S.O., A.T. Giang, and P.L.K. Yap, *The Effectiveness of Horticultural Therapy on Older Adults: A Systematic Review.* J Am Med Dir Assoc, 2019. **20**(10): p. 1351 e1-1351 e11.

463. Kraicer-Melamed, H., S. O'Donnell, and C. Quach, *The effectiveness of pneumococcal polysaccharide vaccine 23 (PPV23) in the general population of 50 years of age and older: A systematic review and meta-analysis.* Vaccine, 2016. **34**(13): p. 1540-1550.

464. Marinus, N., et al., *The Impact of Different Types of Exercise Training on Peripheral Blood Brain-Derived Neurotrophic Factor Concentrations in Older Adults: A Meta-Analysis.* Sports Med, 2019. **49**(10): p. 1529-1546.

465. Patton, D.E., et al., *Theory-Based Interventions to Improve Medication Adherence in Older Adults Prescribed Polypharmacy: A Systematic Review.* Drugs Aging, 2017. **34**(2): p. 97-113.

466. Joseph, K.R., S. Edirimanne, and G.D. Eslick, *Thyroidectomy for thyroid cancer in the elderly: A meta-analysis.* Eur J Surg Oncol, 2019. **45**(3): p. 310-317.
467. Otuyama, L.J., et al., *Tobacco smoking and risk for dementia: evidence from the 10/66 population-based longitudinal study.* Aging Ment Health, 2019: p. 1-11.
468. Cerutti-Kopplin, D., et al., *Tooth Loss Increases the Risk of Diminished Cognitive Function: A Systematic Review and Meta-analysis.* JDR Clin Trans Res, 2016. **1**(1): p. 10-19.
469. Kojima, G., et al., *Transitions between frailty states among community-dwelling older people: A systematic review and meta-analysis.* Ageing Research Reviews, 2019. **50**: p. 81-88.
470. Lely, J.C.G., *Treating trauma-related disorders in later life*. 2019, Utrecht University.
471. Haywood, C. and P. Sumithran, *Treatment of obesity in older persons—A systematic review.* Obesity Reviews, 2019. **20**(4): p. 588-598.
472. Gil-Montoya, J.A., et al., *Treatment of xerostomia and hyposalivation in the elderly: A systematic review.* Med Oral Patol Oral Cir Bucal, 2016. **21**(3): p. e355-66.
473. Dominguez-Chavez, C.J., B.C. Salazar-Gonzalez, and C.J. Murrock, *Use of Music Therapy to Improve Cognition in Older Adults With Dementia: An Integrative Review.* Research and Theory for Nursing Practice, 2019. **33**(2): p. 183-195.
474. Scoglio, A.A., et al., *Use of Social Robots in Mental Health and Well-Being Research: Systematic Review.* J Med Internet Res, 2019. **21**(7): p. e13322.
475. Abdi, J., A. Al-Hindawi, and M. Vizcaychipi. *Use of socially assistive robot technology in elderly care.* in *AGE AND AGEING*. 2017. OXFORD UNIV PRESS GREAT CLARENDON ST, OXFORD OX2 6DP, ENGLAND.
476. Heinzel, S., et al., *Using Exercise to Fight Depression in Older Adults A Systematic Review and Meta-Analysis.* Geropsych-the Journal of Gerontopsychology and Geriatric Psychiatry, 2015. **28**(4): p. 149-162.
477. Corbett, T., et al. *Using the cumulative model of patient complexity to understand supported self-management in older people living with multi-morbidity and cancer: A theory-led qualitative systematic review*. in *PSYCHO-ONCOLOGY*. 2019. WILEY 111 RIVER ST, HOBOKEN 07030-5774, NJ USA.
478. Setbo, E., et al., *Utility of probiotics for maintenance or improvement of health status in older people—a scoping review.* The journal of nutrition, health & aging, 2019. **23**(4): p. 364-372.
479. de Amorim, J.S.C., et al., *Virtual reality therapy for rehabilitation of balance in the elderly: a systematic review and META-analysis.* Adv Rheumatol, 2018. **58**(1): p. 18.

480. McCleery, J., et al., *Vitamin and mineral supplementation for preventing dementia or delaying cognitive decline in people with mild cognitive impairment.* Cochrane Database Syst Rev, 2018. **11**(11): p. CD011905.
481. Oliveira, J.S., et al., *What is the effect of health coaching on physical activity participation in people aged 60 years and over? A systematic review of randomised controlled trials.* British Journal of Sports Medicine, 2017. **51**(19): p. 1425-1432.
482. Ronzi, S., et al., *What is the impact on health and wellbeing of interventions that foster respect and social inclusion in community-residing older adults? A systematic review of quantitative and qualitative studies.* Systematic reviews, 2018. **7**(1): p. 26.
483. Huston, P. and B. McFarlane, *What to say if your patients ask about tai chi: Evidence-based response.* Canadian Family Physician, 2017. **63**(2): p. S35.
484. Aucella, F., et al. *Which interventions are effective in improving nutritional status in elderly/frail patients with CKD stage 3B-5D?: a preliminary report by EBPG group*. in *53rd ERA-EDTA Congress*. 2016.
485. Ruxton, C. and E. Derbyshire, *Which nutrients may contribute to healthy aging? A systematic review.* Proceedings of the Nutrition Society, 2016. **75**(OCE3).
486. Tulloch, A., et al., *Yoga-based exercise improves health-related quality of life and mental well-being in older people: a systematic review of randomised controlled trials.* Age Ageing, 2018. **47**(4): p. 537-544.

Supplementary 4

Table of comparison between the current study and Duplaga et al,2016

	current study	Duplaga et al
Aim of study	(1) To investigate the distribution of and trend in the literature on health promotion in the elderly over a recent five year period.<br>(2) To explore the use and contribution of technology in the literature on health promotion in the elderly.<br>(3) To make suggestions for the development of health policies for the elderly in the future.	Identification and classification of health promotion and related types of interventions addressing general health issues as well as those specific to ageing among older adults and elderly people.
Final number of result	486	334
Publication year	2015/01-2019/10	2000/01-2015/04
Database	Pubmed,<br>CINAHL,<br>the CochraneLibrary,<br>EMBASE,<br>PubPsych,<br>ERIC (EBSCOhost)	MEDLINE,<br>CINAHL,<br>the CochraneLibrary,<br>EMBASE,<br>PubPsych,<br>ERIC,<br>INSPEC
Top 3 target areas of interventions	disease-oriented (n = 214)<br>physical activity (n = 120)<br>general health (n = 118),	disease oriented(n=132)<br>physical activity(n=94)<br>general health (n=82)

Top 3 categories of general area of interventions	Health Promotion (n=118, 24.3%)<br>Health Promotion & Screening (n=99, 20.4%)<br>Screening (n=94, 19.3%)	Primary Prevention (n=112, 33.5%)<br>Health Promotion & Primary Prevention (n=79, 23.7%)<br>Health Promotion (n=75, 22.5%)
Specific gender target	elderly females (n=10)	woman (n=26)<br>men (n=7)
Classification difference	eHealth technology categories	health promotion interventions classified according to the McKenzie et al. taxonomy
